# Intracerebroventricular B7-H3-targeting CAR T cells for diffuse intrinsic pontine glioma: a phase 1 trial

**DOI:** 10.1038/s41591-024-03451-3

**Published:** 2025-01-07

**Authors:** Nicholas A. Vitanza, Rebecca Ronsley, Michelle Choe, Kristy Seidel, Wenjun Huang, Stephanie D. Rawlings-Rhea, Madison Beam, Leonel Steinmetzer, Ashley L. Wilson, Christopher Brown, Adam Beebe, Catherine Lindgren, Joshua A. Gustafson, Amy Wein, Susan Holtzclaw, Corrine Hoeppner, Hannah E. Goldstein, Samuel R. Browd, Jason S. Hauptman, Amy Lee, Jeffrey G. Ojemann, Erin E. Crotty, Sarah E. S. Leary, Francisco A. Perez, Jason N. Wright, Marta M. Alonso, Matthew D. Dun, Jessica B. Foster, Diana Hurst, Ada Kong, Alison Thomsen, Rimas J. Orentas, Catherine M. Albert, Navin Pinto, Colleen Annesley, Rebecca A. Gardner, On Ho, Sowmya Pattabhi, Juliane Gust, Jason P. Wendler, Julie R. Park, Michael C. Jensen

**Affiliations:** 1 Ben Towne Center for Childhood Cancer and Blood Disorders Research, Seattle Children’s Research Institute, Seattle, WA USA; 2 Department of Pediatrics, Seattle Children’s Hospital, University of Washington, Seattle, WA USA; 3 Seattle Children’s Therapeutics, Seattle, WA USA; 4 Division of Neurosurgery, Seattle Children’s Hospital and Department of Neurological Surgery, University of Washington, Seattle, WA USA; 5 Center for Integrative Brain Research, Seattle Children’s Research Institute, Seattle, WA USA; 6 Department of Radiology, Seattle Children’s Hospital, Seattle, WA USA; 7 Department of Pediatrics, Program of Solid Tumors, University Clinic of Navarra, CIMA-Universidad de Navarra, Pamplona, Spain; 8 Cancer Signalling Research Group, School of Biomedical Sciences and Pharmacy, College of Health, Medicine and Wellbeing, University of Newcastle, Newcastle, New South Wales Australia; 9 Precision Medicine Research Program, Hunter Medical Research Institute, Newcastle, New South Wales Australia; 10 Paediatric Stream, Mark Hughes Foundation Centre for Brain Cancer Research, College of Health, Medicine and Wellbeing, Newcastle, New South Wales Australia; 11 Division of Oncology, Children’s Hospital of Philadelphia, Philadelphia, PA USA; 12 Department of Pediatrics, University of Pennsylvania Perelman School of Medicine, Philadelphia, PA USA; 13 Department of Pharmacy, Seattle Children’s Hospital, Seattle, WA USA; 14 Division of Pediatric Neurology, Department of Neurology, University of Washington, Seattle, WA USA; 15 Department of Oncology, St. Jude Children’s Research Hospital, Memphis, TN USA

**Keywords:** CNS cancer, Cancer immunotherapy, Paediatric cancer

## Abstract

Diffuse intrinsic pontine glioma (DIPG) is a fatal central nervous system (CNS) tumor that confers a median survival of 11 months. As B7-H3 is expressed on pediatric CNS tumors, we conducted BrainChild-03, a single-center, dose-escalation phase 1 clinical trial of repetitive intracerebroventricular (ICV) dosing of B7-H3-targeting chimeric antigen receptor T cells (B7-H3 CAR T cells) for children with recurrent or refractory CNS tumors and DIPG. Here we report results from Arm C, restricted to patients with DIPG. The primary objectives were to assess feasibility and tolerability, which were both met. Secondary objectives included assessments of CAR T cell distribution and survival. A total of 23 patients with DIPG enrolled, and 21 were treated with repeated doses of ICV B7-H3 CAR T cells using intra-patient dose-escalation regimens without previous lymphodepletion. Concurrent tumor-directed therapy, including re-irradiation, was not allowed while on protocol therapy. We delivered a total of 253 ICV doses and established the highest planned dose regimen, DR4, which escalated up to 10 × 10^7^ cells per dose, as the maximally tolerated dose regimen. Common adverse events included headache, fatigue and fever. There was one dose-limiting toxicity (intratumoral hemorrhage) during DR2. For all treated patients (*n* = 21), the median survival from their initial CAR T cell infusion was 10.7 months and the median survival from diagnosis was 19.8 months with 3 patients still alive at 44, 45 and 52 months from diagnosis. Ultimately, this completed first-in-human trial shows that repetitive ICV dosing of B7-H3 CAR T cells in pediatric and young adult patients with DIPG is tolerable, including multiyear repeated dosing, and may have clinical efficacy that warrants further investigation on a multisite phase 2 trial. ClinicalTrials.gov registration: NCT04185038.

## Abstract (web summary)

In the final report of a phase 1 trial evaluating intracerebroventricular B7-H3-targeting CAR T cells in children and young adults with diffuse intrinsic pontine glioma, repeated intracranial infusions were feasible and well tolerated with a median overall survival of 19.8 months and 3 patients surviving over 40 months from diagnosis.

## Main

Diffuse intrinsic pontine glioma (DIPG) is a fatal brainstem tumor responsible for ~25,000 years of life lost each year in the United States^1,2^. Chimeric antigen receptor (CAR) T cells have had substantial efficacy against pediatric leukemia^3,4^, yet the clinical application of intracranial CAR T cells for children with DIPG is a nascent area of therapeutic development^5–10^.

Our initial phase 1 clinical trials delivering intracranial CAR T cells (BrainChild-01 delivering human epidermal growth factor receptor 2 (HER2)-specific CAR T cells (NCT03500991)^11^; BrainChild-02 delivering EGFR806-specific CAR T cells^12^ (NCT03638167)) excluded patients with DIPG. As B7-H3 (CD276) is expressed on DIPG^13–16^, we designed B7-H3-targeting CAR T cells (B7-H3 CAR T cells) and described their preclinical efficacy^6^. Next, we opened BrainChild-03 (NCT04185038), delivering B7-H3 CAR T cells to children with recurrent or refractory central nervous system (CNS) tumors and DIPG, and published on the preliminary tolerability^6^.

Here we report on the completed Arm C of the first-in-human phase 1 clinical trial BrainChild-03 dedicated to children with DIPG. Patients were enrolled at any time following standard radiotherapy (including patients with disease progression and/or metastases). We show the safety of repetitive intracerebroventricularly (ICV) dosed B7-H3 CAR T cells up to 10 × 10^7^ cells per dose to children with DIPG, evidence of immune activation in the CNS and the potential clinical benefit.

## Results

### Study design and patient characteristics

This was a phase 1 study of repeatedly dosed ICV adoptive cell therapy with autologous CD4^+^ and CD8^+^ T cells lentivirally transduced to express a B7-H3-specific CAR to children and young adults with DIPG. The primary objectives were to assess the feasibility, safety and tolerability of ICV B7-H3 CAR T cell therapy, while the secondary objectives included assessments of CAR T cell distribution and survival. Eligibility criteria included the following: age ≥1 and ≤26 years, a diagnosis of DIPG (diagnosed radiographically or via histopathologic confirmation of high-grade glioma or diffuse midline glioma H3K27M-altered (DMG)) at any timepoint following completion of standard radiation, presence of a CNS catheter, Lansky or Karnofsky performance of ≥60, dexamethasone dose ≤2.5 mg m^−2^ d^−1^, protocol-defined washout from previous therapies and protocol-defined laboratory evidence of adequate organ function (including an absolute lymphocyte count of ≥100 cells µl^−1^).

**Extended Data Fig. 1:**
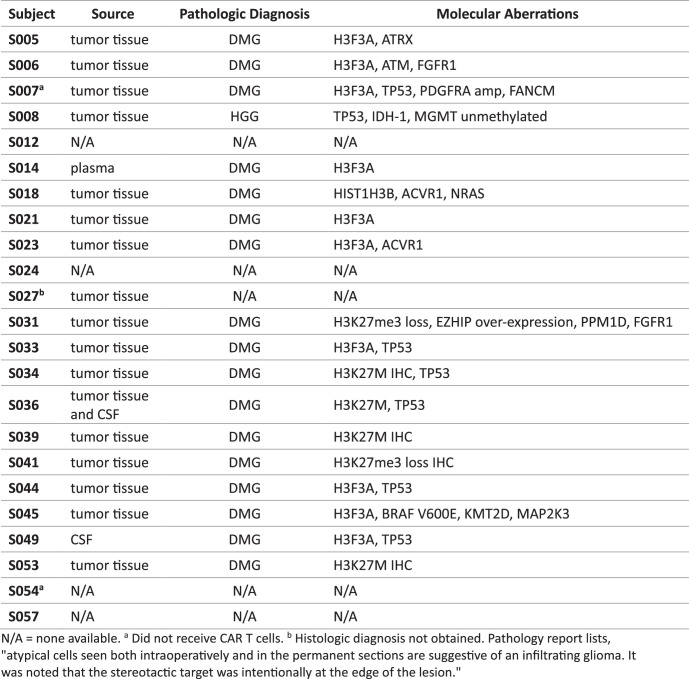
Pathologic diagnostic information for all patients.

The median age at enrollment of 6 years (range: 2–22 years) is consistent with the historical median age for this disease^1^. Patients had Lansky or Karnofsky performance scores of 60 (2, 9%), 70 (2, 9%), 80 (7, 30%) and 90 (12, 52%). All patients received standard radiation therapy at diagnosis. The two enrolled patients who did not receive CAR T cells each experienced clinical progression during CAR T cell manufacturing and never met eligibility for CAR T cell infusion. Of the 21 treated patients, 12 (57%) received treatment after at least one tumor progression, while 9 patients (43%) were treated before any tumor progression. The primary objective of the feasibility to manufacture CAR T cells from a single apheresis was met for all enrollees. The baseline characteristics of the patients are provided in Table 1. Of the 21 treated patients, 17 met the molecular diagnosis of DMG either by tumor tissue (*n* = 15) or by CSF or plasma testing (*n* = 2) (Extended Data Fig. 1). One patient (S008) had a pontine anaplastic astrocytoma harboring an *IDH1* mutation.

**Table 1:** Demographics and clinical characteristics of study participants enrolled in BrainChild-03 Arm C (*n* = 23)

Characteristics	Value
Median age (years (range))	6 (2–22)
Sex (*n* (%))	
Male	9 (39)
Female	14 (61)
Lansky or Karnofsky performance status score (*n* (%))	
90	12 (52)
80	7 (30)
70	2 (9)
60	2 (9)
Median months from diagnosis to enrollment (range)	6 (3–22)
Histopathologic or molecular confirmation of DMG (*n* (%))	18 (78)
Disease history at enrollment (*n* (%))	
Previous progression	12 (52)
No previous progression	11 (48)
Disease history at first dose of B7-H3 CAR (*n* (%); total number treated: 21)	
Previous progression	12 (57)
No previous progression	9 (43)
Previous radiation therapy (*n* (%))	23 (100)

### Treatment

**Fig. 1:**
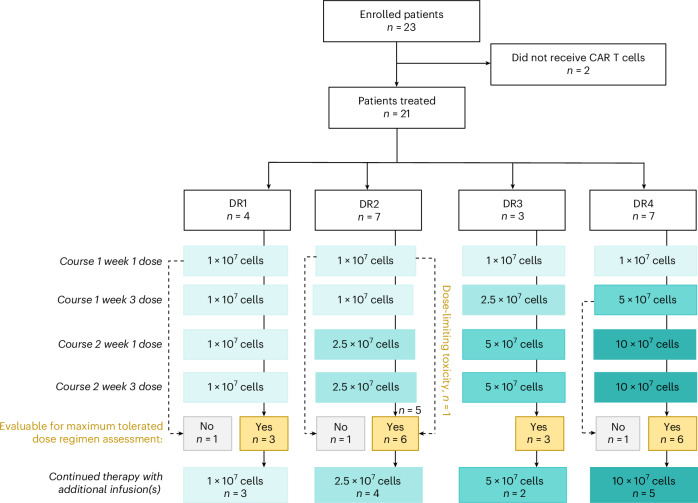
BrainChild-03 Arm C trial design. CONSORT diagram of BrainChild-03 Arm C.

BrainChild-03 Arm C delivered ICV B7-H3 CAR T cells without previous lymphodepletion every 14 days over 8 weeks during the dose-limiting toxicity (DLT) observation period (courses 1 and 2). Concurrent tumor-directed therapy, including re-irradiation, was not allowed while on protocol therapy. As the optimal number of doses is unknown, patients—who met criteria for subsequent infusions beyond course 2 (and for whom additional cryopreserved CAR T cell doses were available)—were eligible to continue receiving CAR T cell therapy beyond the DLT period with doses every 2–4 weeks. Patients were treated on four escalating dose regimens (DRs; Fig. 1). In DR1, three evaluable patients received 1 × 10^7^ CAR T cells per dose. DR2, DR3 and DR4 used intra-patient dose escalation. In DR2, 6 evaluable patients received up to 2.5 × 10^7^ CAR T cells per dose; in DR3, 3 patients received up to 5 × 10^7^ CAR T cells per dose and, in DR4, 6 evaluable patients received up to 10 × 10^7^ CAR T cells per dose. DR2 expanded to six patients owing to a DLT (intratumoral hemorrhage), and DR4 expanded to six patients for a planned statistical confirmation of the maximally tolerated dose regimen (MTDR). Three patients were not DLT evaluable (as they elected to pursue other tumor-directed therapy out of concern for progressive disease (PD) without having experienced a DLT) and were replaced as per the protocol.

**Fig. 2:**
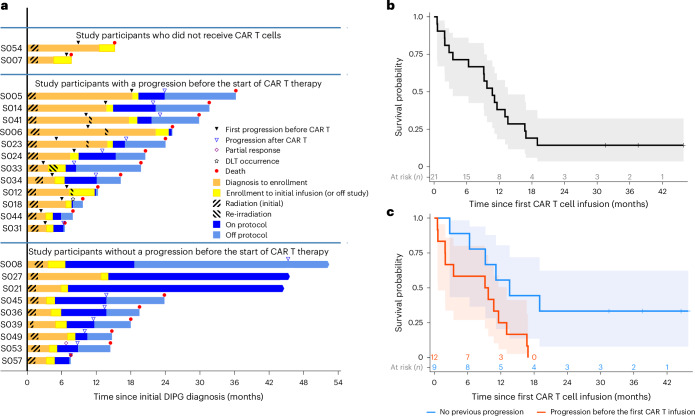
Survival following intracranial B7-H3 CAR T cells. **a**, Swimmer plot describing patient history from time of diagnosis through death or most recent follow-up. For patients with multiple progressions or long-standing ongoing progression, only initial progression may be noted. **b**, Kaplan–Meier survival after the initial B7-H3 CAR T cell infusion for all treated patients. **c**, Kaplan–Meier survival stratified by previous progression status at the time of initial CAR T infusion. Shaded areas in **b** and **c** denote 95% confidence limits for the Kaplan–Meier estimates.

For patients who began treatment before any progression (*n* = 9), the median time from diagnosis to initial CAR T cell infusion was 6.6 months (range: 4.8–14.1; Fig. 2a). For patients who began treatment after progression (*n* = 12), the median time from initial diagnosis to initial CAR T cell infusion was 10.3 months (range: 4.5–24.6 months). For all patients receiving at least one CAR T cell infusion (*n* = 21), the median interval between enrollment and initial CAR T cell dose was 1.4 months (range: 1.0–4.4 months). The 18 patients evaluable for DLT received 253 total doses (median: 9 per patient; range: 1–81) and remained on protocol therapy for a median of 5 months (range: 0.2–37.5 months).

### Safety

All patients who received at least one CAR T infusion are included in the adverse event (AE) summary (*n* = 21; Table 2). The most frequent AEs that were possibly, probably or definitely attributable to CAR T therapy were headache (17 patients, 81%), nausea or vomiting (17 patients, 81%), fatigue (13 patients, 62%) and fever (12 patients, 57%). Most events were grade 1 or 2 with few patients experiencing grade 3 toxicities. Only one patient developed hydrocephalus (grade 3). Fever was considered evidence of local immune activation rather than conventional systemic cytokine release syndrome (CRS). Immune-effector-cell-associated neurotoxicity syndrome was not observed. The sole DLT was an intratumoral hemorrhage (grade 4) occurring in a 3 year old with PD between enrollment and initial infusion. They received their first CAR T cell dose (dose level (DL) 1 of DR2), then had a stable neurologic exam and unchanged performance score without evidence of systemic inflammation (including normal serum C-reactive protein (CRP), ferritin, d-dimer, interleukin (IL)-2, IL-6 and interferon-γ (IFNγ)) for 1 week before the acute event. Following the hemorrhage, they required admission to the pediatric intensive care unit followed by slow, limited neurologic improvement, but ultimately had fatal progression of their tumor.

**Table 2:** AEs recorded during the DLT observation period

Event	Grade 1–2, *n* (%)	Grade 3, *n* (%)	Grade 4, *n* (%)
Neurologic			
Headache	15 (71)	2 (10)	0
Ataxia	3 (14)	1 (5)	0
Dizziness	2 (10)	0	0
Dysarthria	1 (5)	0	0
Dysphagia	1 (5)	0	0
Facial nerve disorder	2 (10)	0	0
Hydrocephalus	0	1 (5)	0
Unilateral muscle weakness	2 (10)	2 (10)	0
Paresthesia	4 (19)	0	0
Peripheral sensory neuropathy	1 (5)	0	0
Tremor	1 (5)	0	0
Trochlear nerve disorder	1 (5)	0	0
Ophthalmic			
Diplopia	1 (5)	0	0
Photophobia	2 (10)	0	0
Gastrointestinal			
Nausea	12 (57)	1 (5)	0
Vomiting	15 (71)	1 (5)	0
Anorexia	3 (14)	0	0
Abdominal pain	1 (5)	0	0
Dysphagia	1 (5)	0	0
Tumor related			
Tumor hemorrhage	0	0	1 (5)
Constitutional			
Fever	11 (52)	1 (5)	0
Fatigue	13 (62)	0	0
Malaise	1 (5)	0	0
Pain	2 (10)	0	0
Agitation	1 (5)	0	0
Respiratory			
Dyspnea	1 (5)	0	0
Hiccups	6 (29)	0	0
Cardiovascular			
Hypertension	1 (5)	0	0
Hypotension	1 (5)	0	0
Musculoskeletal			
Back pain	1 (5)	0	0
Neck pain	3 (14)	1 (5)	0
Urinary			
Incontinence	1 (5)	0	0
Dermatologic			
Pruritus	1 (5)	0	0
Hematologic			
Lymphocytopenia	1 (5)	0	0

CTCAE grading of all observed toxicities during the DLT observation.

### Clinical outcomes

Some clinical trials for DIPG mandate enrollment at diagnosis, while others allow patients to enroll anytime following standard radiation. The latter risks an immortalization bias in which some patients have a prolonged period before enrollment that constitutes a substantial portion of their overall survival. Therefore, while we calculated the median survival from initial diagnosis, we also evaluated the survival from study enrollment (for all enrolled patients) and from initial CAR T cell infusion (for all treated patients). Patients were followed from the time of enrollment, which occurred between August 2020 and April 2023, through treatment until death or censoring at their last follow-up visit before 13 November 2024.

For all enrolled patients (*n* = 23), the median survival from the time of study enrollment was 11.4 months (range: 2.7–48.8 months) and the median survival from diagnosis to death (or last contact for survivors) was 19.5 months (range: 6.5–52.5 months; Fig. 2a). For all treated patients (*n* = 21), the median survival from their initial CAR T cell infusion was 10.7 months (range: 0.6–45.8 months; Fig. 2b) and the median time from diagnosis to death (or last contact for survivors) was 19.8 months with 3 patients still alive 44.6, 45.6 and 52.5 months from diagnosis (range: 6.5–52.5 months; Fig. 2b).

As it is unknown whether intracranial cellular therapy delivered temporally near initial radiation may cause added toxicity or, conversely, be more efficacious when disease burden may be lowest, we performed a post hoc analysis comparing patients who enrolled before progression (*n* = 9) with those who enrolled after progression (*n* = 12). For patients who began CAR T treatment after progression (*n* = 12), the median survival following their initial CAR T cell infusion was 9.4 months (range: 0.6–16.9 months; Fig. 2c) and the median survival from diagnosis was 20.1 months (range: 6.5–36.2 months). For patients who began treatment before progression (*n* = 9), the median survival following their initial CAR T cell infusion was 13.6 months (range: 2.8–45.8 months; Fig. 2c) and the median time from diagnosis to death (or last contact for survivors) was 19.5 months (range: 7.6–52.5 months). All three surviving patients were treated before progression. One surviving patient is in long-term follow-up: S008 (pontine high-grade glioma with mutations in *TP53* and *IDH1* diagnosed at 22 years of age) left protocol therapy owing to patient preference after 1 year of treatment. Two surviving patients remain on protocol therapy: S021 (pontine DMG with a *H3F3A* K27M mutation diagnosed at 2 years of age) has received 81 doses (3 × 10^9^ total CAR T cells) over 37.5 months, and S027 (non-diagnostic biopsy at 5 years of age) has received 39 doses (9.5 × 10^8^ total CAR T cells) over 31.5 months.

**Fig. 3:**
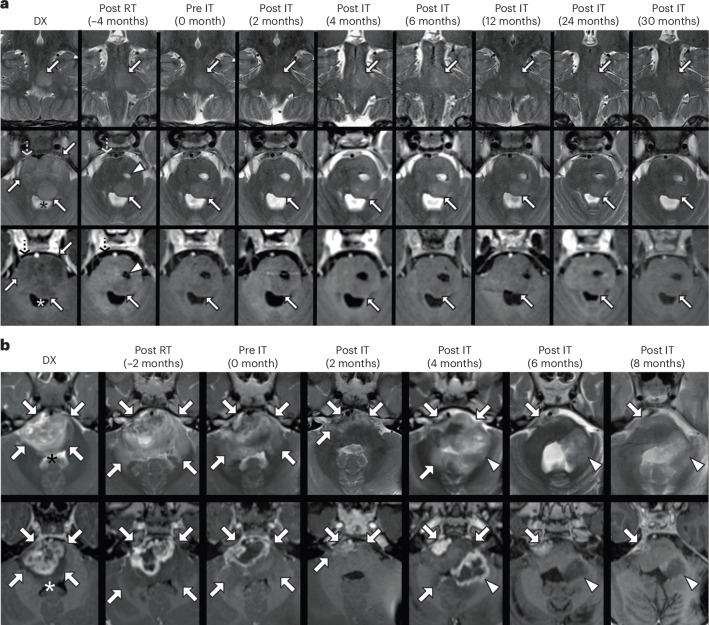
Neuroimaging after locoregional B7-H3 CAR T cell infusion. **a**, Longitudinal MRIs of S021. T2-weighted (coronal top row, axial middle row) and axial post-contrast T1-weighted (bottom row) MRI images focused on the pontine lesion at various timepoints (see column labels). At diagnosis (DX), the pons is expanded with non-enhancing T2 hyperintensity, prepontine cistern effacement (dashed arrows) and partial effacement of the fourth ventricle (asterisks). Following radiation (Post RT, 4 months before immunotherapy (IT)), the lesion is smaller, with reduced prepontine cistern effacement (dashed arrows), persistent nodular T2 hyperintensity in the left dorsal pons (solid arrows) and biopsy- and therapy-related changes in the central pons (arrowheads). From the initiation of IT (Pre IT) to 30 months Post IT, the overall pons size has remained stable while hyperintensity has decreased. **b**, Longitudinal MRI of the pontine lesion of S053. Axial T2-weighted (top row) and axial post-contrast T1-weighted (bottom row) MRI images, focused on the pontine lesion at various timepoints (see column labels). At DX, the pons is enlarged (arrows) with diffuse T2 hyperintensity, heterogeneous enhancement and partial effacement of the fourth ventricle (asterisks). Following radiation (Post RT, 2 months before IT), there is further expansion of the pons and increased T2 signal abnormality (arrows), with persistent enhancement. From Pre IT to 2 months Post IT, the pontine lesion is smaller (arrows). However, by 4 months Post IT, the lesion is larger, with a new region of enhancement (arrowheads). At 6 and 8 months Post IT, the lesion size, T2 signal abnormality and enhancement are reduced.

A total of 18 patients received sufficient CAR T cell infusions to be evaluable for neuroimaging assessment. The best individual neuroimaging responses on MRI were 1 partial response (PR; 6%), 15 stable disease (SD; 83%) and 2 PD (11%). Of patients evaluable for disease response who had progressed prior to treatment, 88.9% recorded a best neuroimaging response of SD or PR after initiating protocol therapy. While S021 is not classified as an objective radiographic response, MRI reveals a sustained and ongoing decrease in pontine T2 hyperintensity (Fig. 3a). While S053 experienced a PR correlating with improvement in clinical symptoms, this patient ultimately had tumor progression at metastatic sites of disease 60 days later (Fig. 3b).

### CAR T cell detection and cytokine analysis

**Extended Data Fig. 2:**
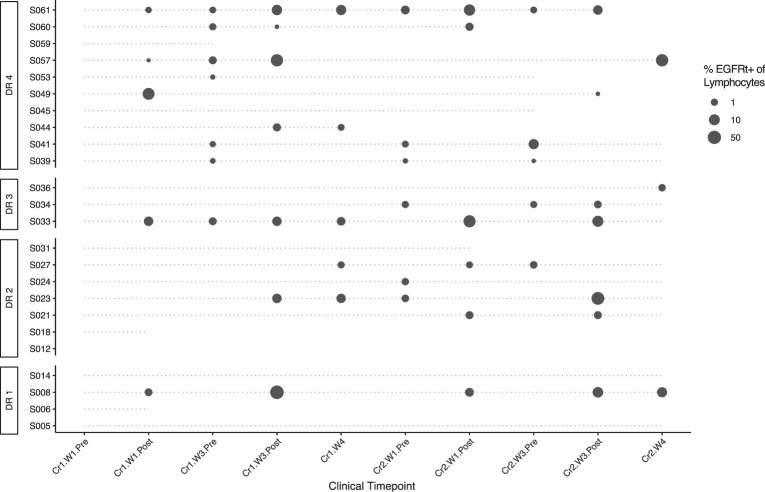
Intracranial Detection of CAR T Cells in CSF. Detection of B7-H3 CAR T cells in CSF pre- and post- infusion during the DLT period consists of 10 scheduled collections: Cr1.W1.Pre, Cr1.W1.Post, Cr1.W3.Pre, Cr1.W3.Post, Cr1.W4, Cr2.W1.Pre, Cr2.W1.Post, Cr2.W3.Pre, Cr2.W3.Post, Cr2.W4, which are defined as (where Cr: course; W: week; Pre: pre-infusion, and Post: post-infusion). The x-axis shows the sequence of these collections. Samples with lymphocytes count below the limit of quantitation (LOQ) were excluded from analysis. Shaded circles represent samples where B7-H3 CAR T cells were detected, defined by lymphocyte/EGFRt+% levels above the limit of detection (LOD) and a minimal count (>1) of detected EGFRt+ cells. The size of the circles denotes the percentage of EGFRt+ lymphocytes (Lymph/EGFRt+%) detected in the sample. Notably, S014 had no sample collected due to the placement of the shunt, S018 only received one dose, and S045 missed all post-infusion collection.

**Extended Data Fig. 3:**
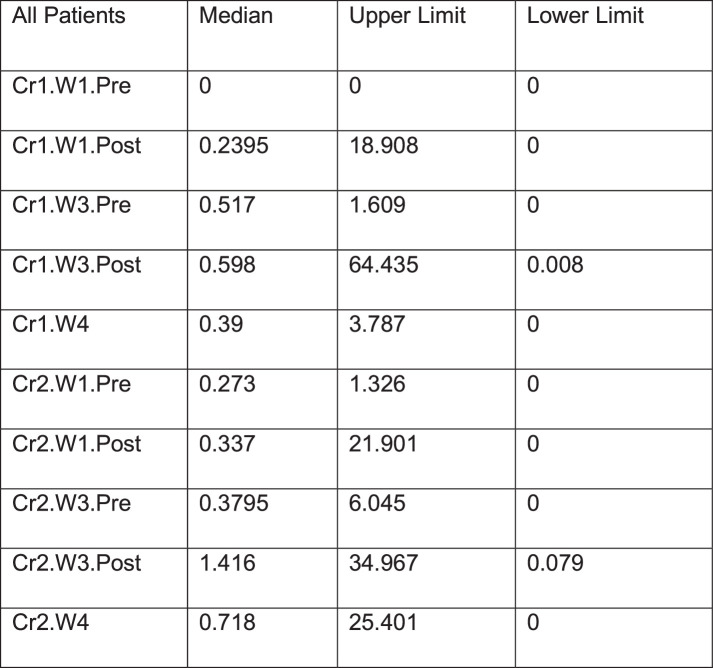
CSF CAR T cell detection Table. Levels of CAR detection by EGFRt+ across all patients and across all dose regimens (DR1, DR2, DR3 and DR4) during the DLT period. Gating Strategy: Live> Singlets> Lymphocyte> CD3+> EGFRt+.

A secondary objective was to assess B7-H3 CAR T cell distribution in the CSF. To complete this, we collected serial CSF samples from the CNS catheter before and after CAR T cell infusions, then performed flow cytometry to detect the truncated epidermal growth factor receptor (EGFRt) transduction tag. B7-H3 CAR T cells were detected via their EGFRt transduction tag in 38.1% (40 of 105) of CSF biospecimens in courses 1 and 2 (Extended Data Figs. 2 and 3 and Supplementary Table) with 13 of 18 (72%) evaluable patients having detectable CAR T cells in at least one timepoint. The median peak CAR T cell detection was at the course (Cr) 2 week (W) 3 post-infusion timepoint. Overall, Cr2 timepoints showed higher median detection than matched Cr1 timepoints, except Cr2.W3.Pre. Of the 96 peripheral blood samples collected, only 2 had detectable vector (S021: Cr2.W4: 264.4 copies μg^−1^ by qPCR; S039: Cr2.W1.Post 205.5 copies μg^−1^ by qPCR). Overall, this supports the finding that while ICV delivered B7-H3 CAR T cells are consistently detected in CSF after infusion, systemic circulation was rare, transient and at low levels.

**Fig. 4:**
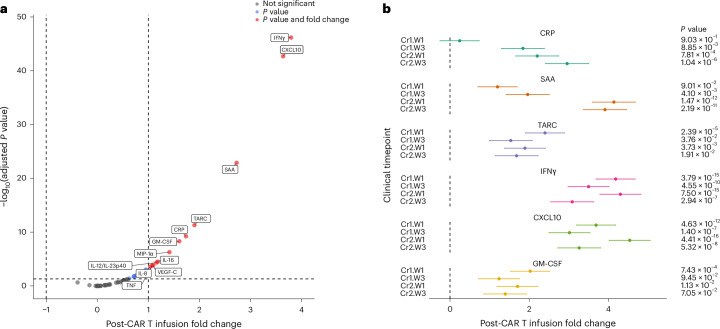
Chemokine and cytokine concentrations in CSF during the DLT period. **a**, A volcano plot of all 53 cytokines tested. The labels indicate cytokines showing at least a twofold change and a false discovery rate (FDR)-adjusted *P* < 0.05 across all four course–week combinations. **b**, A forest plot for six cytokines that show distinctive pre- and post-infusion patterns with subsequent infusions: those trending with cumulative infusions (CRP, SAA and TARC) and those consistently upregulated or downregulated after each infusion (IFNγ, CXCL10 (also known as IP-10) and GM-CSF). Data are presented as the model estimate of the post–pre-change on a log_2_ scale ± s.e.m. For differential analyses, only samples with matched pre- and post-infusion pairs at each Cr and W combination from the same patient were included. For the analysis presented in the volcano plot, the linear mixed model combined data from Cr1.W1 (*n* = 12), Cr1.W3 (*n* = 11), Cr2.W1 (*n* = 11) and Cr2.W3 (*n* = 10), resulting in 44 pre-infusion and 44 post-infusion measurements for each cytokine meeting quality filters. Study participants were included as random intercepts, and pre- and post-infusion status was included as a fixed effect. Analytes were measured in duplicate, samples with signal coefficients of variation greater than 25% were excluded and concentrations below the LLOD were considered undetectable (0 pg ml^−1^). Data were log_2_ transformed for analysis, and fold changes are presented on the log_2_ scale.

**Extended Data Fig. 4:**
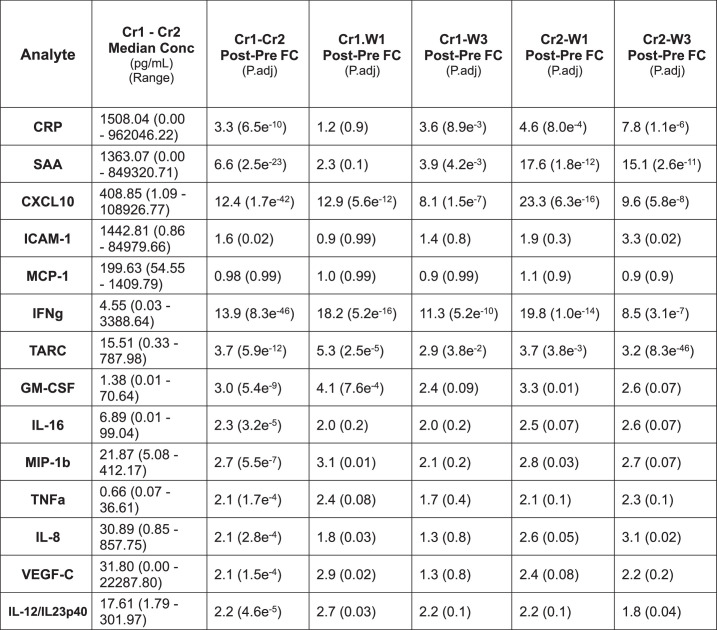
CSF cytokine data. Absolute concentrations and pre-infusion versus post-infusion changes for cytokines exhibiting at least a two-fold change and a false discovery rate (FDR)-adjusted p-value < 0.05 in a linear mixed model. The model combined data from Cr1.W1 (n = 12), Cr1.W3 (n = 11), Cr2.W1 (n = 11), and Cr2.W3 (n = 10), resulting in n = 44 pre-infusion and n = 44 post-infusion measurements for each cytokine that met quality criteria. Subjects were included as random intercepts, and pre/post-infusion status was included as a fixed effect. Analytes were measured in duplicate; those with coefficients of variation (CV) greater than 25% were excluded. Concentrations were log_2_-transformed for analysis, and fold changes are presented on a linear scale. Subsequent columns show fold changes and adjusted p-values for the separate and combined infusion time points.

An exploratory objective of this study was to assess biomarkers indicative of CAR T cell activity. To achieve this, 53 cytokines associated with T cell function and immune microenvironment interactions were measured using a Meso Scale Discovery (MSD) assay. A total of 105 CSF samples during courses 1 and 2 from 18 evaluable patients were analyzed using matched pre- and post-infusion biospecimens from the following timepoints: Cr1.W1 (*n* = 12), Cr1.W3 (*n* = 11), Cr2.W1 (*n* = 11) and Cr2.W3 (*n* = 10) (Extended Data Fig. 4). Following the initial CAR T infusion (Cr1.W1), significant elevations were observed in the levels of CXC motif chemokine ligand 10 (CXCL10, also known as IFNγ-induced protein 10 (IP-10)), granulocyte–macrophage colony-stimulating factor (GM-CSF), IFNγ, and thymus and activation-regulated chemokine (TARC) (Fig. 4a,b). Interestingly, the inflammatory markers CRP and serum amyloid A (SAA) did not significantly increase after the first infusion but showed notable elevations following subsequent infusions (Fig. 4a,b). While GM-CSF and TARC showed their most pronounced increases after the first infusion, CXCL10 and IFNγ levels consistently increased after each infusion. In aggregate, these findings support locoregional CAR T cell activation and cytotoxic activity.

## Discussion

We present results from the completed first-in-human phase 1 trial BrainChild-03 Arm C delivering repetitive ICV doses of B7-H3 CAR T cells to children and young adults with DIPG. While other groups have generated B7-H3 CAR T cells^13,14,17–22^ and our team had delivered them systemically for patients with solid tumors^23^, here we present, to our knowledge, the first completed ICV B7-H3 CAR T cell trial. We show tolerability of repetitive ICV dosing up to 10 × 10^7^ cells, cumulatively as high as 3 × 10^9^ CAR T cells in one patient.

Regarding tolerability, the single DLT occurred in a patient with progressive DIPG (unbiopsied) who received the lowest CAR T cell dose (1 × 10^7^ cells), then 1 week later developed intratumoral hemorrhage. While spontaneous tumor hemorrhage is a known natural risk^24^ and this was a solitary event, hemorrhage deserves attention on future trials as it also was seen in a patient receiving GD2 CAR T cells^9^. Notably, we did not find it necessary to monitor intracranial pressure for this specific product. Tumor-inflammation-associated neurotoxicity^25^ was described after trial initiation, so it was not captured prospectively, although a retrospective assessment of tumor-inflammation-associated neurotoxicity across all of our CNS CAR T cell trials is planned.

Regarding efficacy, ICV B7-H3 CAR T cells may improve survival, but this evaluation is inherently limited as it is a phase 1 study. One patient (S053) met radiographic criteria for PR. We cannot definitively conclude that response was solely due to CAR T cell activity as it is possible that the observed radiographic change could be a decrement of previous radiation-induced pseudoprogression unassociated with CAR T cell therapy. S021—a patient with a histone-mutant tumor who has received 81 doses and remains on protocol therapy—has had near resolution of T2 hyperintensity. However, the designation for complete response is challenging as the tumor often diffusely expands the pons and resolution of pontine size to normal may not mathematically characterize a response. Considering the historical consistency of survival in patients with DIPG, survival among newly diagnosed patients represents the best metric of clinical success. The median survival of 19.8 months for all treated patients is superior to the historical median survival of 11.2 months^1^ and the 9.4 month survival after initial CAR T cell infusion for patients enrolled after progression is superior to the <3 month historical progression to death^1^. However, historical values may underrepresent modern survival as supportive care measures have improved and re-irradiation has become standard^26^. Patients also may have benefitted from cytotoxic chemotherapy, re-irradiation or targeted medications after discontinuing protocol therapy. Notably, while our longest survivor still on protocol therapy (S021) has molecularly confirmed DMG, another long-term survivor (S008) has a high-grade glioma with an *IDH1* mutation that is associated with improved survival, as are mutations in *BRAF* as seen in S045. As decades of DIPG clinical trials before the discovery of the H3K27M alteration found no survival benefit, our results warrant further investigation. This is especially true as clinical trials for children with DIPG often exclude patients based on metastatic disease, locoregional extension outside of the pons or overall tumor size, none of which were exclusion criteria on this trial.

Tumor biopsy was not required for enrollment because B7-H3 is expressed on most DIPGs^13–16^ and because we did not want to exclude patients coming from underserved areas where, despite the relative safety^27,28^, biopsy was discouraged or not available. This is unlikely to limit our safety analysis but restricts our ability to correlate efficacy with the degree of B7-H3 expression. To assess other metrics of CAR T cell activity, we evaluated CSF cytokine levels and confirmed signals of intracranial inflammation following CAR T cells, although the small patient numbers preclude correlation to survival. It is clear that cytokines related to CAR T cell trafficking, such as CXCL10, which was also identified in our previous publications^6,11^, are elevated following infusion, although it is impossible to specify that this is directly from CAR T cell engagement with target antigen and that the concentration gradient peaks within the tumor. Notably, a recent study found a decrease in CXCL10 concentrations associated with timing of tumor progression^9^. A range of correlative studies including CSF mass spectrometry and CSF circulating tumor DNA will be completed in a search for biomarkers of failure, response and toxicity. Neuroimaging analysis following immunotherapy is challenging^29^ with an additional layer of complexity for DIPG as the pons itself can only decrease in size to a certain extent and T2 hyperintensity measurements are subject to imaging artifact and reporter bias. Therefore, advanced machine-learning volumetric and diffuse based imaging investigations are ongoing to address these limitations^30^.

Currently, several other leading centers including City of Hope, St. Jude, Stanford and Texas Children’s have developed clinical pediatric CNS CAR T cell programs, although this is the first report of a completed trial arm delivering ICV B7-H3 CAR T cells. A recent report by Stanford detailed the experience of delivering GD2 CAR T cells to children and young adults with DMG, with notable differences from our trial including their enrollment of only biopsy-proven DMG, their exclusion of patients with bulky thalamic or cerebellar disease, and their incorporation of lymphodepletion with intravenous dosing that was later amended to include repeated ICV dosing^9^. Their study found CRS to be dose limiting following intravenous GD2 CAR T cell dosing, but they did not find CRS complicating ICV infusions. Our trial used exclusively ICV dosing from inception, allowed enrollment of children receiving dexamethasone and did not incorporate lymphodepletion. Notably, their median age was 15 and included spinal DMG, while the median age was 6 years on our study and only patients with DIPG were treated. Their trial also allowed re-irradiation while on protocol therapy, which was not allowed here. While both studies had comparable survival for treated patients with DIPG (17.6 months for Stanford’s GD2 trial, 19.8 months on our B7-H3 trial) and, atypically, have patients surviving multiple years beyond their enrollment when the historical 2 year survival is 5% (ref. ^31^), it is critical to note that these are phase 1 clinical trials not fully powered to delineate efficacy. In aggregate, this work has laid a foundation for phase 2 studies, as well as clinical trials incorporating enhanced engineering strategies and combinatorial therapeutic regimens.

Ultimately, our clinical trial has shown that repeatedly dosed ICV B7-H3 CAR T cells are tolerable and feasible for children and young adults with DIPG. A planned multisite phase 2 trial will aim to fully define efficacy, while ongoing preclinical efforts aim to enhance CAR T cell migration and effector function, as well as identify synergistic combinatorial regimens, which we hope will improve the lives of children and young adults with DIPG.

## Methods

### CAR T cell product

Our B7-H3-specific CAR T cells (SCRI-CARB7H3(s)) and Good Manufacturing Practices were previously described^6^. Briefly, the second-generation, 4-1BB:zetaCAR is appended to a T2A ribosomal skip sequence followed by an EGFRt cell-surface tag^32^. A methotrexate-resistant human DHFR mutein (huDHFR^FS^; L22F, F31S) was appended to allow enrichment with methotrexate ex vivo^33^.

### Objectives

The study’s primary objectives were to assess the feasibility, safety and tolerability of ICV delivery of B7-H3 CAR T cells for children and young adults with DIPG and to define the maximally tolerated phase 2 dose regimen (RP2DR). Feasibility was primarily assessed by the ability to generate sufficient product to receive all planned doses in courses 1 and 2 from a single apheresis. Safety and tolerability were primarily assessed by history and physical exams, laboratory and radiographic evaluations, and Common Terminology Criteria for Adverse Events (CTCAE v5.0). A DLT was defined as an event that is possibly, probably or definitely attributable to CAR T cells and occurs from the initial CAR T cell infusion through 28 days following the final CAR T cell infusion. A DLT included all ≥ grade 3 CTCAE v5.0 toxicities except ≥ grade 3 toxicities known to be related to CAR T cells including the following: grade 3 CRS that decreased to ≤ grade 2 within 72 h; ≥grade 3 hypotension, fever and/or chills not controlled with medical intervention that decreased to ≤ grade 2 within 72 h; ≥ grade 3 activated PTT, fibrinogen and/or INR that were asymptomatic and resolved within 72 h; ≥ grade 3 hypoglycemia and/or electrolyte imbalance that was asymptomatic and resolved within 72 h; ≥ grade 3 nausea and/or vomiting that decreased to ≤ grade 2 within 7 days; and grade 3 neurologic symptoms that decreased to ≤ grade 2 within 21 days. DLTs included any toxicity lasting >14 days preventing the patient from receiving subsequent doses in course 1 or 2. Patients were considered DR escalation evaluable if evaluable for toxicity and counted in a dose-escalation cohort. Radiologic response criteria used the sum of the two longest two-dimensional perpendicular diameters to distinguish SD, PD (>25% increase), PR (>50% decrease) and complete response.

### Patients

Enrollment criteria for BrainChild-03 Arm C included age ≥1 and ≤26 years; DIPG (diagnosed radiographically or via histopathology confirming high-grade glioma or DMG) at any timepoint following completion of standard radiation; ability to tolerate apheresis; presence of a CNS catheter; life expectancy ≥8 weeks; Lansky or Karnofsky performance of ≥60; study-determined washout from previous therapies; adequate organ function including an absolute lymphocyte count ≥100 cells µl^−1^, absolute neutrophil count ≥500 cells µl^−1^, hemoglobin ≥9 g dl^−1^, platelets ≥100,000 µl^−1^, creatinine ≤ the upper limit of normal, total bilirubin <3× the upper limit of normal or conjugated bilirubin <2 mg dl^−1^, an oxygen saturation ≥90%, adequate neurologic function defined as stable deficits for ≥1 week, ≤2 antiepileptic agents required and no encephalopathy; negative virology for HIV and hepatitis B and C; and use of contraception in patients of child-bearing age. Exclusion criteria included dexamethasone >2.5 mg m^−2^ d^−1^; severe cardiac dysfunction; primary immunodeficiency or bone marrow failure; impending CNS herniation; presence of > grade 3 dysphagia; another active malignancy; severe, active infection; active receipt of any anticancer therapy; or pregnancy and/or breastfeeding. Patients and/or their guardians provided written informed consent in accordance with local regulatory review.

### Study design and treatment

BrainChild-03 began accrual on 22 November 2019. Clinical data through 13 November 2024 are included. The first reported patient was enrolled in August 2020, and the last reported patient was enrolled in April 2023. This study was conducted in accordance with FDA and International Conference on Harmonisation Guidelines for Good Clinical Practice, the Declaration of Helsinki and applicable institutional review board requirements, including study protocol approval by the Seattle Children’s Institutional Review Board. BrainChild-03 Arm C patients underwent leukopheresis, CAR T cell manufacture and infusions through their CNS catheter. DRs other than DR1 (in which all doses were DL1) used an intra-patient DL (Fig. 1). Dose escalation and de-escalation decisions were made using a modified 3 + 3 design. The MTDR was defined as the highest DR with at least 6 DLT-evaluable study participants whose cumulative DLT rate during courses 1 and 2 was below 34%. The study was monitored by a data monitoring committee. The BrainChild-03 protocol and list of amendments are available in Supplementary Information.

Requirements to receive CAR T cell infusions included a CNS catheter^5^, ≥5 days from surgery, evidence of disease, not breastfeeding and/or pregnant, meeting study-defined washout periods from bridging therapy, adequate study-defined organ function, no encephalopathy or uncontrolled seizure activity, compliance with prescribed antiepileptic drug(s), no evidence of active severe infection and no previous DLT. Beyond course 2, patients were eligible to receive additional infusions at the previous maximum tolerated DL, if the above criteria were met and sufficient CAR T cells were available. Response was assessed following course 2, and subsequent even-numbered courses, via MRI and CSF cytology. Following the initial DLT observation period, eligible patients could continue therapy (at least 1 dose every 28 days for an infinite number of courses). Tumor-directed therapy following discontinuation of protocol therapy was not collected as this was a phase 1 trial. Follow-up is ongoing, with patients monitored until death or for 15 years in long-term follow-up.

### Statistical analysis

Sample size was based on the phase 1 3 + 3 design. The MTDR was defined as the highest DR with at least six DLT-evaluable study participants and a cumulative DLT rate during courses 1 and 2 below 34%. Study participant demographics, clinical characteristics and CAR T manufacturing feasibility were summarized with descriptive statistics including frequencies and percentages for categorical data and medians with ranges for continuous variables. The lengths of intervals between diagnosis and enrollment, first CAR T infusion, and death or end of follow-up were described using ordinary median and range statistics. Kaplan–Meier analysis was used for evaluating survival time from the first CAR T infusion. Overall survival was defined as time from first CAR T infusion to death. Surviving study participants were censored at most recent follow-up. A supplemental intent-to-treat survival analysis starting from the time of enrollment and including the study participants who did not receive any CAR T product was also done. SAS 9.4 (SAS Institute) software was used for analyses of clinical and time-to-event variables.

### CSF analysis

#### CSF processing

**Extended Data Fig. 5:**
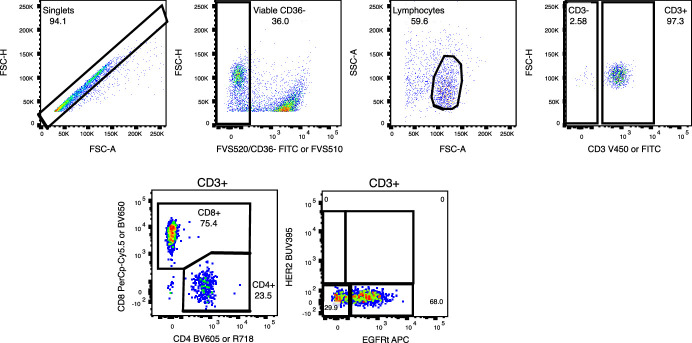
Representative flow gating strategy for CART cell detection. Gating strategy used for flow-based CAR detection in CSF. Selection of the singlet, viable [CD36-] lymphocyte population was performed prior to T cell gating and exam­ination of the EGFRt+ CART cells and CD4/CD8 expression.

Patient CSF samples were collected via lumbar puncture or ventricular catheter and kept at 4 °C until processing. The samples underwent serial centrifugation: first at 250 × *g* for 10 min to remove cells, followed by a final centrifugation at 10,000 × *g* for 10 min to remove any remaining debris. The cell-free supernatant was then aliquoted and cryopreserved at −80 °C. CAR T cell expression was quantified by detecting the EGFRt transduction tag using cetuximab custom conjugated to allophycocyanin (BD Biosciences). In addition, cells were stained with custom-biotinylated trastuzumab followed by streptavidin (BD Bioscience) for detection of a HER2 tag, which was not relevant to this trial. CAR T cells were identified as singlets, lymphocytes and viable cells and characterized by the phenotype CD3^+^/EGFRt^+^ HER2^−^ (with or without CD36^−^). The expression of CD4 and CD8 in both CAR^+^ and CAR^−^ populations was evaluated. BD LSRFortessa was used. Representative flow gating strategies are provided in Extended Data Fig. 5. Samples with lymphocyte counts under the limit of quantitation requirement for the assay were excluded from the report. The CAR T cell detection status was determined by a combination of at least one detectable EGFRt^+^ cell count in the sample and the level of CD3^+^/EGFRt^+^ cells as a percentage of lymphocytes in the sample to be above the pre-defined limit of detection (LOD) for the assay.

#### Electrochemiluminescence assays

Patient CSF samples were thawed and assessed for cytokines and chemokines using the V-PLEX Plus Human Biomarker 54-Plex Kit (MesoScale Diagnostics, catalog number K15248G), following the manufacturer’s instructions. All CSF samples were diluted according to the manufacturer’s recommendations, except for the analytes CXCL10 and MCP-1, which were diluted 50-fold for measurement. A precoated 96-well plate with capture antibody was used. The plate was blocked with MSD Blocker A for 1 h at room temperature. Then, the plate was washed, diluted standards and samples were added in duplicates to the respective wells and the plate was incubated overnight at 4 °C with shaking. Following the overnight incubation, a proprietary SULFO-TAG-conjugated detection antibody was added to the wells and the plate was incubated for 1–2 h at room temperature. The plate was washed again, developed using read buffer and measured using the MESO QuickPlex SQ 120 instrument. Data processing was conducted using MSD Discovery Workbench version 4.0.13 software after a final wash step. Standard curves were generated using a five-parameter logistic model. The concentration of each analyte was extrapolated from the standard curve. Values below the lowest LOD (LLOD) were considered undetectable. Statistical analyses for correlative cytokine data were conducted in R, using the lme4, emmeans and EnhancedVolcano packages^34,35^. Cytokine levels were measured in duplicate, and those with a coefficient of variation exceeding 25% were excluded. Differential expression between pre- and post-infusion timepoints was assessed using linear mixed-effects models, with study participants included as random intercepts and log2-transformed cytokine expression values as the response variable. A single model was fitted to include all cytokines and timepoints, and linear contrasts were calculated to evaluate pre- versus post-infusion differences at the specified timepoints.

### Peripheral blood CAR T cell detection

CAR T cell peripheral blood detection was assessed by qPCR quantification of the human 5-lipoxygenase-activating protein elongation factor-1 (FLAP-EF1; details provided in Supplementary Information). Genomic DNA was extracted from mononuclear cells. The in vivo persistence of CAR T cells was evaluated through batched analysis. A standard curve for the transcript copy number was generated by amplifying serial dilutions of the plasmid epHIV7. The number of transgene copies per nanogram of genomic DNA input was then determined. Measurements were taken from distinct samples.

### Reporting summary

Further information on research design is available in the Nature Portfolio Reporting Summary linked to this article.

## Online content

Any methods, additional references, Nature Portfolio reporting summaries, source data, extended data, supplementary information, acknowledgements, peer review information; details of author contributions and competing interests; and statements of data and code availability are available at 10.1038/s41591-024-03451-3.

## Supplementary information

Supplementary Information qPCR reagents, protocol for BrainChild-03 and history of amendments to BrainChild-03.

Reporting Summary

Supplementary Table CSF flow cytometry data. Flow cytometric analysis of longitudinal CSF samples collected during course 1 and course 2. The data show CD3^⁺^ T cells as a percentage of total lymphocytes and CD4^⁺^/CD8^⁺^ T cells as percentages of CD3^⁺^ T cells.

## Data Availability

All requests for raw and analyzed data and materials should be made to J.P.W. (jason.wendler@seattlechildrens.org). Requests will be promptly reviewed by the intellectual property office of Seattle Children’s Research Institute to verify whether the request is subject to any intellectual property or confidentiality obligations. Raw preclinical and clinical data are stored at Seattle Children’s with indefinite appropriate backup. Patient-related data not included in the paper were generated as part of a clinical trial and may be subject to patient confidentiality. Any data and materials that can be shared will be released via a Material Transfer Agreement.
