# Supplementary material for: Intracerebroventricular B7-H3-targeting CAR T cells for diffuse intrinsic pontine glioma: a phase 1 trial
Source: Nat Med. 2025 Jan 7;31(3):861–8. doi: 10.1038/s41591-024-03451-3 (PMC11922736; doi:10.1038/s41591-024-03451-3)
Supplement: Supplementary file 1 — qPCR reagents, protocol for BrainChild-03 and history of amendments to BrainChild-03. [file 41591_2024_3451_MOESM1_ESM.pdf]

nature medicine

Supplementary information https://doi.org/10.1038/s41591-024-03451-3

Intracerebroventricular B7-H3-targeting CAR T cells for diffuse intrinsic pontine glioma: a phase 1 trial

In the format provided by the authors and unedited

qPCR Reagents

Reagent Id	Oligo Type	Direction	Panel	Sequence	Fluorophore	Internal Quencher	Quencher	Company
oCS0007	Primer	Forward	FLAP-EF1	GGACAGCAGAGATCCAGTTTGG	N/A	N/A	N/A	IDT
oCS0008	Primer	Reverse	FLAP-EF1	GCAGATCCTCGCGAAGCA	N/A	N/A	N/A	IDT
tCS0001	Probe	N/A	FLAP-EF1	AAACGCCTAACCCTAAG	FAM	N/A	MGBNFQ	Applied Biosystems

PHASE 1 STUDY OF B7-H3-SPECIFIC CAR T CELL LOCOREGIONAL IMMUNOTHERAPY FOR DIFFUSE INTRINSIC PONTINE GLIOMA, DIFFUSE MIDLINE GLIOMA, AND RECURRENT OR REFRACTORY PEDIATRIC CENTRAL NERVOUS SYSTEM TUMORS

Protocol Number: BrainChild-03

Protocol Version:9.1

Protocol Version Date: 18 December 2023

**Investigational Product Name:**	SCRI-CARB7H3(s)
**Indication:**	Recurrent or refractory pediatric central nervous system tumors, diffuse intrinsic pontine glioma, and diffuse midline glioma
**IND/EudraCT Number:**	19043
**Sponsor:**	Seattle Children's, Seattle Children's Therapeutics<br>1920 Terry Avenue<br>M/S CURE-10<br>Seattle, WA 98101
**Sponsor's Responsible Medical Officer:**	Colleen Annesley, MD<br>Medical Director, Seattle Children's Therapeutics<br>Seattle Children's<br>4800 Sand Point Way NE<br>Seattle, WA 98105

This study is to be performed in compliance with the protocol, Good Clinical Practices (GCP) and applicable regulatory requirements.

Confidentiality Statement: This confidential information about an investigational product is provided for the exclusive use of investigators of this product and is subject to recall at any time. Subject to the foregoing, this information may be disclosed only to those persons involved in the study who have a need to know, with the obligation not to further disseminate this information.

Seattle Children's® Therapeutics

Protocol Version History:

Protocol V1.0	20Aug2019
Protocol V2.0	04Mar2020
Protocol V3.0	28Sep2020
Protocol V4.0	05May2021
Protocol V4.1	08Jun2021
Protocol V5.1	18Aug2021
Protocol V6.0	27Jan2022
Protocol V7.0	19Aug2022
Protocol V8.1	10Feb2023
Protocol V9.0	10Oct2023
Protocol V9.1	18Dec2023

Seattle Children's Therapeutics

TABLE OF CONTENTS

LIST OF ACRONYMS, ABBREVIATIONS, AND DEFINITION OF TERMS 8
ABSTRACT 10
EXPERIMENTAL DESIGN SCHEMA 11
1 BACKGROUND/RATIONALE 12
1.1 CNS Tumors of Children and Young Adults 12
1.2 Role of Immunotherapy in CNS Tumors 13
1.3 Redirected Specificity of T Cells Engineered to Express Chimeric Antigen Receptors 13
1.4 Clinical Experience Using CAR T Cells 14
1.5 B7-H3 as a Target in CNS Tumors 15
1.6 B7-H3 CAR T Cells 16
1.7 Safety of B7-H3 as a Therapeutic Target 16
1.8 Construction of Second-Generation Self-Inactivating (SIN) Lentivirus Vectors that Co-express a B7-H3 CAR and EGFRt 17
1.9 Intracavitary and CNS Dosing of CAR T Cells 17
1.10 CNS Catheters 18
1.11 Intra-Subject Dose Escalation 18
1.12 T Cell Dosing Rationale 19
1.13 Rationale for Use of This Product in Pediatric Subjects 20
2 STUDY OBJECTIVES 21
2.1 Primary Objectives 21
2.2 Secondary Objectives 21
2.3 Exploratory Objectives 21
3 ENROLLMENT AND ELIGIBILITY 22
3.1 Enrollment 22
3.2 Eligibility 22
3.2.1 Inclusion criteria 22
3.2.2 Exclusion criteria 24
4 DRUG INFORMATION 26
4.1 T Cell Product 26
4.1.1 Possible side effects of intracranially delivered CAR T cell therapy 26
5 TREATMENT PROGRAM 27
5.1 Overview of Treatment Plan 27
5.1.1 Treatment Arm assignment 27
5.1.2 Dose Regimen assignment 27
5.1.3 Description of Dose Regimens 28
5.1.4 Arm A and B Dose Regimens 1 through 3 29
5.1.5 Arm C Dose Regimens 1 through 4 32
5.1.6 Treatment Plan Delays 35

5.2 Dose-Limiting Toxicity (DLT) 36
5.3 Apheresis for T Cell Isolation 37
5.3.1 Requirements for apheresis 37
5.4 Bridging Therapy 37
5.5 CAR T Cell Product Infusion 37
5.5.1 Requirements for initial CAR T cell infusion 37
5.5.2 Requirements for subsequent CAR T cell infusion and dose modification 40
5.6 Guidelines for Administering the CAR T Cell Product 40
5.7 Concomitant Medication and Supportive Care 41
5.7.1 Anti-seizure medication 41
5.7.2 Blood product support 41
5.7.3 Antimicrobial prophylaxis 42
5.7.4 Prohibited medications 42
6 MANAGEMENT OF TOXICITIES AND COMPLICATIONS 43
6.1 Symptoms Associated with Apheresis 43
6.2 Symptoms Associated with CAR T Cell Infusion 43
6.3 Management of Cytokine Release Syndrome (CRS) 44
6.4 Management of Non-CRS Toxicity Associated with Infused CAR T Cells into the CNS 45
6.5 Ablation of T Cells with Cetuximab 46
7 STUDY PROCEDURES AND ASSESSMENTS 49
7.1 Informed Consent/Assent 49
7.2 Demography 49
7.3 Medical History 49
7.4 Performance Status 49
7.5 Physical Examination, Vital Signs, Weight, Height 49
7.6 Pulse Oximetry 49
7.7 Neurologic Exam 49
7.8 Concomitant Medications 49
7.9 Adverse Events 50
7.10 Pregnancy Test 50
7.11 Hematology 50
7.12 Chemistry 50
7.13 Cytokine Release Syndrome Labs and Evaluation 50
7.14 Virology 50
7.15 Cerebrospinal Fluid (CSF) Sampling 51
7.16 Correlative Sciences 51
7.17 CNS Imaging 51
7.18 Disease Response 52
7.18.1 Disease response assessment during active treatment 52

7.18.2 Disease response assessment following active treatment 52
7.19 Post-Treatment Long-Term Follow-Up data collection 52
7.19.1 Post-treatment long-term follow-up data collection 52
7.19.2 Post-Treatment Long-Term Follow-up Research Testing 53
7.19.3 Autopsy 53
8 REMOVAL FROM PROTOCOL THERAPY & OFF STUDY CRITERIA 54
8.1 Removal from Protocol Therapy 54
8.2 Off-Study Criteria and Study Termination 54
9 STATISTICAL CONSIDERATIONS 56
9.1 Accrual and Study Duration 56
9.2 Primary Objectives 56
9.3 Secondary and Exploratory Objectives 57
9.4 Statistical Analysis 58
9.5 Safety and Tolerability 58
9.6 Survival Definitions 58
9.7 Evaluability for Dose Escalation 59
9.8 Evaluability for Disease Response 59
9.9 Cohort Size and Rules for Cohort Advancement 59
9.9.1 Arms and DLT observation periods 59
9.9.2 Dose Regimen (DR) escalation rules 60
9.9.3 Stopping rules 61
9.9.4 Definition and determination of MTDR and RP2DR 62
9.10 Expansion Cohort 62
9.11 Safety Monitoring 62
9.11.1 Weekly safety review 62
9.11.2 Data Safety Monitoring Board 63
10 DISEASE RESPONSE 64
10.1 Disease Response Criteria 64
10.1.1 Measurable disease 64
10.1.2 Evaluable disease 64
10.1.3 Selection of target and non-target lesions 64
10.1.4 Response criteria for target lesions 64
10.1.5 Response criteria for non-target lesions 65
10.1.6 Evaluation of best overall response 65
11 ADVERSE EVENTS AND SERIOUS ADVERSE EVENTS 67
11.1 Definition of Adverse Event (AE) 67
11.2 Definition of Serious Adverse Event (SAE) 68
11.3 Classification of an Adverse Event 68
11.3.1 Grading of adverse events 68
11.3.2 Relationship of adverse event to treatment 69

11.4 Expectedness, Pre-Existing Conditions, and Persistent Adverse Events 70
11.5 Cytokine Release Syndrome Grading 71
11.6 Neurologic Toxicity Grading 71
11.7 Serious Adverse Event Reporting 71
11.7.1 Study-specific SAE reporting 72
11.8 IND Safety Reporting.. 72
11.9 On-Target, Off-Tumor Toxicities... 73
11.10 Reporting of Pregnancy.. 73
11.11 Safety Reporting Contact Information 73
12 ADMINISTRATIVE, ETHICAL, AND REGULATORY CONSIDERATIONS..... 74
12.1 Good Clinical Practice.. 74
12.2 Institutional Review Boards (IRB) and Institutional Biosafety Committees (IBC) 74
12.3 Informed Consent/Assent and Other Informational Documents Provided to Study Participants........ 75
12.4 Data Handling and Record Keeping... 75
12.4.1 Case report forms and source documents 75
12.4.2 Data quality assurance 76
12.4.3 Record retention.. 76
12.5 Investigational Product Accountability 76
12.6 Protocol Deviations 76
12.7 Subject-Specific Biologic Materials.. 77
12.8 Investigator's Responsibilities.. 77
12.9 Publication Policy... 78
12.10 Financing and Insurance.. 78
13 REFERENCES 79
14 APPENDICES 85
APPENDIX 1A – SPONSOR SIGNATURE PAGE.. 86
APPENDIX 1B – PRINCIPAL INVESTIGATOR SIGNATURE PAGE 87
APPENDIX 2 – SCHEDULE OF PROCEDURES 88
APPENDIX 3 – PERFORMANCE STATUS SCALES.. 99
APPENDIX 4 – NEUROLOGIC TOXICITY GRADING SYSTEM 100
APPENDIX 5 –REFRACTORY AND RECURRENT DISEASE CATEGORIZATION 101
APPENDIX 6 – HIGHLY EFFECTIVE CONTRACEPTION 102

Study Committee

STUDY CHAIR

Rebecca Ronsley, MD
Pediatric Neuro-oncology
Seattle Children's Hospital
4800 Sand Point Way NE
Seattle, WA 98105
Phone: 206-987-2106
email: rebecca.ronsley@seattlechildrens.org

STUDY STATISTICIAN

Qian (Vicky) Wu, PhD
Assistant Member, Clinical Research Division
Fred Hutchinson Cancer Research Center
1100 Fairview Avenue N
Seattle, WA 98109
Phone: 206-667-3358
email: qwu@fredhutch.org

STUDY MEMBERS

Julie R. Park, MD
Department of Oncology
St. Jude Children's Research Hospital
262 Danny Thomas Place
Memphis, TN 38105
Phone: 901-596-4329
email: Julie.park@stjude.org

Samuel Browd, MD, PhD
Pediatric Neurosurgery
Seattle Children's Hospital
4800 Sand Point Way NE
Seattle, WA 98105
Phone: 206-987-2544
email: samuel.browd@seattlechildrens.org

Bonnie Cole, MD
Neuropathology
Seattle Children's Hospital
4800 Sand Point Way NE
Seattle, WA 98105
Phone: 206-987-2103
email: bonnie.cole2@seattlechildrens.org

Juliane Gust, MD, PhD
Pediatric Neurology
Seattle Children's Hospital
4800 Sand Point Way NE
Seattle, WA 98105
Phone: 206-987-2087
email: juliane.gust@seattlechildrens.org

Francisco Perez, MD, PhD
Neuroradiology
Seattle Children's Hospital
4800 Sand Point Way NE
Seattle, WA 98105
Phone: 206-987-2134
email: francisco.perez@seattlechildrens.org

Nicholas Vitanza, MD
Pediatric Neuro-oncology
Seattle Children's Hospital
4800 Sand Point Way NE
Seattle, WA 98105
Phone: 206-987-28730
email: nicholas.vitanza@seattlechildrens.org

LIST OF ACRONYMS, ABBREVIATIONS, AND DEFINITION OF TERMS

ACD	acid-citrate-dextrose
AE	adverse event
ALC	absolute lymphocyte count
ALL	acute lymphoblastic leukemia
ALT	alanine aminotransferase
ANC	absolute neutrophil count
BUN	blood urea nitrogen
CAR	chimeric antigen receptor
CFR	Code of Federal Regulations
CNS	central nervous system
CR	complete response
CRF	Case Report Form
CRS	cytokine release syndrome
CSF	cerebrospinal fluid
CSL	Correlative Sciences Laboratory
CTEP	Cancer Therapy Evaluation Program
DIPG	diffuse intrinsic pontine glioma
DL	dose level
DLT	dose-limiting toxicity
DMG	Diffuse Midline Glioma, H3 K27M-mutant
DR	Dose Regimen
DSMB	Data Safety Monitoring Board
FDA	Food and Drug Administration
GBM	Glioblastoma
GCP	Good Clinical Practice
GMP	Good Manufacturing Practice
HIPAA	Health Insurance Portability and Accountability Act
HIV	human immunodeficiency virus
IBC	Institutional Biosafety Committee
ICF	informed consent form
ICH	International Conference on Harmonisation of Technical Requirements for Registration of Pharmaceuticals for Human Use
IEC	Institutional Ethics Committee
IND	Investigational New Drug (application)
IR	incomplete response
IRB	Institutional Review Board

IV	Intravenous
MRI	magnetic resonance imaging
MTDR	Maximum tolerated Dose Regimen
NCI	National Cancer Institute
CTCAE	Common Terminology Criteria for Adverse Events
NRM	non-relapse mortality
OS	overall survival
PB	peripheral
PCR	polymerase chain reaction
PD	progressive disease
PFNS	preservative-free normal saline
PFS	progression-free survival
PI	principal investigator
PR	partial response
RCL	replication-competent lentivirus
RP2DR	recommended Phase 2 dose regimen
SAE	serious adverse event
SCRI	Seattle Children's Research Institute
TCPC	therapeutic cell production core
ULN	upper limit of normal
US	United States
VS	vital signs

Definition of Terms

Investigational Product is defined as, "A pharmaceutical form of an active ingredient or placebo being tested or used as a reference in a clinical trial, including a product with a marketing authorization when used or assembled (formulated or packaged) in a way different from the approved form, or when used for an unapproved indication, or when used to gain further information about an approved use" [from International Conference on Harmonization (ICH) of Technical Requirements for Registration of Pharmaceuticals for Human Use Harmonized Tripartite Guideline E6: Guideline for Good Clinical Practice].

The terms "Investigational Product" and "study drug" may be used interchangeably in the protocol.

Seattle Children's® Therapeutics

ABSTRACT

This is a Phase 1 study of central nervous system (CNS) locoregional adoptive therapy with autologous $CD4^+$ and $CD8^+$ T cells that are lentivirally transduced to express a B7-H3-specific chimeric antigen receptor (CAR) and EGFRt. CAR T cells are delivered via an indwelling catheter into the tumor cavity or ventricular system in children and young adults with diffuse intrinsic pontine glioma (DIPG), diffuse midline glioma (DMG), and recurrent or refractory CNS tumors. Depending on the location of the tumor and the existence of metastatic (including leptomeningeal) disease, subjects will have CAR T cells delivered into the tumor cavity or the ventricular system. The primary objectives of this protocol are to evaluate the feasibility, safety, and tolerability of CNS-delivered fractionated B7-H3 CAR T cell infusions employing intra-patient dose escalation and to define the Maximally Tolerated Dose (MTD) and Recommended Phase 2 Dose Regimen (RP2DR) of CNS-delivered fractionated B7-H3 CAR T cell infusions through a modified 3+3 design and an expansion cohort. The secondary objectives are to assess B7-H3 CAR T cell distribution within the cerebrospinal fluid (CSF) and the extent to which B7-H3 CAR T cells egress into the peripheral circulation, and disease response to B7-H3 CAR T cells. The exploratory objectives are to describe B7-H3 expression in tumor tissue and/or normal tissue if a tissue biopsy, tumor biopsy, or resection is available; to describe the presence of B7-H3 CAR T cells in tumor tissue and/or normal tissue if a tissue biopsy, tumor biopsy, or resection is clinically indicated post-treatment; and to analyze blood, CSF, and tumor tissue for biomarkers of anti-tumor B7-H3 CAR T cell expression, safety, and activity.

EXPERIMENTAL DESIGN SCHEMA

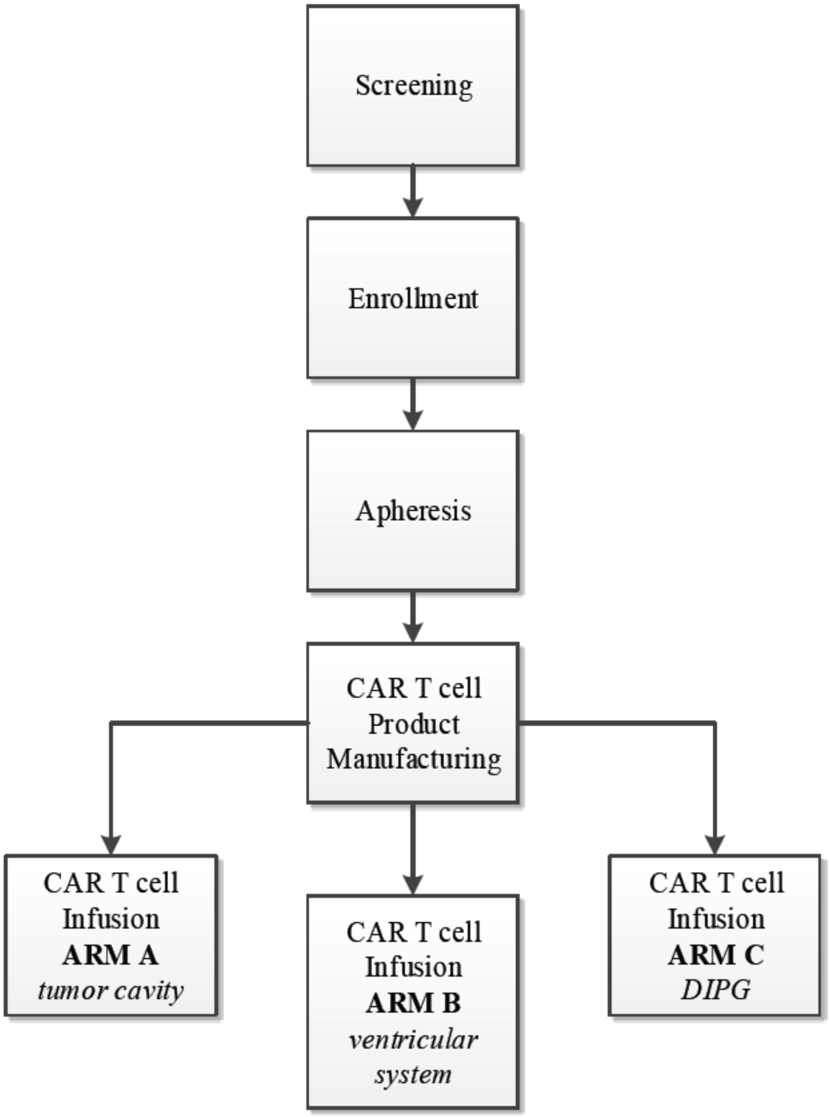

[redacted] [redacted]

[redacted] [redacted]

[redacted]

[redacted]

[redacted]

[redacted]

[redacted]

[redacted] [redacted]

[redacted]

[redacted]

[redacted] [redacted]

[redacted]

[redacted]

[redacted]

[redacted]

[redacted] [redacted]

[redacted]

[redacted]

[redacted]

[redacted]

[redacted] [redacted]

[redacted]

[redacted]

[redacted]

[redacted] [redacted]

[redacted]

[redacted] [redacted]

[redacted]

[redacted]

[redacted]

[redacted]

[redacted]

[redacted]

[redacted]

[redacted]

[redacted] [redacted]

[redacted]

[redacted] [redacted]

[redacted]

[redacted]

[redacted]

[redacted]

[redacted]

[redacted]

[redacted]

[redacted]

[redacted] [redacted]

[redacted]

[redacted]

[redacted]

2 STUDY OBJECTIVES

2.1 Primary Objectives

- To assess the feasibility of CNS locoregional adoptive therapy with autologous $CD4^+$ and $CD8^+$ T cells lentivirally transduced to express a B7-H3-specific CAR EGFRt and a methotrexate resistant human dihydrofolate reductase mutein, delivered by an indwelling catheter into the tumor cavity or ventricular system in children and young adults with DIPG, DMG, or recurrent/refractory CNS tumors.
- To assess the safety of CNS locoregional adoptive therapy with autologous $CD4^+$ and $CD8^+$ T cells lentivirally transduced to express a B7-H3-specific CAR, EGFRt and a methotrexate resistant human dihydrofolate reductase mutein, delivered by an indwelling catheter into the tumor cavity or ventricular system in children and young adults with DIPG, DMG, or recurrent/refractory CNS tumors.
- To establish the tolerability of a fractionated CNS-delivered B7-H3 CAR T cell infusion schedule employing intra-subject dose escalation in children and young adults with DIPG, DMG, or recurrent/refractory CNS tumors.
- To define the maximally tolerated dose (MTD) and recommended Phase 2 dose regimen (RP2DR) of CNS-delivered fractionated B7-H3 CAR T cell infusions.

2.2 Secondary Objectives

- To assess B7-H3 CAR T cell distribution within the cerebrospinal fluid (CSF) and the extent to which B7-H3 CAR T cells egress into the peripheral circulation.
- To assess disease response to B7-H3 CAR T cell locoregional therapy in children and young adults with DIPG, DMG, or recurrent/refractory CNS tumors.

2.3 Exploratory Objectives

- To evaluate for presence of B7-H3 CAR T cells in tumor tissue and/or normal tissue if a tissue biopsy, tumor biopsy, or resection is clinically indicated post-treatment.
- To evaluate B7-H3 expression in tumor tissue and/or normal tissue if a tissue biopsy, tumor biopsy, or resection is available.
- To analyze blood, CSF, and tumor tissue for biomarkers of anti-tumor B7-H3 CAR T cell expression, safety, and activity.

3 ENROLLMENT AND ELIGIBILITY

3.1 Enrollment

Upon consent for participation in a Seattle Children's Therapeutics immunotherapy clinical trial, subject demographic information is entered into the Immunotherapy Registration Portal and a unique Registration ID is appointed. Subjects are then transferred to the study-specific database where they are assigned a unique Study ID. Screening and eligibility information will be entered into the study-specific database where it will be reviewed by the Study Chair or their designee who will subsequently approve/deny subject enrollment to the study (consult the sponsor and/or case report form (CRF) Completion Guidelines for instructions related to the study-specific databases).

3.2 Eligibility

3.2.1 Inclusion criteria

1. Subjects must be age ≥ 1 and ≤ 26 years (except for the first 3 subjects, who will be enrolled in Arm A or Arm B and must be age ≥ 15 and ≤ 26 years) (this requirement has been met as of 8/27/2020).
2. Subject disease classified as one of the following:
   a. DIPG at any timepoint following completion of standard radiotherapy
   b. DMG at any timepoint following completion of standard radiotherapy
   c. Evidence of refractory or recurrent CNS disease for which there is no standard therapy, defined by either of the following (further categorization by disease type is listed in Appendix 5 –Refractory and Recurrent Disease Categorization):
      i. Recurrent disease (e.g., new site(s) of measurable or evaluable disease by radiographic imaging or histologic confirmation following completion of standard of care first-line therapy for which curative salvage therapy is not available or amenable), OR
      ii. Refractory disease (e.g., measurable or evaluable disease that persists following completion of standard of care first-line therapy for which curative salvage therapy is not available or amenable)
3. Able to tolerate apheresis or already has an apheresis product available for use in manufacturing
4. CNS reservoir catheter, such as an Ommaya or Rickham catheter, present in the proper location for CNS-directed therapy delivered as specified for BrainChild-03
5. Life expectancy ≥ 8 weeks
6. Lansky or Karnofsky score ≥ 60. Subjects who are unable to walk because of paralysis, but who are up in a wheelchair, will be considered ambulatory for purposes of assessing performance status

7. If subject does not have a previously obtained apheresis product that is acceptable and available for manufacturing of CAR T cells, subject must discontinue all anticancer agents and radiotherapy and, in the opinion of the investigator, have fully recovered from significant acute toxic effects of the following:
    a. *Cytotoxic chemotherapy/biologic therapy:* All cytotoxic chemotherapy/biologic therapy must be discontinued ≥ 7 days prior to enrollment.
    b. *Antibody Therapy:* The last dose of anti-tumor antibody therapy (including check point inhibitor) must be at least 3 half-lives or 30 days, whichever is shorter, from the time of enrollment. Bevacizumab will be considered biologic therapy.
    c. *Cellular Therapy:* must be at least 30 days from most recent cell infusion prior to enrollment
    d. *Steroid use:* All systemically administered (i.e. Subcutaneous, Intramuscular, PO or IV) corticosteroid therapy (unless physiologic replacement dosing) must be stable or decreasing for ≥ 1 week prior to enrollment, with a maximum dexamethasone dose of 2.5 mg/m^2/day ongoing at enrollment. Corticosteroid physiologic replacement therapy for management of pituitary/adrenal axis insufficiency and/or topical administration (e.g. inhaled or dermatologic) is allowed.
8. Adequate hematologic values, all of the following in Table 3-1 must be true:

Table 3-1 Screening Hematologic Values

Analyte	Value
Absolute lymphocyte count (ALC) [1]	≥ 100 cells/uL
Absolute neutrophil count (ANC)	≥ 500 cells/uL
Hemoglobin [2]	≥ 9 g/dL
Platelets [2]	≥100,000/µL

[1]Value not required to be met if subject has previously obtained apheresis product acceptable and available for manufacturing of CAR T cells

[2]Subjects receiving blood product transfusion(s) are acceptable as long as they are not determined to be transfusion refractory

9. Adequate renal function, as indicated by serum creatinine ≤ the upper limit of normal (ULN), per Table 3-2:

Table 3-2 Serum Creatinine ULN Based on Age and Sex

Serum Creatinine (mg/dL) ULN		
**Age**	**Male**	**Female**
1 to < 2 yrs	0.6	0.6
2 to < 6 yrs	0.8	0.8
6 to <10 yrs	1	1
10 to < 13 yrs	1.2	1.2
13 to < 16 yrs	1.5	1.4

≥ 16 yrs	1.7	1.4

10. Adequate hepatic function as indicated by either of the following:
    a. Total bilirubin < 3 times ULN for age, OR
    b. Conjugated bilirubin < 2 mg/dL
11. Adequate respiratory function as indicated by BOTH of the following:
    a. Oxygen saturation ≥ 90% on room air without supplemental oxygen or mechanical ventilation, AND
    b. No dyspnea at rest
12. Adequate neurologic function as indicated by all of the following:
    a. Signs and symptoms of neurologic deficit must be stable for ≥ 1 week prior to enrollment, AND
    b. ≤ two anti-epileptic agents are required to control seizure activity, AND
    c. No clinically evident encephalopathy present
13. Virology negative within 3 months prior to enrollment, to include all of the following:
    a. HIV antigen & antibody, AND
    b. Hepatitis B surface antigen, AND
    c. Hepatitis C antibody OR if antibody positive, Hepatitis C polymerase chain reaction (PCR) is negative
14. Subjects of childbearing/fathering potential must agree to use highly effective contraception (Appendix 6 – Highly Effective Contraception) from the time of enrollment through 12 months following the last T cell infusion

3.2.2 Exclusion criteria

1. Presence of ≥ Grade 3 cardiac dysfunction or symptomatic arrhythmia requiring intervention
2. Presence of primary immunodeficiency/bone marrow failure syndrome
3. Presence of clinical and/or radiographic evidence of impending herniation
4. For Arm C subjects only: Presence of > Grade 3 dysphagia
5. Presence of active malignancy other than the CNS tumor under study
6. Presence of active severe infection, defined as either of the following:
    a. Positive blood culture within 48 hours of enrollment, OR
    b. Fever > 38.2ºC AND clinical signs of infection within 48 hours of enrollment
7. Pregnant or breastfeeding
8. Subject and/or authorized legal representative unwilling to provide consent/assent for study participation, including participation in the 15-year follow-up period, which is required if CAR T cell therapy is administered

9. Presence of any condition that, in the opinion of the investigator, would prohibit the subject from undergoing treatment under this protocol

4 DRUG INFORMATION

4.1 T Cell Product

Investigational product for BrainChild-03 is composed of autologous CD4+ and CD8+ T cells that express an B7-H3-specific CAR and EGFRt. The B7-H3-specific CAR consists of a scFv binding domain derived from the B7-H3-binding monoclonal antibody huBRCA84D. The CAR's signaling domains include 4-1BB and CD3ζ chain.

T cell product manufacturing will be conducted in the SCRI Therapeutic Cell Production Core (TCPC) GMP facility under approved Investigational New Drug (IND) processes. Cryopreserved cells will be stored in vapor phase in a controlled access LN2 (liquid nitrogen) freezer until released for clinical use. On the day of T cell product infusion, cryopreserved unit(s) will be thawed, aseptically washed, and formulated in a sterile, single-use syringe as specified in standard thaw and wash procedures. Formulated CAR T cell product must be administered prior to the expiration date/time specified in standard thaw and wash procedures and recorded on the syringe label.

4.1.1 Possible side effects of intracranially delivered CAR T cell therapy

Please see the SCRI-CARB7H3(s) Investigator's Brochure for safety information.

5 TREATMENT PROGRAM

5.1 Overview of Treatment Plan

5.1.1 Treatment Arm assignment

Subjects will be enrolled into one of three Treatment Arms according to disease pathology, disease location, and subsequent CNS-directed CAR T cell delivery route.

Arm A will include subjects whose CAR T cells will be delivered into the tumor cavity.

Arm B will include subjects whose CAR T cells will be delivered into the ventricular system.

Arm C will include subjects with DIPG whose CAR T cells will be delivered into the ventricular system.

Within each Arm, subjects will be enrolled into a defined Dose Regimen (DR). Subjects will receive a different combination of Dose Levels (DL) depending on the DR into which they are enrolled.

The specific DLs within each DR are:

Table 5-1 CAR T Cell Dose Levels

Dose Level (DL)	Dose
DL 1 (test dose)	1 x 10^7 cells
DL 2	2.5 x 10^7 cells
DL 3	5 x 10^7 cells
DL 4	10 x 10^7 cells

During the time from apheresis to infusion of CAR T cells, subjects may return to the care of their primary physician and may receive additional cancer-directed therapy (see 5.4 Bridging Therapy). The site team will be notified when the subject's CAR T cell product is released and available so that the CAR T cell infusion may be scheduled.

CAR T cell therapy will be administered on Study Weeks 1 through 3 (Arms A and B) or Weeks 1 and 3 (Arm C) of each 4-week Course. Dosing will start with a test dose (DL 1) at Week 1. Arm A and B, DR 1 will establish the safety of the test dose plus a single DL escalation. Arm C DR 1 will establish the safety of the intermittent administration of the test dose without DL escalation.

5.1.2 Dose Regimen assignment

Detailed rules for DR assignment are described in Section 9.9.1 Arms and DLT observation periods.

If the quantity of the initial manufactured CAR T cell product is less than the specified DL, but is otherwise releasable by TCPC, it may be administered as long as the subject meets criteria to

undergo initial T cell infusion (Section 5.5.1 Requirements for initial CAR T cell infusion) and the occurrence is clearly documented in the subject files (see Section 5.6 Guidelines for Administering the CAR T Cell Product). However, in the absence of a DLT during Courses 1 and 2, such subjects will not be evaluable for DR escalation per Section 9.9.2 Dose Regimen (DR) escalation rules.

If a subject has a releasable CAR T cell product but treatment is suspended (during the mandated period between participants, or while the data are under review by the Data Safety Monitoring Board [DSMB] or Food and Drug Administration [FDA]) and the subject is not medically stable enough to wait until treatment is reinitiated, the subject may receive their CAR T cell product according to the schedule and specific CAR T cell DLs of the maximum DR already deemed tolerable.

Subjects who are removed/withdraw from the protocol prior to receiving CAR T cell product will be replaced to meet statistical guidelines for evaluation of toxicity and feasibility.

5.1.3 Description of Dose Regimens

Dose regimens (DR) will be the same for Arms A and B using an intra-patient dose escalation schema with weekly dosing during 3 of every 4 weeks. Arm C will use every other week dosing.

5.1.4 Arm A and B Dose Regimens 1 through 3

Figure 5-1 Arm A and B Dose Regimen 1

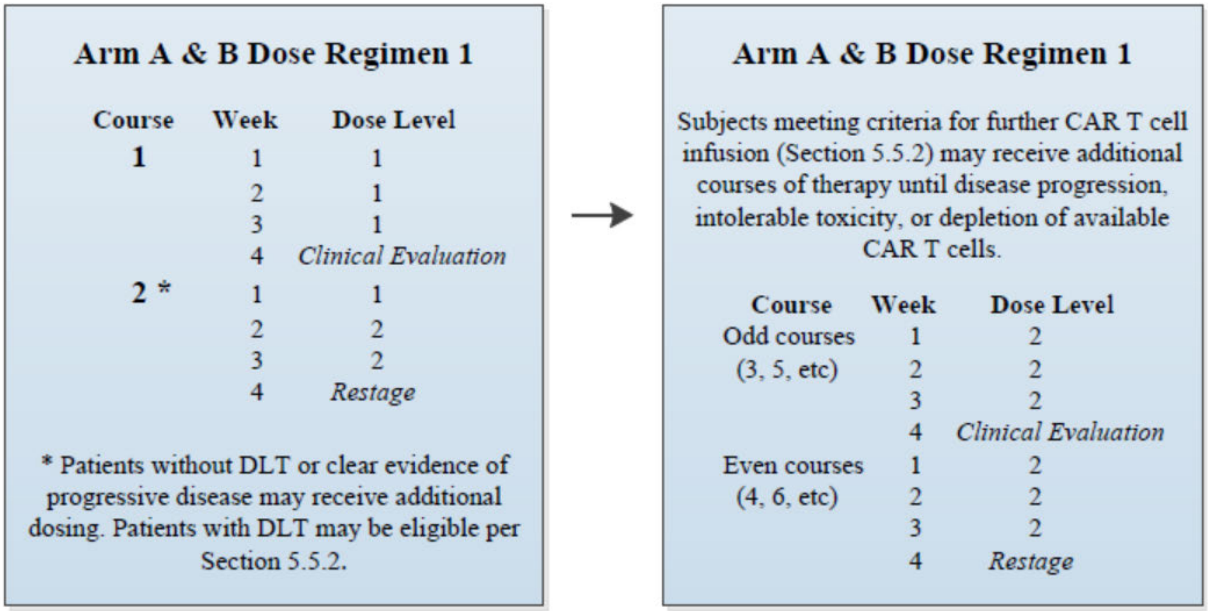

Subjects enrolled in Arm A and B DR 1 will receive Course 1 consisting of DL 1 on Weeks 1, 2, and 3 followed by a rest during Week 4, with clinical evaluations.

Course 2 will commence for subjects without DLT and who meet criteria for subsequent CAR T cell infusions (Section 5.5.2 Requirements for subsequent CAR T cell infusion and dose modification). Subjects will receive DL 1 on Week 1, DL 2 on Weeks 2 and 3, followed by a rest during Week 4 with disease evaluation, including radiographic restaging.

Following Course 2, subjects meeting criteria for further CAR T cell infusion (Section 5.5.2) may receive additional Courses of therapy until subject meets criteria for removal from protocol therapy, intolerable toxicity, or depletion of available manufactured CAR T cells. The DLs for Courses 3 and beyond will be no higher than DL 2 or the dose which no DLTs were experienced by the subject during Courses 1 and 2. A lower DL may be administered based on availability of CAR T cells, see Section 5.1.2 Dose Regimen assignment. Treatment will be administered on Weeks 1, 2, and 3 followed by a rest week every fourth week. Disease restaging evaluations will occur as indicated in the schedule of procedures.

Figure 5-2 Arm A and B Dose Regimen 2

Arm A & B Dose Regimen 2

Course	Week	Dose Level
**1**	1	1
	2	2
	3	2
	4	*Clinical Evaluation*
**2 ***	1	2
	2	3
	3	3
	4	*Restage*

* Patients without DLT or clear evidence of progressive disease may receive additional dosing. Patients with DLT may be eligible per Section 5.5.2.

→

Arm A & B Dose Regimen 2

Subjects meeting criteria for further CAR T cell infusion (Section 5.5.2) may receive additional courses of therapy until disease progression, intolerable toxicity, or depletion of available CAR T cells.

Course	Week	Dose Level
Odd courses	1	3
(3, 5, etc)	2	3
	3	3
	4	*Clinical Evaluation*
Even courses	1	3
(4, 6, etc)	2	3
	3	3
	4	*Restage*

Subjects enrolled in Arm A and B DR 2 will receive Course 1 consisting of DL 1 on Week 1, DL 2 on Weeks 2 and 3 followed by a rest during Week 4, with clinical evaluations.

Course 2 will commence for subjects meeting criteria for subsequent CAR T cell infusion (Section 5.5.2). Subjects will receive DL 2 on Week 1, DL 3 on Weeks 2 and 3, followed by a rest during Week 4 with disease evaluation, including radiographic restaging.

Following Course 2, subjects meeting criteria for further CAR T cell infusion (Section 5.5.2) may receive additional Courses of therapy until subject meets criteria for removal from protocol therapy, intolerable toxicity, or depletion of available manufactured CAR T cells. The DLs for Courses 3 and beyond will be no higher than DL 3 or the dose at which no DLTs were experienced by the subject during Courses 1 and 2. A lower DL may be administered based on availability of CAR T cells, see Section 5.1.2 Dose Regimen assignment. Treatment will be administered on Weeks 1, 2, and 3 followed by a rest week every fourth week. Disease restaging evaluations will occur as indicated in the schedule of procedures.

Figure 5-3 Arm A and B Dose Regimen 3

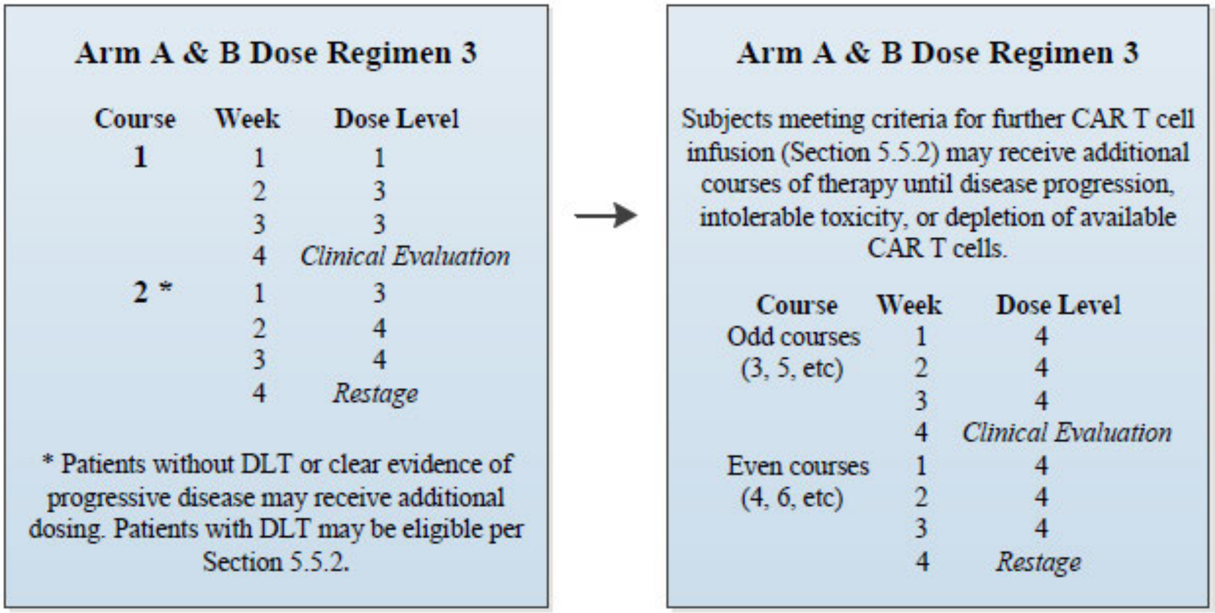

Subjects enrolled in Arm A and B DR 3 will receive Course 1 consisting of DL 1 on Week 1, DL 3 on Weeks 2 and 3 followed by a rest during Week 4, with clinical evaluations.

Course 2 will commence for subjects meeting criteria for subsequent CAR T cell infusion (Section 5.5.2). Subjects will receive DL 3 on Week 1, DL 4 on Weeks 2 and 3, followed by a rest during Week 4 with disease evaluation, including radiographic restaging.

Following Course 2, subjects meeting criteria for further CAR T cell infusion (Section 5.5.2) may receive additional Courses of therapy until subject meets criteria for removal from protocol therapy, intolerable toxicity, or depletion of available manufactured CAR T cells. The DLs for Courses 3 and beyond will be no higher than DL 4 or the dose at which no DLTs were experienced by the subject during Courses 1 and 2. A lower DL may be administered based on availability of CAR T cells, see Section 5.1.2 Dose Regimen assignment. Treatment will be administered on Weeks 1, 2, and 3 followed by a rest week every fourth week. Disease restaging evaluations will occur as indicated in the schedule of procedures.

5.1.5 Arm C Dose Regimens 1 through 4

Figure 5-4 Arm C Dose Regimen 1

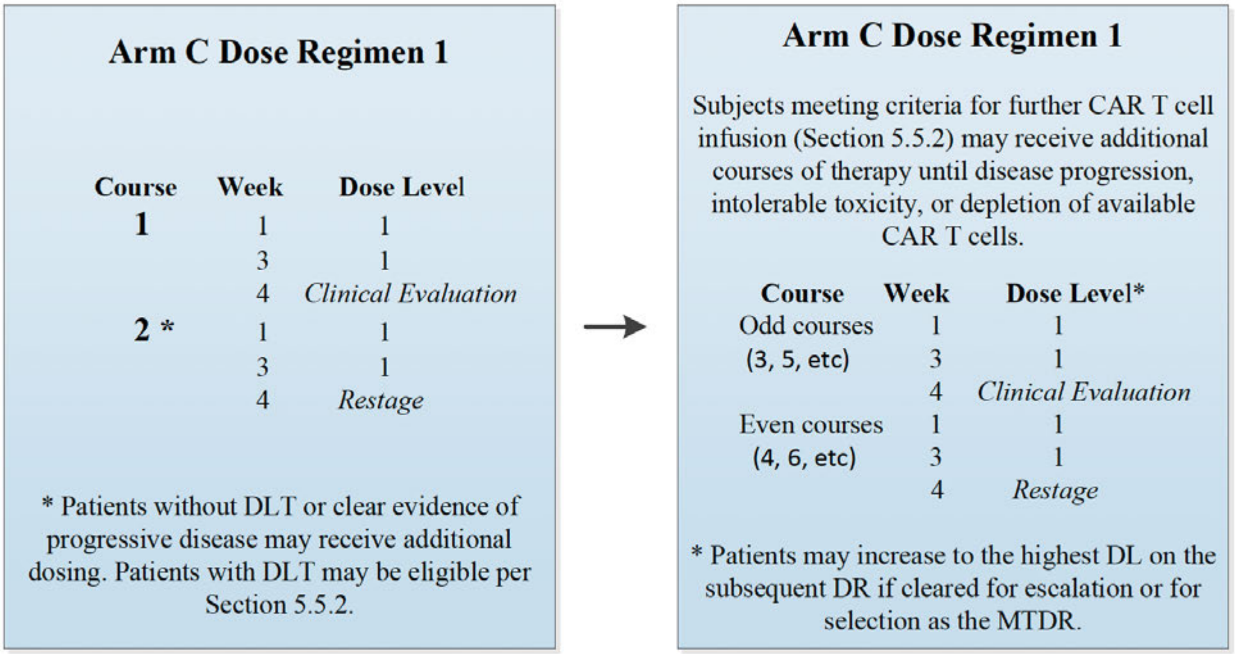

Subjects enrolled in Arm C DR1 will receive Course 1 consisting of DL 1 on Weeks 1 and 3 followed by a rest during Week 4, with clinical evaluations.

Course 2 will commence for subjects without DLT and who meet criteria for subsequent CAR T cell infusions (Section 5.5.2). Subjects will receive DL 1 on Week 1 and DL 1 on Week 3, followed by a rest during Week 4 with disease evaluation, including radiographic restaging.

Following Course 2, subjects meeting criteria for further CAR T cell infusion (Section 5.5.2) may receive additional Courses of therapy until subject meets criteria for removal from protocol therapy, intolerable toxicity, or depletion of available manufactured CAR T cells. The DLs for Courses 3 and beyond will be no higher than DL 1 unless a subsequent DR has been cleared for escalation or for selection as the MTDR. In that case, the subject may increase to the highest DL on the subsequent DR. Treatment will be administered on Weeks 1 and 3 followed by a rest week every fourth week. Disease restaging evaluations will occur as indicated in the schedule of procedures.

Seattle Children's® Therapeutics

Figure 5-5 Arm C Dose Regimen 2

Arm C Dose Regimen 2

Course	Week	Dose Level
**1**	1	1
	3	1
	4	*Clinical Evaluation*
**2 ***	1	2
	3	2
	4	*Restage*

* Patients without DLT or clear evidence of progressive disease may receive additional dosing. Patients with DLT may be eligible per Section 5.5.2.

→

Arm C Dose Regimen 2

Subjects meeting criteria for further CAR T cell infusion (Section 5.5.2) may receive additional courses of therapy until disease progression, intolerable toxicity, or depletion of available CAR T cells.

Course	Week	Dose Level*
Odd courses	1	2
(3, 5, etc)	3	2
	4	*Clinical Evaluation*
Even courses	1	2
(4, 6, etc)	3	2
	4	*Restage*

* Patients may increase to the highest DL on the subsequent DR if cleared for escalation or for selection as the MTDR.

Subjects enrolled in Arm C DR 2 will receive Course 1 consisting of DL 1 on Weeks 1 and 3 followed by a rest during Week 4, with clinical evaluations.

Course 2 will commence for subjects without DLT and who meet criteria for subsequent CAR T cell infusions (Section 5.5.2). Subjects will receive DL 2 on Weeks 1 and 3, followed by a rest during Week 4 with disease evaluation, including radiographic restaging.

Following Course 2, subjects meeting criteria for further CAR T cell infusion (Section 5.5.2) may receive additional Courses of therapy until subject meets criteria for removal from protocol therapy, intolerable toxicity, or depletion of available manufactured CAR T cells. The DLs for Courses 3 and beyond will be no higher than DL2 or the dose at which no DLTs were experienced by the subject during Courses 1 and 2 unless the subject did not experience a DLT and a subsequent DR has been cleared for escalation or for selection as the MTDR. If that occurs, then the DL may increase to the highest DL on the cleared DR. A lower DL may be administered based on availability of CAR T cells, see Section 5.1.2 Dose Regimen assignment.

Treatment will be administered on Weeks 1 and 3 followed by a rest week every fourth week. Disease restaging evaluations will occur as indicated in the schedule of procedures.

Figure 5-6 Arm C Dose Regimen 3

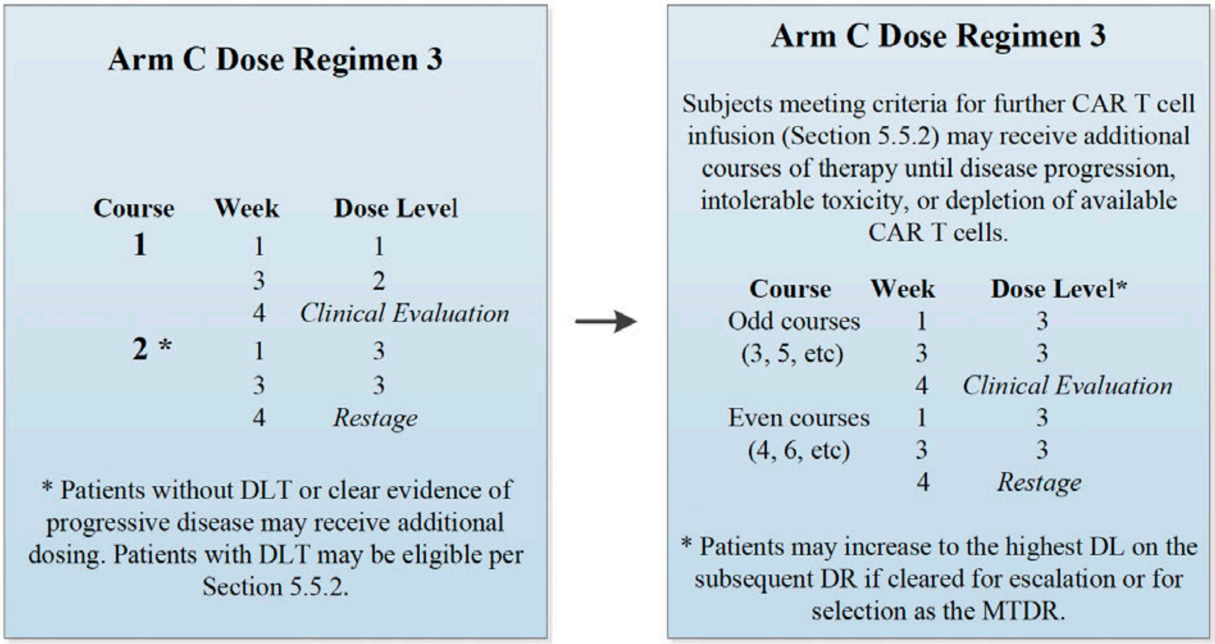

Subjects enrolled in Arm C DR 3 will receive Course 1 consisting of DL 1 on Week 1 and DL 2 on Week 3 followed by a rest during Week 4, with clinical evaluations.

Course 2 will commence for subjects meeting criteria for subsequent CAR T cell infusion (Section 5.5.2). Subjects will receive DL 3 on Weeks 1 and 3, followed by a rest during Week 4 with disease evaluation, including radiographic restaging.

Following Course 2, subjects meeting criteria for further CAR T cell infusion (Section 5.5.2) may receive additional Courses of therapy until subject meets criteria for removal from protocol therapy, intolerable toxicity, or depletion of available manufactured CAR T cells. The DLs for Courses 3 and beyond will be no higher than DL 3 or the dose at which no DLTs were experienced by the subject during Courses 1 and 2 unless a subsequent DR has been cleared for escalation or for selection as the MTDR. If that occurs, then the DL may increase to the highest DL on the cleared DR. A lower DL may be administered based on availability of CAR T cells, see Section 5.1.2 Dose Regimen assignment. Treatment will be administered on Weeks 1 and 3 followed by a rest week every fourth week. Disease restaging evaluations will occur as indicated in the schedule of procedures.

Figure 5-7 Arm C Dose Regimen 4

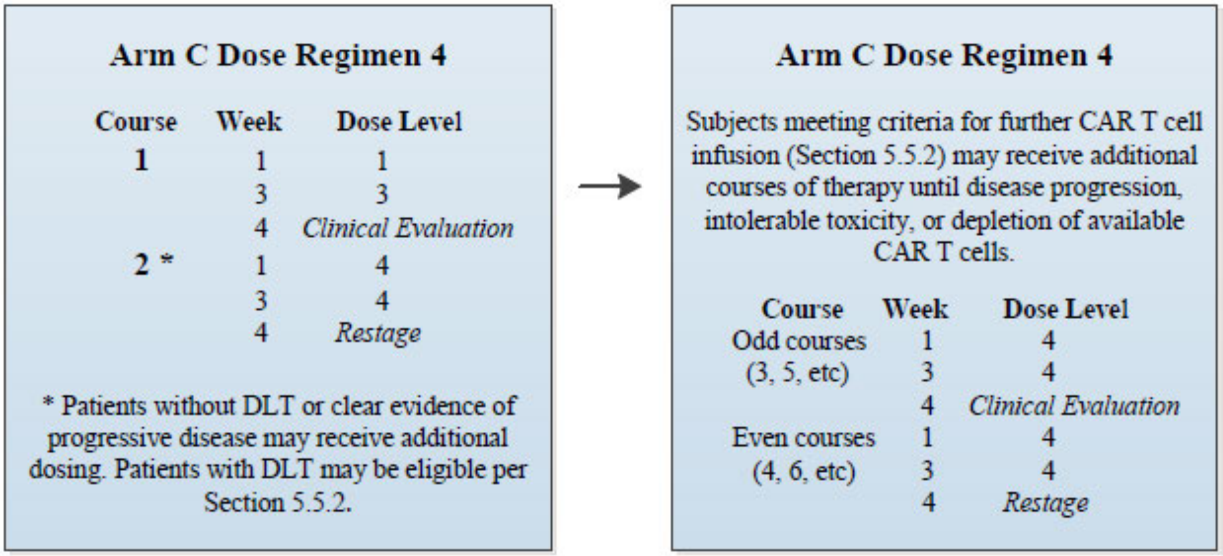

Subjects enrolled in Arm C DR 4 will receive Course 1 consisting of DL 1 on Week 1 and DL 3 on Week 3 followed by a rest during Week 4, with clinical evaluations.

Course 2 will commence for subjects meeting criteria for subsequent CAR T cell infusion (Section 5.5.2). Subjects will receive DL 4 on Weeks 1 and 3, followed by a rest during Week 4 with disease evaluation, including radiographic restaging.

Following Course 2, subjects meeting criteria for further CAR T cell infusion (Section 5.5.2) may receive additional Courses of therapy until subject meets criteria for removal from protocol therapy, intolerable toxicity, or depletion of available manufactured CAR T cells. The DLs for Courses 3 and beyond will be no higher than DL 4 or the dose at which no DLTs were experienced by the subject during Courses 1 and 2. A lower DL may be administered based on availability of CAR T cells, see Section 5.1.2 Dose Regimen assignment.

Treatment will be administered on Weeks 1 and 3 followed by a rest week every fourth week. Disease restaging evaluations will occur as indicated in the schedule of procedures.

5.1.6 Treatment Plan Delays

During each Course, the time interval between each CAR T cell infusion will be according to the Schedule of Procedures and based upon the date of the most recent CAR T cell infusion. The end date of each Course will be the date of completion of Week 4 evaluations per the Schedule of Procedures. The start date of each Course will be the date that the Week 1 CAR T cell infusion required evaluations are performed.

Within Course 1 and 2, if a subject does not meet the criteria to receive a CAR T cell infusion (Section 5.5.2) on the planned infusion day of a Course regardless of attribution to the CAR T cells, the infusion may be delayed no more than 3 weeks from the planned infusion date. If delayed greater than 3 weeks from the planned infusion date, the subject will be removed from

protocol therapy and will not receive further CAR T cell treatment. See Section 8.1 Removal from Protocol Therapy.

Between Course 2 and Course 3, delays due to family/subject preference, investigator discretion, or due to subject recovery from a surgical procedure (e.g., tumor biopsy) are allowed. Following Course 3, only delays due to subject recovery from a surgical procedure (e.g., tumor biopsy) or due to travel disruptions are allowed. During the delay subjects must continue to be followed for concomitant medications (Section 7.8) and adverse events (Section 7.9). Criteria for removal from protocol therapy in Sections 5.5.2 and 8.1 will override family/subject/investigator preference delays as noted above.

Beyond Course 2, subjects must receive at least the Week 1 infusion of any Course but may skip the other scheduled infusions. If a subject does not receive an infusion within 3 weeks of the Week 1 infusion, the next infusion will be considered a new Course. If infusions are delayed, the subsequent pre- and post-visit evaluations/procedures (see Appendix 2 – Schedule of Procedures) will be timed based upon the next administered CAR T cell infusion. If an infusion is not given, the pre- and post-visit procedures for that infusion are not required. If delay to subsequent infusion is greater than 8 weeks from prior infusion, disease response assessment must be obtained prior to dosing.

5.2 Dose-Limiting Toxicity (DLT)

A DLT is an event, defined below, which, in the opinion of the investigator, is possibly, probably, or definitely attributable to the CAR T cell product and which occurs from the time of initial CAR T cell infusion through 28 days following the final CAR T cell infusion. The Maximum Tolerated Dose Regimen (MTDR) will be established by the occurrence of DLTs which occur from the time of initial CAR T cell infusion through 7 days following the final CAR T cell infusion received during Course 2. See Section 9.9.4 Definition and determination of MTDR and RP2DR, for information on establishing MTDR.

The definition of a DLT includes all ≥ Grade 3 Common Terminology for AEs (CTCAE) v5 toxicities **except** ≥ Grade 3 toxicities that are known to be related to CAR T cells [45], listed below.

Exceptions to ≥ Grade 3 DLTs:

- Grade 3 CRS that decreases to ≤ Grade 2 within 72 hours of onset
- ≥ Grade 3 hypotension, fever, chills not controlled with medical intervention that decrease to ≤ Grade 2 within 72 hours
- ≥ Grade 3 activated PTT, fibrinogen, and/or INR that are asymptomatic and resolve within 72 hours
- ≥ Grade 3 hypoglycemia and/or electrolyte imbalance that are asymptomatic and resolve within 72 hours
- ≥ Grade 3 nausea and/or vomiting that decrease to ≤ Grade 2 within 7 days

- Arm A and B only: Grade 3 neurological adverse event that decreases to ≤ Grade 2 within 7 days
- Arm C only: Grade 3 neurological adverse event that decreases to ≤ Grade 2 within 21 days (treatment with dexamethasone and/or bevacizumab is allowed)

The definition of a DLT also includes any related toxicity lasting > 14 days which prohibits the subject from meeting criteria for subsequent CAR T cell infusion.

5.3 Apheresis for T Cell Isolation

Subjects who do not already have an apheresis product available for use in manufacturing will undergo apheresis to obtain T cells from which individualized CAR T cell therapy will be manufactured. Apheresis may take place in either an outpatient or inpatient setting and will be performed per institutional standard operating procedure. After meeting requirements for apheresis, subjects will undergo apheresis with a target total lymphocyte collection of 1×10^9.

In instances of manufacturing failure or insufficient availability of starting material subjects may undergo a repeat apheresis procedure if the Principal Investigator feels it may yield a successful product. Subjects who have already initiated CAR T cell infusions and are eligible for ongoing CAR T cell therapy but no longer have available CAR T cell product and who do not have sufficient starting material for additional manufacture may undergo repeat apheresis. All subjects undergoing apheresis or repeat apheresis must meet requirements for apheresis, noted in Section 5.3.1.

5.3.1 Requirements for apheresis

Subjects with active, severe, infection may not undergo apheresis. For the purpose of this trial, active, severe infection is defined as:

- Positive blood culture within 48 hours of blood draw, OR
- Fever > 38.2°C AND clinical signs of infection within 48 hours of blood draw

5.4 Bridging Therapy

During the period between apheresis and CAR T cell infusion, subjects may return to the care of their primary physician. Additional therapy aimed at controlling disease burden to allow the subject to meet criteria for CAR T cell infusion may be given. Refer to Section 5.5.1 Requirements for initial CAR T cell infusion and Table 5-2 for Pre-T Cell Therapy Wash-Out Requirements.

5.5 CAR T Cell Product Infusion

5.5.1 Requirements for initial CAR T cell infusion

The following criteria must be met prior to administration of initial CAR T cell product:

- First 3 subjects (this requirement has been met as of 8/27/2020):

- Age ≥ 15 and ≤ 26 years
- Only treated on Arm A or Arm B

- Subjects subsequent to the first 3 treated:
  - Age ≥ 1 and ≤ 26 years
  - May be enrolled on any Arm (Note: See Section 9.9.1 Arms and DLT observation periods: Subjects may be enrolled on Arm C once 3 subjects have been treated on Arm A and/or Arm B, and at least 1 subject on Arm A or B completes Course 2 without experiencing a DLT. This requirement has been met as of 04/29/20.)
- Subject has a CNS reservoir catheter in place
- Subject is ≥ 5 days from CNS surgery, including catheter placement
- Subject has confirmed evidence of persistent, evaluable disease
- Subject is not breastfeeding
- Female subjects of childbearing potential only: subject has a negative pregnancy test within 2 days prior to initial CAR T cell infusion
- Regarding bridging therapy:
  - If the subject has achieved defined partial or complete remission with bridging therapy, the Study Chair or designee will recommend to the subject's treating oncologist that the bridging therapy be continued. The subject may be re-evaluated and would be eligible to receive CAR T cell treatment upon meeting requirements for CAR T cell infusion.
  - The following treatments must be discontinued for the specified duration (washout period) prior to infusion of the CAR T cell product, and subject must have recovered from acute therapy associated toxicities.

Table 5-2 Pre-T Cell Therapy Washout Requirements

Treatment	Wash-out Period<br>(prior to CAR T cell product infusion)
Radiation therapy	≥ 6 weeks
Bevacizumab	≥ 28 days
Cytotoxic chemotherapy	≥ 21 days
Biologic agents	≥ 7 days
Antibody therapy<br>(eg, murine, chimeric or humanized monoclonal antibodies)<br>T cell growth factors<br>(eg, IL-2, IL-7 or IL-15)<br>Interferons<br>Vaccines	≥ 3 half-life or 30 days<br>(whichever is shorter)
Cellular therapy	≥ 30 days since last infusion
Any investigational agent	≥ 3 half-life or 30 days<br>(whichever is shorter)

- If the subject is receiving corticosteroids for symptomatic relief from their CNS disease, steroid dosing must be stable or decreasing for ≥ 1 week with a maximum dexamethasone dose of 2.5 mg/m^2/day on the day of the infusion. Corticosteroid physiologic replacement therapy for management of pituitary/adrenal axis insufficiency and/or topical administration (e.g., inhaled or dermatologic) is allowed.
- Subject must have adequate organ function as indicated by:
    - Renal: Serum creatinine ≤ ULN per Table 3-2 Screening Serum Creatinine Values
    - Hepatic: Total bilirubin < 3 times ULN for age OR conjugated bilirubin < 2 mg/dL
    - Respiratory: Oxygen saturation ≥ 90% on room air without supplemental oxygen or mechanical ventilation, and no dyspnea at rest
    - Hematologic: Platelets ≥ 100,000/µL (transfusion(s) allowed to meet criteria)
- No evidence of clinically significant progressive encephalopathy or uncontrolled seizure activity.
- Subject is taking an anti-seizure medication.
- No evidence of active severe infection, defined as: 1) positive blood culture within 48 hours of CAR T cell infusion; or 2) fever > 38.2°C AND clinical evidence of infection within 48 hours of CAR T cell infusion

5.5.2 Requirements for subsequent CAR T cell infusion and dose modification

The following criteria must be met prior to administration of subsequent CAR T cell product:

- No DLT (Section 5.2) has occurred following infusion of CAR T cell (See Section 8 Removal from Protocol Therapy & Off Study Criteria).
- Cryopreserved CAR T cells are available and released for infusion
- Subject must have adequate organ function as indicated by:
    - Renal: Serum creatinine ≤ ULN per Table 3-2
    - Hepatic: Total bilirubin < 3 times ULN for age OR conjugated bilirubin < 2 mg/dL
    - Respiratory: Oxygen saturation ≥ 90% on room air without supplemental oxygen or mechanical ventilation, and no dyspnea at rest
    - Hematologic: Platelets ≥ 100,000/µL (transfusion(s) allowed to meet criteria)
- No evidence of clinically significant progressive encephalopathy or uncontrolled seizure activity
- Subject is taking an anti-seizure medication
- No evidence of active severe infection, defined as: 1) positive blood culture within 48 hours of CAR T cell infusion; or 2) fever > 38.2°C AND clinical evidence of infection within 48 hours of CAR T cell infusion
- Subject has a CNS reservoir catheter in place
- Subject is not breastfeeding
- Female subjects of childbearing potential only: subject has a negative pregnancy test within 2 days prior to Week 1 CAR T cell infusion of each Course

5.6 Guidelines for Administering the CAR T Cell Product

The administered dose of CAR T cell therapy may be within ±10% of the protocol prescribed dose. Administering a dose of CAR T cell therapy varying by greater than ±10% of the protocol prescribed dose is not permitted without prior Sponsor approval. All dosing deviances should be communicated to the Sponsor and the occurrence clearly documented in the subject files. Please refer to Section 5.1 Overview of Treatment Plan for full details of the study dosing requirement.

Subjects may undergo CAR T cell infusion in the outpatient or inpatient setting. When clinically prudent, subjects may be admitted to the hospital following the CAR T cell infusion for observation and monitoring if they exhibit minor symptoms which, if to worsen, could place the subject at risk of an adverse medical outcome.

In addition to institutional guidelines for infusion of cellular products, subjects are to be administered CAR T cell product according to the following guidelines:

- Prior to CAR T cell product administration (in instances of documented allergy or sensitivity to any of the below pre-medications clinical discretion may be used for appropriate substitution):
    - Required: acetaminophen
        - Recommended dosing: 12.5 mg/kg (maximum dose 650 mg), PO
    - Optional: diphenhydramine
        - Recommended dosing: 0.5 mg/kg (maximum dose 50 mg), IV or PO
    - Optional: ondansetron
        - Recommended dosing: 0.15 mg/kg (maximum dose 8 mg), IV or PO
- Thawed, formulated CAR T cell product will be administered slowly through a catheter placed into the tumor cavity or ventricular system.
- Microaggregate filters and leukodepletion filters must not be used to infuse CAR T cell products.
- CAR T cell infusion must be followed by a preservative-free normal saline (PFNS) flush delivered slowly via a manual push technique to clear the administration line and catheter. The volume of PFNS is dependent on catheter size and will be determined at time of enrollment and then kept consistent for each CAR T cell infusion the subject receives.

5.7 Concomitant Medication and Supportive Care

5.7.1 Anti-seizure medication

Seizures may cause significant short and long-term impairment, however treatment-related seizures may be a preventable complication in subjects receiving localized therapy for intra-cranial tumors. It is required that seizure prophylaxis begin prior to the subject's initial CAR T cell infusion on study and continue until 1 month following the final CAR T cell infusion on study. Levetiracetam is recommended; however, an alternative anti-seizure medication may be given per investigator discretion.

5.7.2 Blood product support

All blood products will be transfused per institutional standards.

It is strongly recommended that platelet transfusions be given to maintain a platelet count ≥ 100,000/µL prior to initial, and between, CAR T cell infusions.

It is recommended that packed red blood cells (PRBCs) be given to maintain a hematocrit of ≥ 25% during the immediate post-CAR T cell infusion periods (to include the first 48 hours) and during periods of Grade 2-4 CRS, unless clinically the subject has a higher established transfusion threshold.

Cryoprecipitate should be given to maintain fibrinogen > 150 mg/dL.

5.7.3 Antimicrobial prophylaxis

Pneumocystis jiroveci pneumonia (PJP) prophylaxis should be given per institutional standards.

Meningeal dosing of antibiotics should be considered based on history, clinical presentation, and physical examination for subjects who develop fever > 38.2°C within 72 hours of CAR T cell infusion.

Infectious disease management should follow institutional standards but may be individualized as clinically indicated. Institutional standards for infectious diseases will guide addition of antimicrobial agents in cases of persistent or recurrent fever.

Additionally, if the CAR T cell product has a positive culture which is discovered after being infused into the subject, blood cultures will be drawn and appropriate antibiotic coverage will be initiated, as follows:

- For gram negative organisms: ceftazidime and gentamicin should be started until identification and susceptibility have been determined and antibiotic coverage can then be tailored to the specific bacteria. If the subject has a cephalosporin allergy, in infectious disease consult should be obtained to determine appropriate antibiotic coverage. Additionally, ciprofloxacin may be substituted for gentamicin if the subject has impaired renal function
- For fungal organisms: an infectious disease consult should be obtained to determine appropriate antibiotic coverage

If the subject's blood cultures remain negative for 5 days and the subject remains clinically well, antimicrobials may be discontinued.

5.7.4 Prohibited medications

The following agents are not allowed from the time of initial CAR T cell infusion through 28 days following the final CAR T cell infusion or time of removal from protocol therapy (Section 8.1) (whichever comes first):

- Anti-tumor directed chemotherapy
- Systemic immunosuppressive agents (other than corticosteroids) unless given to treat symptoms attributed to CAR T cells as noted in Section 6.3 Management of Cytokine Release Syndrome (CRS) and Section 6.4 Management of Non-CRS Toxicity Associated with Infused CAR T Cells into the CNS.
- Immunotherapy (other than the protocol-specified CAR T cell infusions)
- Other investigational agents, unless used to treat or prevent symptoms related to protocol CAR T cell infusions

6 MANAGEMENT OF TOXICITIES AND COMPLICATIONS

6.1 Symptoms Associated with Apheresis

Side effects that may occur during cell collection include nausea, vomiting, fainting or dizziness, seizures, skin rash, hives, flushing (redness and warmness of the skin, usually the face), blood loss, and infection. Tingling of the lips, muscle cramping, and, very rarely, changes in heart rhythm, may occur. These symptoms may be prevented or made milder by giving calcium supplements, either IV or PO during the apheresis procedure. Very rarely (< 1 in 1,000 procedures) clotting may occur in the apheresis machine or in the subject and is potentially life-threatening. To reduce the risk of clotting, acid-citrate-dextrose (ACD) and heparin may be given during the apheresis procedure. ACD may increase the risk of bleeding and may cause temporary tingling of the lips and limbs, muscle cramping, seizures, or changes in heart rhythm. Heparin may also increase the risk of bleeding. Transfusions of both PRBC and platelets may be required surrounding the procedure.

6.2 Symptoms Associated with CAR T Cell Infusion

Mild, transient symptoms have been observed while receiving or within 24 hours of receiving CAR T cell therapy including fevers, chills, rigors, headache, and, rarely, nausea, vomiting, hypotension, and pulmonary toxicity. Clinical changes may represent a recently reported distinct syndrome, tumor inflammation associated neurotoxicity (TIAN), that consists of immunotherapy-induced fever, headaches, and neurologic changes [84]. The management of these symptoms is outlined below.

Fever, chills and temperature elevations > 38.2°C may be managed with additional acetaminophen as clinically indicated, and/or 1 mg/kg meperidine (Demerol) IV for chills (maximum 50 mg). Additional methods such as cooling blankets may be employed for fevers resistant to these measures. All subjects who develop fever and/or chills should have a blood culture drawn and be admitted for IV antibiotics and supportive care.

Headache may be managed with additional acetaminophen as clinically indicated, and magnesium, diphenhydramine/metoclopramide, NS fluid, and/or narcotics may be considered.

Nausea and vomiting may be treated with additional ondansetron, and diphenhydramine/metoclopramide, scopolamine, lorazepam, and/or olanzapine may be considered.

Hypotension:

- Transient hypotension may initially be managed by intravenous fluid administration; however, subjects with persistent hypotension may require transfer to the intensive care unit (ICU) for definitive medical treatment.
- If significant hypotension occurs during the CAR T cell infusion, the infusion should be immediately suspended. Significant hypotension is defined as symptomatic and/or systolic blood pressure < 80 mmHg for age > 12 years, < 70 mm/hg for age < 12 years, or a 15% drop from baseline, whichever value is lower.

- Treatment for significant hypotension will follow institution standard practice.

Hypoxemia may be managed by standard clinical practice.

If the CAR T cell infusion is stopped before completion and the symptoms causing cessation return to baseline within 30 minutes, the CAR T cell infusion may be restarted at a slower rate. All product must be infused prior to the expiration time/date listed on the syringe label.

If the CAR T cell infusion is terminated due to acute toxicity occurring during the infusion, the residual CAR T cell product should be returned to the TCPC for analysis. Investigation of possible causes of observed symptoms should proceed and, if necessary, additional medical treatment should be instituted.

6.3 Management of Cytokine Release Syndrome (CRS)

All subjects for whom there is concern for CRS will be admitted to the hospital for observation. In subjects with adverse events (AEs) during *in vivo* expansion of CAR T cells, additional laboratory testing will be requested. If a subject is suspected of having CRS, additional samples may be sent for clinical cytokine analysis per treating physician discretion and additional Correlative Sciences specimens may be requested. Subjects may receive cytokine-directed therapy for symptom control including, but not limited to, tocilizumab, an IL-6 antagonist. The use of corticosteroids may be given to prevent a more serious toxicity. Table 6-1 provides guidelines for CRS management.

Table 6-1 Recommended Symptom Management for CRS

Symptom related to CRS	Suggested Intervention
Fever > 38.2°C	Acetaminophen (12.5 mg/kg) PO/IV up to every 4 hrs
Persistent fever ≥ 39 °C for 6 hrs that is unresponsive to acetaminophen	Tocilizumab (8-12 mg/kg) IV
Persistent fevers ≥ 39 °C after Tocilizumab	Dexamethasone 5-10 mg IV/PO up to every 6-12 hrs with continued fevers
Recurrence of symptoms 48 hrs after initial dose of Tocilizumab	Tocilizumab (8-12 mg/kg) IV
Hypotension	Fluid bolus, target hematocrit > 24%
Persistent/recurrent hypotension after initial fluid bolus (within 6 hrs)	Tocilizumab (8-12 mg/kg) IV
Use of low dose vasopressors for hypotension for longer than 12 hrs	Dexamethasone 5-10 mg IV/PO up to every 6 hrs with continued use of vasopressors
Initiation of higher dose vasopressors or addition of a second pressor for hypotension	Dexamethasone 5-10 mg IV/PO up to every 6 hrs with continued use of vasopressors
Initiation of oxygen supplementation	Tocilizumab (8-12 mg/kg) IV

Increasing respiratory support with concern for impending intubation	Dexamethasone 5-10 mg IV/PO up to every 6-12 hrs with continued use of vasopressors
Recurrence/Persistence of symptoms for which Tocilizumab was given ≥ 48 hrs after initial dose was administered	Tocilizumab (8-12 mg/kg) IV

6.4 Management of Non-CRS Toxicity Associated with Infused CAR T Cells into the CNS

When clinically prudent, subjects may be admitted to the hospital following the CAR T cell infusion for observation and monitoring if they exhibit minor symptoms which, if to worsen, could place the subject at risk of an adverse medical outcome.

Mild, transient symptoms have been observed greater than 24 hours after receiving CAR T cell treatment including fevers, headache, nausea, vomiting, and increase in neurologic changes from baseline. Suggested management of these symptoms is outlined below.

Fever may be managed with acetaminophen as clinically indicated. Avoid the use of NSAIDs. Consider obtaining blood culture and/or initiation of antibiotic therapy based upon clinical symptoms and institutional standards.

Headache may be managed with acetaminophen. If unresponsive to acetaminophen, consider magnesium, diphenhydramine/metoclopramide, NS fluid, and/or narcotics.

Nausea and vomiting may be treated with ondansetron. If unresponsive to ondansetron, consider diphenhydramine/metoclopramide, scopolamine, lorazepam, and/or olanzapine.

Seizures may require a medical evaluation with recommended neurology consult, hospital admission, and appropriate anti-seizure medication(s). Dexamethasone (10 mg x 1 dose) and neuroimaging should be considered.

Neurologic changes may be treated with symptomatic care and a review of the anti-epileptic therapy is recommended. Discussion with the PI, PI designee, and/or neuro-oncologist is recommended. For subjects with ≥ Grade 2 neurologic changes, the addition of corticosteroids should be considered, with initial dosing recommendations of dexamethasone 5-10 mg IV every 6 to 12 hours, often until resolution of symptoms. Bevacizumab may be used based on clinical judgment. Cytokine-directed therapies may be used based on clinical judgment. Cerebrospinal fluid assessments and CNS imaging should be considered as clinically indicated. See Section 7.16 Correlative Sciences for Correlative Sciences requirements during periods of systemic toxicity possibly, probably, or definitely related to CAR T cells.

Subjects who develop other serous medically significant toxicity attributable to the infused CAR T cell product will also be hospitalized for observation and treatment.

In addition, subjects who develop a new toxicity ≥ Grade 3 that is possibly, probably, or definitely attributable to the CAR T cell infusion other than those noted above, meets the definition of a DLT in Section 5.2, and, in the opinion of the Principal Investigator (PI) or PI

designee puts the subject at significant risk of an untoward outcome if measures are not taken to ameliorate the toxicity, should be given corticosteroids and/or Tocilizumab.

Table 6-3 Recommended Symptom Management for Non-CRS Toxicity

Symptom related to Non-CRS Toxicity	Suggested Intervention
Fever > 38.2°C	• Acetaminophen
Headache	• Acetaminophen<br>• Magnesium<br>• Diphenhydramine/metoclopramide<br>• NS fluids
Nausea/vomiting	• Ondansetron<br>• Diphenhydramine/metoclopramide<br>• Lorazepam<br>• Olanzapine
Seizures	• Anti-seizure medication<br>• Dexamethasone (10 mg) up to every 6 hours
Neurologic changes (≥ Grade 2)	• Anti-seizure medication<br>• For Arm C: bevacizumab (10 mg/kg) every 2 weeks up to 2 doses

6.5 Ablation of T Cells with Cetuximab

Cetuximab therapy may be initiated to ablate CAR expressing T cells in subjects under the following circumstances:

- Subject is experiencing a toxicity that meets the definition of DLT in Section 5.2 Dose-Limiting Toxicity (DLT), lasts ≥ 48 hours, AND, in the opinion of the PI or designee, cannot be controlled and puts the subject at significant risk of an untoward outcome if measures are not taken to ameliorate the toxicity, OR
- Subject is experiencing any duration or grade of toxicity which, in the opinion of the PI or designee, cannot be controlled and puts the subject at significant risk of an untoward outcome if measures are not taken to ameliorate the toxicity, OR
- Subject molecular studies indicate a lymphoproliferative disorder arising from the CAR T cells

The Study Chair or designee must be consulted prior to administering cetuximab.

Cetuximab is to be administered according to the current package insert.

Pretreatment with diphenhydramine is recommended 30-60 minutes before each dose of cetuximab (recommended diphenhydramine dosing: 1 mg/kg, maximum dose of 50 mg).

Cetuximab dosing recommendations are as follows:

- Subjects age ≥ 18 years, based on FDA approved dosing: loading dose of 400 mg/m^2 IV, followed by additional weekly doses of 250 mg/m^2 IV, if required
- Subjects age < 18 years: 250 mg/m^2 IV administered over 1 hour weekly [92]

If after 2 cetuximab doses are administered, there is no appreciable decrease in the CAR T cell burden, no further doses will be given. For subjects who have an appreciable response to cetuximab, up to a total of 4 weekly doses may be given. Any further cetuximab therapy following the 4 doses, if deemed necessary by the Sponsor and PI, will be administered only after Sponsor consultation with the FDA.

For all subjects who receive cetuximab, Correlative Sciences samples (peripheral blood and CSF) will be collected to determine CAR T cell persistence.

All Correlative Sciences samples drawn in response to cetuximab administration have a ± 3 day window, however a distinct sample must be drawn for each time point (samples may not be shared across time points: for instance, 1 sample drawn on day 4 following cetuximab dosing could not be used for both Day 1 and Day 3 despite being within the draw window). Refer to the study specific lab manual for specimen requirements of all correlative studies samples:

- *Peripheral Blood (all arms)*: Peripheral blood for Correlative Sciences should be collected prior to the initial dose of cetuximab and again on days 1, 3, 7, 10, 14, and 28 following the initial dose of cetuximab.

- *Cerebrospinal Fluid (Arm A)*: CSF for Correlative Sciences should be collected prior to the initial dose of cetuximab and again at Day 7 following the initial dose of cetuximab.

- *Cerebrospinal Fluid (Arms B and C)*: CSF for Correlative Sciences should be collected prior to the initial dose of cetuximab and again on days 1, 3, 7, 10, 14, and 28 days following the initial dose of cetuximab.

Additional measures may also be taken to resolve toxicity should the protocol-specified cetuximab treatment plan fail to abate the side effects associated with the CAR T cell product such as, but not limited to, immunosuppressive medications or agents (such as ATG/Campath and calcineurin inhibitors) or chemotherapy agents with immunosuppressive properties.

Table 6-2 details adverse events observed in clinical trials using cetuximab [90-94].

Table 6-2 Adverse Events Observed in Clinical Trials with Cetuximab		
**Common**	**Less Common**	**Rare**
• Abdominal pain<br>• Anemia<br>• Constipation<br>• Cough<br>• Diarrhea<br>• Difficulty breathing<br>• Fatigue<br>• Headache<br>• Hypomagnesemia<br>• Infection<br>• Leukopenia<br>• Liver enzyme elevation<br>• Nail changes<br>• Nausea/vomiting<br>• Neutropenia<br>• Peripheral neuropathy<br>• Skin reactions<br>• Stomatitis<br>• Weakness<br>• Weight loss	• Anxiety<br>• Bone pain<br>• Chills<br>• Confusion<br>• Dehydration<br>• Depression<br>• Dry mouth<br>• Eye irritation<br>• Fever<br>• Infusion reaction<br>• Insomnia<br>• Hypocalcemia<br>• Hypokalemia<br>• Joint pain<br>• Thrombocytopenia<br>• Upset stomach	• Cardiac abnormality<br>• Severe infusion reaction leading to death

7 STUDY PROCEDURES AND ASSESSMENTS

Refer to Appendix 2 – Schedule of Procedures, for a summary of required study activities.

7.1 Informed Consent/Assent

Prior to conducting any tests or procedures performed solely for the purposes of the study, written informed consent, and assent if applicable, must be obtained from the subject and/or subject's legal representative.

7.2 Demography

Demographic information (including date of birth, sex, race and ethnicity) will be transferred from the Immunotherapy Registration Portal at Screening.

7.3 Medical History

Relevant medical history, including history and treatment of CNS disease, will be obtained per the Schedule of Procedures.

7.4 Performance Status

Lansky (for subjects < 16 years of age) or Karnofsky (for subjects ≥ 16 years of age) performance status will be assessed per the Schedule of Procedures. Subjects who are unable to walk because of paralysis, but who are up in a wheelchair, will be considered ambulatory for purposes of assessing performance status. Reference Appendix 3 – Performance Status Scales.

7.5 Physical Examination, Vital Signs, Weight, Height

Physical examinations, vital signs (must include pulse, respiratory rate, blood pressure and temperature), weight and height will be done per the Schedule of Procedures.

7.6 Pulse Oximetry

Pulse oximetry will be performed as noted in the Schedule of Procedures.

7.7 Neurologic Exam

A neurological exam will be performed as indicated in the Schedule of Procedures.

7.8 Concomitant Medications

All concomitant medication and therapies will be documented at time of apheresis and then from the time of initial CAR T cell infusion through 28 days following final CAR T cell infusion or removal from protocol therapy, whichever occurs earlier. IV contrast is not considered a concomitant medication.

7.9 Adverse Events

Information regarding AEs will be captured following the start of the CAR T cell infusion through 28 days following final CAR T cell infusion per Section 11 Adverse Events and Serious Adverse Events. Related AEs will continue to be followed past Day 28 through resolution, if possible. This study will utilize the NCI CTCAE v5 for toxicity reporting and grading, except for CRS which will be assessed using the CRS Grading Scale in Section 11.5 Cytokine Release Syndrome Grading. Individual neurologic symptoms should be graded using CTCAE v5, however cumulative neurologic toxicity will be graded per Appendix 4 – Neurologic Toxicity Grading System. The study team should have access to the CTCAE v5 which can be downloaded from the Cancer Therapy Evaluation Program (CTEP) website (http://ctep.cancer.gov). Refer to Section 11 for information on reporting AEs.

7.10 Pregnancy Test

Female subjects of childbearing potential will have urine or serum pregnancy tests as indicated in the Schedule of Procedures.

Additional pregnancy tests will be performed at any visit in which pregnancy status is in question. A serum pregnancy test will be performed in the event of a positive or equivocal urine pregnancy test result.

7.11 Hematology

Hematology must include complete blood count (CBC) including hemoglobin, hematocrit, red blood cell count, white blood cell count, white blood cell differential, and platelet count and will be obtained according to the Schedule of Procedures.

7.12 Chemistry

Chemistry must include serum sodium, potassium, chloride, bicarbonate, blood urea nitrogen (BUN), creatinine, alanine aminotransferase (ALT), total bilirubin, conjugated bilirubin, and C-reactive protein (CRP) and will be obtained according to the Schedule of Procedures.

7.13 Cytokine Release Syndrome Labs and Evaluation

CRS labs and evaluation will be obtained daily during periods of Grade 2-4 CRS. CRS labs must include CRP, LDH, PT, PTT, ferritin, d-dimer, fibrinogen and absolute lymphocyte count. CRS evaluation must include temperature, heart rate and blood pressure.

7.14 Virology

Virology must include human immunodeficiency virus (HIV) antigen and antibody, Hepatitis B surface antigen, and Hepatitis C antibody testing and will be performed per the Schedule of Procedures. If Hepatitis C antibody testing is positive, quantitative PCR will be performed. Results of virology testing obtained up to 3 months prior to enrollment will be accepted for subject eligibility.

7.15 Cerebrospinal Fluid (CSF) Sampling

Cerebrospinal fluid sampling via intraventricular catheter will be performed according to institution standard practice at the time points indicated in the Schedule of Procedures. All CSF samples may be obtained via CNS catheter. If CSF collection via catheter is not feasible the sample is not required.

Specific testing requirements are noted in the Schedule of Procedures.

Per Section 6.4 Management of Non-CRS Toxicity Associated with Infused CAR T Cells into the CNS, in subjects who develop neurologic toxicity additional cerebrospinal fluid assessments should be considered if clinically indicated.

7.16 Correlative Sciences

Peripheral blood and CSF samples will be collected at the time points specified in the Schedule of Procedures and sent to the CSL. Refer to the study-specific Laboratory Manual for specimen type, storage, and shipping requirements.

Specimens may be processed for molecular analysis of T cell persistence and/or flow cytometric analysis based on detection of EGFRt in conjunction with T cell surface markers. These samples may also be used to detect the *in vitro* anti-B7-H3 activity of the persistent CAR T cells and for serum cytokine analysis. Additionally, DNA-based testing may be done to detect low levels of malignant cells.

If a subject develops systemic toxicity possibly, probably, or definitely (Section 6 Management of Toxicities and Complications) related to CAR T cells, peripheral blood for may be requested to be sent to the CSL up to once per day while the subject is experiencing toxicity. The blood may be used for serum cytokine analysis, and, if indicated by clinical data, detection of an anti-CAR or EGFRt immune response.

If a neurologic toxicity occurs, CSF samples may be requested to be sent the CSL for cytokine analysis and presence of CAR T cells.

Any material in excess of that needed for protocol required CSL samples will be retained for potential additional analysis related to this trial.

Archival tumor tissue, tumor biopsy/resection, or normal tissue biopsy material obtained for clinical purposes may be sent to the CSL to be tested for evaluation of B7-H3 expression, presence of CAR T cells, evaluation of immune modulation, or additional analysis related to this trial.

7.17 CNS Imaging

MRI brain and spine will occur according to the Schedule of Procedures. Per Section 6.4 Management of Non-CRS Toxicity Associated with Infused CAR T Cells into the CNS, additional CNS imaging should be considered in subjects who develop neurologic toxicity, if clinically indicated.

After month 12 following the final CAR T cell infusion MRI will be performed only as clinically indicated. For all MRIs that occur after the End of Therapy/Early Discontinuation Visit, MRI of the spine is required only for subjects with previous spinal disease, positive CSF cytology, or cause for clinical concern.

7.18 Disease Response

Disease Response will be assessed according to the Schedule of Procedures and as further described below.

7.18.1 Disease response assessment during active treatment

Disease Response will be assessed within 1 week following the last CAR T cell infusion on even courses (e.g., 2, 4...) but may be delayed up to 4 weeks due to clinical or scheduling issues. This evaluation must be performed prior to receiving Week 1 CAR T cell infusion on the subsequent odd course (e.g., 3, 5…).

7.18.2 Disease response assessment following active treatment

Disease Response will be assessed per the Schedule of Procedures at end of therapy/early discontinuation (i.e., the required assessment performed after final CAR T cell infusion), then at months 3, 6, 9, and 12 following final CAR T cell infusion as clinically indicated. After month 12 following the final CAR T cell infusion MRI will be performed only as clinically indicated.

Disease Evaluations are no longer required if progressive disease was confirmed in a prior evaluation.

7.19 Post-Treatment Long-Term Follow-Up data collection

Research participants who receive engineered T cells will be required to participate in long-term follow-up (LTFU) per the guidelines set forth by the FDA's Biologic Response Modifiers Advisory Committee that apply to gene transfer studies to capture delayed adverse events related to the use of lentivirally transduced T cells.

7.19.1 Post-treatment long-term follow-up data collection

For the first 5 years following treatment with CAR T cells, patients will be seen at least yearly. The visit will include an interval medical history and physical exam, specifically eliciting for the development of delayed, related AEs of interest, i.e., malignancy, neurologic disorder, rheumatologic or autoimmune disorder, or hematologic disorder. Unexpected medical problems felt to be related to the CAR T cells will be collected. If subjects are not able to be seen at study site, they may be seen by their primary physician for a physical exam and a blood draw courier kit will be provided to obtain research samples. The physician will be provided with adverse event screening guidelines and will be requested to notify the study team of all new malignancies and unexpected illness felt to be related to the CAR T cells.

Following the first 5 years of LTFU, patients who do not have evidence of persisting gene modified T cells will be contacted yearly by phone for an additional 10 years (for a total of 15

years of LTFU). For those patients who have continued persistence of transferred T cells, they will continue to be seen yearly for LTFU for at least a total of 15 years.

7.19.2 Post-Treatment Long-Term Follow-up Research Testing

At time points specified in the schedule of procedures, up to 15 mL of peripheral blood will be drawn and sent to CSL and, if relevant, will be tested for persistence of CAR T cells, clonality, and replication competent lentivirus (RCL) through year 1. During years 2 through 15, samples will be drawn and sent annually only for those subjects with evidence of persistent engraftment of the CAR T cells, or who test positive for RCL. If a patient receives a different lentivirus gene therapy product following treatment on BrainChild-03, they will be expected to have RCL testing for that product and will no longer have specimens collected on BrainChild-03 specifically for RCL.

Persistence: PCR for the transgene vector sequence and/or flow cytometric analysis may be done on mononuclear cells to determine the persistence of CAR T cells. Persistence assay testing will be discontinued if patient has had a negative test post their final infusion.

Clonality: Patients who at any time point in long term follow up have a >5% increase of cells positive for transgene expression will have a repeat test in 1 month. If the % of cells positive for transgene expression continues to increase, additional testing for clonality will be performed. Clonality may be measured by either examining integration sites or TCR diversity among vector positive cells. If there is evidence of clonality, repeat testing will be performed no later than 3 months.

Replication Competent Lentivirus (RCL): Evidence for RCL will be done using VSVg qPCR pre-infusion and at each post-infusion time point during the first year (3, 6 and 12 months). If all post-treatment assays are negative during the first year, then sample collection will be discontinued. For those subjects with positive RCL testing at any time during the 1st year, further time points will be determined by sponsor after review with FDA.

7.19.3 Autopsy

If the research participant dies while on study, then an autopsy will be requested.

8 REMOVAL FROM PROTOCOL THERAPY & OFF STUDY CRITERIA

8.1 Removal from Protocol Therapy

A subject may be discontinued from protocol therapy at any time. Subjects will be removed from protocol therapy for the following reasons:

- Subject/family or the investigator feels that it is not in the subject's best interest to continue
- Subject/family is non-compliant with protocol therapy and/or clinic appointments
- Subject receives medical intervention prohibited under this protocol or which will interfere with the ability to assess toxicity or response following CAR T cell infusion
- Subject experiences a DLT following CAR T cell infusion
- Subject does not meet criteria to receive the next CAR T cell infusion as defined in Section 5.5.2
- Subject is pregnant
- Subject meets criteria for removal from study (Section 8.2)

All subjects who discontinue protocol therapy should come in for an End of Therapy/Early Discontinuation Visit per Appendix 2 – Schedule of Procedures and should be encouraged to complete all remaining scheduled visits and procedures.

8.2 Off-Study Criteria and Study Termination

All subjects are free to withdraw from participation at any time, for any reason, specified or unspecified, without prejudice.

Reasonable attempts will be made by the investigator to provide a reason for subject withdrawals. The reason for the subject's withdrawal from the study will be recorded in the subject's source documents.

Subjects will be removed from the study for the following reasons:

- Subject, or subject's parents/legal guardian, withdraw consent for further participation
- Subject never received CAR T cell product
- Enrolled onto a long-term follow-up protocol for gene therapy
- 15-year anniversary of final CAR T cell infusion
- Death
- Lost to follow-up

The study may be terminated at any time by the Sponsor, the SCH Institutional Review Board (IRB), or the FDA.

9 STATISTICAL CONSIDERATIONS

9.1 Accrual and Study Duration

Up to 90 patients will be enrolled into BrainChild-03 at Seattle Children's Hospital. Anticipated duration of enrollment accrual is 6 years. Anticipated duration of the active treatment portion of the study is up to 7 months, and anticipated duration of the long-term follow-up period is 15 years.

9.2 Primary Objectives

The primary objectives are:

- To assess the feasibility of CNS locoregional adoptive therapy with autologous $CD4^+$ and $CD8^+$ T cells lentivirally transduced to express a B7-H3-specific CAR, EGFRt and a methotrexate resistant human dihydrofolate reductase mutein, delivered by an indwelling catheter into the tumor cavity or ventricular system in children and young adults with DIPG, DMG, or recurrent/refractory CNS tumors.
- To assess the safety of CNS locoregional adoptive therapy with autologous $CD4^+$ and $CD8^+$ T cells lentivirally transduced to express a B7-H3-specific CAR, EGFRt and a methotrexate resistant human dihydrofolate reductase mutein, delivered by an indwelling catheter into the tumor cavity or ventricular system in children and young adults with DIPG, DMG, or recurrent/refractory CNS tumors.
- To establish the tolerability of a fractionated CNS-delivered B7-H3 CAR T cell infusion schedule employing intra-subject dose escalation in children and young adults with DIPG, DMG, or recurrent/refractory CNS tumors.
- To define the maximally tolerated dose (MTD) and Recommended Phase 2 Dose Regimen (RP2DR) of CNS-delivered fractionated B7-H3 CAR T cell infusions.

Feasibility will be described across all subjects and within each therapy Arm and assessed as ability to achieve criteria noted above versus inability to meet each criterion.

Feasibility data will include:

- Number and percent of subjects with sufficient therapeutic product generated to receive two courses at the intended DL per assigned DR after two attempts using a single apheresis product for starting material
- Number of subjects who meet criteria for initial CAR T cell infusion per Section 5.5.1 Requirements for initial CAR T cell infusion
- Number of subjects who meet criteria for at least two courses of CAR T cell infusions per Section 5.5.2 Requirements for subsequent CAR T cell infusion and dose modification

Safety and tolerability data will include:

- History and physical exam (PE) occurring on study and occurring after the CAR T cell infusion
- Laboratory/radiographic evaluations taking place on study and occurring after CAR T cell infusions
- Adverse events

Within each Arm, the toxicities observed with each DR will be summarized in terms of type (organ affected or laboratory determination, such as ANC), severity (by NCI CTCAE v5), CRS grading, neurologic grading, and nadir or maximum values for the laboratory measures, date of onset, and attribution. Tables will be created to summarize these toxicities by Arm and DR with both number of subjects and number of incidences. The evaluation taking place within 2 days prior to initial CAR T cell infusion will serve as the baseline measurement.

9.3 Secondary and Exploratory Objectives

The secondary objectives are:

- To assess B7-H3 CAR T cell distribution within the cerebrospinal fluid (CSF) and the extent to which B7-H3 CAR T cells egress into the peripheral circulation.
- To assess disease response to B7-H3 CAR T cell locoregional therapy in children and young adults with DIPG, DMG, or recurrent/refractory CNS tumors.

Data for evaluation of secondary objectives will include:

- CAR T cell egress will be assessed by analysis of peripheral blood and CSF.
- Tumor response will be assessed using disease response criteria (Section 10.1).
- The duration of overall response will be measured from the initial complete response (CR) or partial response (PR) (whichever is first recorded) until the first date treatment failure is clinically or objectively documented.
- Survival endpoints: progression-free survival (PFS), overall survival (OS), and non-relapse mortality (NRM)

The exploratory objectives are:

- To evaluate for presence of B7-H3 CAR T cells in tumor tissue and/or normal tissue if a tissue biopsy, tumor biopsy, or resection is clinically indicated post-treatment.
- To evaluate B7-H3 expression in tumor tissue and/or normal tissue if a tissue biopsy, tumor biopsy, or resection is available.
- To analyze blood, CSF, and tumor tissue for biomarkers of anti-tumor B7-H3 CAR T cell expression, safety, and activity.

Data for evaluation of exploratory objectives will include:

- B7-H3 status of primary versus recurrent/refractory tumor will be determined by B7-H3 immunohistochemistry (IHC) performed on sequential tumor samples available for each patient.

9.4 Statistical Analysis

Descriptive statistics, such as mean, standard deviation, and range for continuous variables, and percent and number for categorical variables, will be summarized for baseline information (eg, the extent of prior therapy), and demographic information for each Arm singly and combined. This analysis includes the following variables: race, sex, age, height and weight, stage of disease, tumor characteristics at diagnosis, and others if applicable.

9.5 Safety and Tolerability

Subjects receiving any CAR T cell product will be eligible for safety and tolerability analyses. If a subject is removed from study prior to receiving the initial CAR T cell infusion, they will be replaced. For the purpose of assessing overall safety and tolerability, subjects will be evaluated for toxicities for up to 28 days following final CAR T cell infusion. For the purposes of escalation of DR and defining Maximal Tolerated Dose Regimen (MTDR), subjects in the existing cohort will be evaluated for toxicities from the time of initial CAR T cell infusion through 7 days following the final CAR T cell infusion received during Course 2, or until they commence with alternate therapies for their CNS tumor, whichever comes first. Subjects who are taken off study prior to Day 28 of Course 2 and have experienced a DLT will count towards that DR's evaluable accrual and will not be replaced. For all Arms, if a subject is removed from study prior to Day 28 of Course 2 for reasons other than toxicity (e.g., progressive disease requiring immediate alternative therapy) and has not received at least one dose of the maximally intended dose level per the assigned DR, they will be replaced before cohort advancement is permitted. Subjects who die before Day 28 of Course 2, and before receiving alternative therapy, and whose death is due to CAR T cell related toxicity will meet the definition of DLT and will not be replaced. Subjects who die before Day 28 of Course 2 due to reasons other than CAR T cell related toxicity and have not experienced a DLT will be replaced before cohort advancement is permitted.

9.6 Survival Definitions

OS: Time from CAR T cell infusion to death from any cause. If a subject is alive at the last evaluation time period, survival time is censored at the time of last follow-up.

PFS: Time from CAR T cell infusion to the first observation of disease progression or death from any cause, whichever occurs first. If the subject has not progressed, relapsed or died, progression-free survival is censored at the time of last follow-up.

NRM: Time to death event where cause of death is not attributable to underlying disease. Relapse and progression, including death attributable to underlying disease, are treated as competing risks for NRM and censored at time of last follow-up for those who do not relapse, progress or die.

Analysis of survival endpoints will include all subjects who receive at least one B7-H3 CAR T cell infusion and will be summarized for all subjects and by cohort. PFS and OS will be assessed using Kaplan Meier (K-M) estimates, and NRM will be assessed using cumulative incidence estimates. The following will be presented for both PFS and OS:

- K-M curves
- The median K-M expected survival time and 95% confidence interval
- Six-month and 12-month K-M survival estimates with 95% confidence intervals

The following will be presented for NRM:

- Cumulative Incidence curves
- Median expected survival and 95% confidence interval
- Six-month and 12-month and 95% confidence intervals

9.7 Evaluability for Dose Escalation

A subject will be considered DR escalation-evaluable if they are evaluable for toxicity (see Section 9.5 Safety and Tolerability), and were counted as part of a 3-subject dose escalation cohort according to the rules described in Section 9.9.1 Arms and DLT observation periods during Course 1 and 2.

9.8 Evaluability for Disease Response

A subject will be considered evaluable for disease response if: (1) the subject meets the eligibility criteria to receive the CAR T cell infusion; (2) the subject receives the CAR T cell infusion; and (3) the subject is under follow-up for a sufficient period to evaluate the disease and meets criteria for having evaluable disease (Section 10.1 Disease Response Criteria). For the purposes of the study, response will be assessed in comparison to the Treatment Response Evaluation performed within 28 days prior to the initial T cell infusion. Response will be characterized following Course 2 and, if available, at the completion of additional Courses. A subject who dies as a result of toxicity after receiving T cell product but prior to initial response assessment following Course 2 will be considered a non-responder.

9.9 Cohort Size and Rules for Cohort Advancement

9.9.1 Arms and DLT observation periods

Subjects will be enrolled onto one of three Arms, as described in Section 5.1.1 Treatment Arm assignment. The first 3 subjects treated will be enrolled only on Arm A or B and will be ≥ 15 years and ≤ 26 years at time of enrollment. Subjects may be enrolled on Arm C once 3 subjects have been treated on Arm A and/or Arm B, and at least 1 subject on Arm A or B completes Course 2 without experiencing a DLT. Within each Arm subjects will be assigned to successive DRs in Cohorts of up to 3 subjects. Dose escalation rules will be applied separately for each Arm. There will be a ≥ 7-day interval before enrollment of the initial subject onto the other

Arm. [For example, if the initial subject on Arm B is enrolled on 7 July 2018, the initial subject for Arm A cannot be enrolled until ≥ 14 July 2018.]

A modified 3+3 statistical design will be used for determining DR assignment with specific details below for determination of a Maximally Tolerated Dose Regimen (MTDR), followed by an expansion cohort. Within each Arm, treatment will begin with DR 1, Course 1. After the first subject completes Course 1 (i.e., completes the seven-day observation period following the last CAR T cell infusion of Course 1) the subsequent 2 subjects may be treated. Subjects within DR1 of each arm may only receive their initial CAR T cell infusion after the previously treated subject completes Course 1. Subjects enrolled in subsequent cohorts may only receive their initial CAR T cell infusion no sooner than 14 days following the date the previously treated subject received their initial CAR T cell infusion. Before the cohorts for the next (higher) DR may open, all 3 subjects of the prior (lower) DR must have received at least 1 of the maximally intended doses in Course 2 and completed a 7-day observation period following the final infusion occurring in Course 2 and not have experienced dose closure as noted below.

If the current Cohort is fully accrued, receiving therapy, but is not yet completely evaluated for DR DLTs, or if the cumulative number of DLTs at the current DR (including subjects still undergoing evaluation) exceeds 2, then a new subject cannot be assigned to the current DR. Instead, the subject may be offered (a) treatment at the DR below the DR currently being evaluated or (b) to wait until that current Cohort has been completely evaluated to receive treatment in the next DR as per study design. A subject treated under this provision may serve as a member of the current or the next cohort of three at that DR if the trial resumes regular accrual at that same DR or if the trial resumes at the next higher DR but subsequently de-escalates to revisit that DR.

9.9.2 Dose Regimen (DR) escalation rules

A subject will be considered DR escalation-evaluable if they are evaluable for MTDR DLT and are counted as part of a 3-subject dose escalation Cohort according to the rules described below.

The following rules will be applied separately and independently for each Arm. DLTs are defined in Section 5.2.

Dose escalation rules using a modified 3 + 3 design for Arms A, B, and C. DRs are detailed in Section 5.1.3.

1. Escalation and de-escalation decisions will use cohorts of 3 consecutive toxicity-evaluable subjects treated at the same DR
2. The dose escalation route will be similar to a 3+3 design, and will allow expansion on the tentative MTDR if 1 DLTs out of the first cohort of 3 and 1 DLTs out of the second cohort of 3 were observed
    a. If 0 DLTs out of the first cohort of 3 (0/3) within a DR, and there is a DR available for escalation, it will escalate to the next DR. If there is no DR

available for escalation, then this DR is considered as tentative MTDR and another cohort of 3 will be treated at the current DR.

b. If 1 DLTs out of the first cohort of 3 (1/3), a second cohort of 3 will be treated and evaluated at the current DR
    i. If 0 DLTs out of the second 3 and there is a DR available for escalation, it will escalate to the next DR. If there is no DR available for escalation (1 DLT in first 6), the MTDR will be estimated
    ii. If 1 DLTs out of the second cohort of 3 (i.e. 2 DLT in the first 6), a third cohort of 3 will be treated at the current DR. If there are 0 DLTs in the third cohort of 3, the MTDR will be estimated. If there is 1 DLT in the third cohort (i.e. 3 DLTs in the first 9), a final cohort of 3 will be treated at the current DR and the MTDR will be estimated once all subjects have been evaluated.

c. If 2 or more DLTs out of any cohort of 3 (≥2/3) toxicity-evaluable subjects, the current DR will be shut down and the DR will de-escalate to the previous DR. If the first 2 subjects of any cohort of 3 both experience DLTs and the third subject has not received CAR T cells, stop the cohort at 2 subjects and de-escalate to previous DR and the third subject will become the first subject of a new cohort. If there is no DR available for de-escalation (lowest), the arm will be shut down.

d. After de-escalation, if the current DR has fewer than 6 toxicity-evaluable subjects, then the requisite number of subjects will be treated and evaluated at that DR and the MTDR will be re-estimated. If the current DR has 6 or more than 6 toxicity-evaluable subjects, MTDR will be estimated.

9.9.3 Stopping rules

In the event of the following, further T cell infusions will be suspended pending review by the DSMB and consultation with the FDA:

- Death unrelated to disease progression or accidental trauma that occurs within 30 days of CAR T cell administration

To further protect the safety of patients, infusions will also be suspended pending review by the DSMB and consultation with the FDA if the lower bound of a 1-sided 80% exact binomial confidence interval of the discontinuation rate due to medically significant Grade 4 or greater events that are possibly, probably, or definitely attributable to CAR T cells is > 10%. Operationally, any of the following would trigger such a rule: 2 out of the first ≤ 5 subjects or 3 out of the first ≤ 11 patients or 4 out of the first ≤ 18 patients or 5 out of the first ≤ 24 have unacceptable toxicity or ≥ Grade 4 AEs that are possible, probably, or definitely attributable to CAR T cells. If the true probability of ≥ Grade 4 AE is 2.5%, the probability of study suspension under the above rule is approximately 0.007; if the true probability is 30%, the probability of suspension is approximately 0.93 (probabilities estimated from 5,000 simulations).

This rule will apply to medically significant (requiring medical intervention) ≥ Grade 4 toxicities that are possibly, probably, or definitely attributable to CAR T cells, with the exception of Grade 4 CRS for < 72 hours, and Grade 4 fever.

If this rule is met, further T cell infusions for all subjects enrolled will be immediately halted. A safety evaluation will be performed and reviewed with the FDA and DSMB prior to resuming any CAR T cell infusions.

Additionally, the Study Chair may shut down a DR at any time due to toxicities observed or efficacy observed at lower DRs.

9.9.4 Definition and determination of MTDR and RP2DR

The MTDR is defined as the highest DR with at least six DLT-evaluable subjects, whose cumulative DLT rate during Courses 1 and 2 is below 34%. The MTDR will be estimated separately for each Arm.

Per investigator discretion and with DSMB approval, if a DR lower than the selected MTDR displays satisfactory disease response and has at least six DLT-evaluable subjects, then this DR can be chosen as the RP2DR instead of the MTDR.

9.10 Expansion Cohort

Following determination of the safety and toxicity of each arm based on our modified 3+3 design, an expansion cohort of up to 12 subjects in each arm will be started with treatment at the MTDR/RP2DR. The purpose of the expansion cohort is to provide a more precise assessment of the tolerability and toxicity of this treatment when dose at the MTDR/RP2DR as well as gain additional knowledge regarding secondary and exploratory objectives with particular focus on the duration and magnitude of in vivo persistence of CAR T cells in the cerebral spinal fluid including descriptions of cellular and protein components.

The expansion cohort will begin once the MTDR/RP2DR has been determined. For the expansion cohort, up to 12 additional subjects will be treated. Accrual to the expansion cohort will be continuous and will not depend upon the completion of treatment of the previous patients in the expansion cohort. Ongoing safety monitoring will occur during the expansion cohort with analysis in combination with all subjects treated at the MTDR/RP2DR. If at any time the DLT rate appears to exceed 33%, infusions would be halted to allow review with the Data Safety Monitoring Board (DSMB). Examples include a DLT rate of 3/7-9, 4/10-12, 5/13-15, 6/16-18, or 7/19-21.

9.11 Safety Monitoring

9.11.1 Weekly safety review

The Study Chair, Investigators, study statistician (if needed), CRAs, research nurses, and other site and Sponsor staff will meet weekly (or as needed when there are active subjects) to review subject enrollment and conduct subject safety review. This group is responsible for monitoring

the data and safety of this study, including implementation of the stopping rules for dose escalation. During this meeting, this group will review as applicable enrollment, AEs, DLTs, and protocol compliance, CAR T cell persistence analysis, feasibility data and follow-up information for each subject.

9.11.2 Data Safety Monitoring Board

The study will be monitored by a DSMB. This is an independent committee with no affiliation to the protocol. The DSMB will meet prior to the study opening to review and approve the study protocol and DSMB Charter. The DSMB will review toxicity data approximately every 6 months. In addition, the DSMB will review study conduct including accrual, drop-outs, data completeness, any inability to generate CAR T cell product that meets all Quality Control criteria, positive bacterial cultures from the CAR T cell product, subjects not meeting eligibility criteria for CAR T cell infusion after enrollment, protocol compliance, and treatment efficacy measures.

DSMB meetings may be called at any time by the DSMB chair or sponsor for additional safety review if indicated. Following any temporary suspension of accrual for a safety event, the DSMB will be convened and will further review the safety data to determine if continuation of accrual is appropriate. Applicable regulatory agencies will receive copies of the DSMB's recommendations as they become available.

10 DISEASE RESPONSE

10.1 Disease Response Criteria

10.1.1 Measurable disease

Measurable disease is defined as a lesion that is at minimum 10 mm in 2 dimensions on standard MRI.

10.1.2 Evaluable disease

Evaluable disease is defined as a lesion or multiple lesions, with no lesion that can be accurately measured in at least 2 dimensions and does not meet criteria to be measurable. Such lesions may be evaluable by pathology, MRI, immunocytochemistry techniques, tumor markers, CSF cytology, or other reliable measures.

10.1.3 Selection of target and non-target lesions

For most CNS tumors, only 1 lesion/mass is present and therefore is considered a “target” for measurement/follow up to assess for tumor progression/response. If multiple measurable lesions are present, up to 5 may be selected as target lesions. Target lesions should be selected on the basis of size and suitability for accurate repeated measurements. All other lesions will be followed as non-target lesions. The lower size limit of the target lesion(s) should be at least twice the thickness of the slices showing the tumor to decrease the partial volume effect (e.g., 8 mm lesion for a 4-mm slice).

The presence of non-target lesions should be noted.

10.1.4 Response criteria for target lesions

Complete Response (CR): Disappearance of all target lesions

Partial Response (PR): ≥ 50% decrease in the sum of the products of the two perpendicular diameters of all target lesions (taking as reference the initial baseline measurements)

Stable Disease (SD): Neither sufficient decrease in the sum of all target lesions to qualify for PR, nor sufficient increase to qualify for progressive disease

Progressive Disease (PD): ≥ 25% increase in the sum of the products of the perpendicular diameters of the target lesions (taking as reference the smallest sum of the products observed since the start of treatment)

Immune-Related Progressive Disease (irPD) may occur in the setting of CAR T cell therapy and can manifest as increased disease and/or new lesions and is distinct from PD [93]. irPD will be documented as SD. irPD must occur within 6 months of the initiation of immunotherapy, cannot last longer than 6 months, and cannot correspond to neurologic decline that is definitely attributable to worsening disease. If irPD is suspected:

- Re-imaging is recommended in 3 months and if progressive disease is confirmed, subject will be determined to have PD
- Biopsy should be considered and if pathology is consistent with progressive disease, subject will be determined to have PD

10.1.5 Response criteria for non-target lesions

Complete Response (CR): Disappearance of all non-target lesions

Stable Disease (SD): The persistence of one or more non-target lesions

Progressive Disease (PD): The appearance of one or more new lesions and/or unequivocal progression of existing non-target lesions

Immune-Related Progressive Disease (irPD) may occur in the setting of CAR T cell therapy and can manifest as increased disease and/or new lesions and is distinct from PD [93]. irPD will be documented as SD. irPD must occur within 6 months of the initiation of immunotherapy, cannot last longer than 6 months, and cannot correspond to neurologic decline that is definitely attributable to worsening disease. If irPD is suspected:

- Re-imaging is recommended in 3 months and if progressive disease is confirmed, subject will be determined to have PD
- Biopsy should be considered and if pathology is consistent with progressive disease, subject will be determined to have PD

10.1.6 Evaluation of best overall response

The overall response assessment takes into account response in both target and non-target lesions and the appearance of new lesions, according to the criteria described in the table below. The best overall response is the best response recorded from the start of the treatment until disease progression/recurrence (taking as reference for PD the smallest measurements recorded since the treatment started). In general, the subject's best response assignment will depend on the achievement of both measurement and confirmation criteria.

Table 10-1 Best Overall Response

Target lesions	Non-Target lesions	New Lesions	Overall response
CR	CR	No	CR
CR	SD	No	PR
PR	CR or SD	No	PR
SD	CR or SD	No	SD
PD	Any	Yes or No	PD
Any	PD	Yes or No	PD
Any	Any	Yes	PD

Subjects with a global deterioration of health status requiring discontinuation of treatment without objective evidence of disease progression at that time should be classified as having

"symptomatic deterioration." Every effort should be made to document the objective progression even after discontinuation of treatment.

In some circumstances, it may be difficult to distinguish residual disease from normal tissue. When the evaluation of complete response depends on this determination, it is recommended that the residual lesion be investigated (biopsy) to confirm the complete response status.

11 ADVERSE EVENTS AND SERIOUS ADVERSE EVENTS

11.1 Definition of Adverse Event (AE)

According to 21 CFR 312.32(a): "An adverse event is defined as any untoward medical occurrence associated with the use of a drug in humans, whether or not considered drug related." An AE can therefore be any unfavorable and unintended sign (including an abnormal laboratory finding, for example), symptom, or disease temporally associated with the use of an investigational product, whether or not related to the investigational products. AEs may also include pre- or post-treatment complications that occur as a result of protocol specified procedures, lack of efficacy, overdose, drug abuse/misuse reports, or occupational exposure. Pre-existing events that increase in severity or change in nature during or as a consequence of participation in the clinical study will also be considered AEs. The subject's baseline for purposes of assessing toxicity is established by the active medical conditions present directly prior to initiation of Course 1, Week 1 investigational product administration.

An AE does not include the following:

- Medical or surgical procedures such as surgery, endoscopy, tooth extraction, and transfusion. The condition that led to the procedure may be an adverse event and must be reported.
- Pre-existing diseases, conditions, or laboratory abnormalities present or detected before the infusion of investigational product that do not worsen.
- Situations where an untoward medical occurrence has not occurred (e.g., hospitalization for elective surgery, social and/or convenience admissions).
- Overdose without clinical sequelae.
- Any medical condition or clinically significant laboratory abnormality with an onset date before infusion of investigational product and not related to a protocol-associated procedure is not an AE. It is considered to be pre-existing and should be documented on the medical history CRF.
- Abnormal laboratory values or tests that are not clinically significant or do not require therapy.

A suspected adverse reaction is any AE for which there is a reasonable possibility that the investigational product caused the AE. For the purposes of IND safety reporting, "*reasonable possibility*" means there is evidence to suggest a causal relationship between the investigational product and the AE. *Suspected* adverse reaction implies a lesser degree of certainty about causality than adverse reaction, which means any AE caused by an investigational product.

Life-threatening AE or life-threatening suspected adverse reaction is an AE or suspected adverse reaction that, in the view of either the investigator or study Sponsor, places the study participant at immediate risk of death. It does not include an AE or suspected adverse reaction that, had it occurred in a more severe form, might have caused death.

11.2 Definition of Serious Adverse Event (SAE)

An AE or suspected adverse reaction is considered "serious" if, in the view of either the investigator or the study Sponsor, it results in any of the following outcomes:

- Death
- A life-threatening AE
- Inpatient hospitalization or prolongation of existing hospitalization
- Persistent or significant incapacity or substantial disruption of the ability to conduct normal life functions
- A congenital anomaly/birth defect
- A secondary malignancy

Important medical events that may not result in death, be immediately life-threatening, or require hospitalization may be considered serious when, based upon appropriate medical judgment, they may jeopardize the study participant and may require medical or surgical intervention to prevent one of the outcomes listed above.

11.3 Classification of an Adverse Event

11.3.1 Grading of adverse events

Wherever possible, all AEs will be graded using the NCI CTCAE v5, except grading of CRS which will be according to the CRS Grading Scale (Section 11.5 Cytokine Release Syndrome Grading). Individual neurologic symptoms should be graded using CTCAE v5, however cumulative neurologic toxicity will be graded according to the Neurologic Toxicity Grading System (Section 14, Appendix 4 – Neurologic Toxicity Grading System). The majority of AEs can be graded using these scales.

If an AE cannot be graded using the NCI CTCAE criteria, it should be graded as mild, moderate, severe, life-threatening, or death using the following definitions.

- ***Mild (Grade 1):*** Awareness of signs or symptoms, but easily tolerated and of a minor irritant type, causing no loss of time from normal activities. Symptoms do not require therapy or a medical evaluation. Signs and symptoms are transient.

- ***Moderate (Grade 2):*** Events that introduce a low level of inconvenience or concern to the participant and may interfere with daily activities but are usually improved by simple therapeutic measures. Moderate experiences may cause some interference with functioning.

- ***Severe (Grade 3):*** Events that interrupt the participant's normal daily activities and generally require systemic drug therapy or other treatment. They are usually incapacitating.

- ***Life-threatening (Grade 4):*** Events that place the participant at immediate risk of death or are disabling.

- ***Death (Grade 5):*** Events that result in death.

To make sure there is no confusion or misunderstanding of the difference between the terms "serious" and "severe," which are not synonymous, the following note of clarification is provided. The term "*severe*" is often used to describe the intensity (severity) of a specific event (as in mild, moderate, or severe myocardial infarction); the event itself, however, may be of relatively minor medical significance (such as severe headache). This is not the same as "*serious*", which is based on participant/event outcome or action criteria usually associated with events that pose a threat to a subject's life or functioning. Seriousness (not severity) serves as a guide for defining regulatory reporting obligations.

11.3.2 Relationship of adverse event to treatment

The investigator will assess the potential relationship of the AE to investigational product using the following descriptions.

- ***Not Related:*** This category applies to an AE that is clearly not related to the investigational agent/procedure, beyond a reasonable doubt. That is, another cause of the event is most plausible and/or a clinically plausible temporal sequence is inconsistent with the onset of the event and the exposure to study drug and/or a causal relationship is considered biologically implausible.

- ***Unlikely Related:*** This category applies to an AE that is doubtfully related to the investigational agent/procedure. That is, another cause of the event is highly likely and/or there is not a reasonable temporal sequence from administration of the study drug or one that follows a known or expected response pattern to the suspected study drug. The event could readily have been produced by a number of other factors.

- ***Possibly Related:*** This category applies to an AE that follows a reasonable temporal sequence from administration of the study drug and that follows a known or expected response pattern to the suspected study drug, but that could readily have been produced by a number of other factors.

- ***Probably Related:*** This category applies to an AE that is likely related to the investigational agent/procedure. That is, the AE has a temporal relationship to the administration of the investigational agent(s) or research intervention and follows a known or suspected pattern of response.

- ***Definitely Related:*** This category applies to an AE that is clearly related to the investigational agent/procedure. That is, the AE has a temporal relationship to the administration of the investigational agent(s) or research intervention and follows a known or suspected pattern of response.

11.4 Expectedness, Pre-Existing Conditions, and Persistent Adverse Events

- ***Expectedness:*** The study Sponsor will be responsible for determining whether an AE is expected or unexpected. An AE will be considered unexpected if the nature, severity, or frequency of the event is not consistent with the risk information previously described for the investigational product.

- ***Pre-Existing Conditions:*** If a pre-existing condition worsens in severity, the worsening may require reporting as an AE or SAE. In addition, if a condition present at baseline resolves and then recurs, the reoccurrence may require reporting as an AE or SAE.

- ***Persistent Adverse Events:*** A persistent AE is one that extends continuously, without resolution (is ongoing). A persistent AE is reported only once unless the grade and/or frequency become more severe. If the grade becomes more severe the original AE will be considered to have stopped on the date the severity increased and the AE must be reported again with the higher grade and/or frequency.

11.5 Cytokine Release Syndrome Grading

Grade[2]	Description of Symptoms
**1: Mild**	Not life-threatening, require only symptomatic treatment such as antipyretics and anti-emetics (e.g., fever[1] ≥ 38.0°C, nausea, emesis, fatigue, headache, myalgia, malaise)
**2: Moderate**	Require and respond to moderate intervention:<br>• Oxygen requirement for low flow nasal cannula[3] or blow by oxygen, or<br>• Hypotension responsive to fluids
**3: Severe**	Require and respond to aggressive intervention:<br>• Oxygen requirement for high flow nasal canula, face mask, non-rebreather or Venturi mask, and/or<br>• Hypotension requiring a one vasopressor with or without vasopressin
**4: Life threatening**	Life-threatening:<br>• Requirement for positive pressure support including ventilator support, CPAP or BiPAP<br>• Hypotension requiring multiple vasopressors (excluding vasopressin)
**5: Fatal**	Death

1: Fever is defined as temperature ≥38.0°C not attributable to any other cause. In patients who have CRS then receive antipyretics or anti-cytokine therapy such as tocilizumab or corticosteroids, fever is no longer required to grade subsequent CRS severity. In this case, CRS grading is driven by hypotension and/or hypoxia.

2: CRS grade is determined by the more severe event: hypotension or hypoxia not attributable to any other cause. For example, a patient with temperature of 39.5°C, hypotension requiring one vasopressor and hypoxia requiring low-flow nasal cannula is classified as having Grade 3 CRS. Organ toxicities associated with CRS may be graded according to CTCAE v5.0 but they do not influence CRS grading.

3: Low-flow nasal cannula is defined as oxygen delivered at ≤ 6 liters/minute. Low flow also includes blow-by oxygen delivery, sometimes used in pediatrics. High-flow nasal cannula is defined as oxygen delivered at > 6 liters/minute

11.6 Neurologic Toxicity Grading

Individual neurologic symptoms should be graded using CTCAE v5, however cumulative neurologic toxicity will be graded according to the Neurologic Toxicity Grading System, see Section 14, Appendix 4 – Neurologic Toxicity Grading System.

11.7 Serious Adverse Event Reporting

SAEs occurring from the beginning of the first infusion of the investigational product up to and including 30 days after the last infusion of the investigational product will be reported to the study Sponsor or its designee within 24 hours of the investigator becoming aware of the event. SAEs occurring after 30 days following the last treatment with the investigational product, which, in the judgement of the investigator or treating physician, are ***possibly***, ***probably or***

definitely related to treatment with the investigational product, will be reported to the study Sponsor or its designee within 24 hours of the investigator becoming aware of the event.

Initial SAE reports must be followed by detailed descriptions. These should include copies of hospital case records and other documents when requested. Telephone reports must be confirmed promptly by written report.

Additionally, the Investigator is responsible for submitting follow-up reports for all SAEs until the SAE has resolved or until the subject's condition stabilizes (in case of persistent impairment), or the study participant dies.

A death (including death due to progressive disease) occurring within 30 days after the last T cell infusion must be reported to the Sponsor or its designee within 24 hours of site awareness of the death whether or not it is considered treatment-related. Any death occurring greater than 30 days after the last T cell infusion must be reported to the Sponsor or its designee within 24 hours of site awareness of the death only if it is possibly, probably, or definitely related to treatment with the investigational product.

The investigator also must notify the IRB/Institutional Ethics Committee (IEC) and/or Institutional Biosafety Committee (IBC) of the occurrence of the SAE, in writing, as soon as is practicable and in accordance with local law. A copy of this notification must be provided to the study Sponsor or its designee.

11.7.1 Study-specific SAE reporting

Planned hospitalization will not be reported to the Sponsor as an SAE. Planned hospitalizations may include admission for:

- entry into the study
- elective treatment of a pre-existing condition
- routine monitoring of the subject not associated with any deterioration in condition
- monitoring following CAR T cell infusion if minor (non-serious) symptoms indicate the potential for more significant medical problems

11.8 IND Safety Reporting

This study will comply with 21 CFR 312.32, which requires that the sponsor notify the FDA and participating investigators in an IND Safety Report of potentially serious risks from clinical trials or any other source. Reports must be submitted no later than 15 calendar days after the Sponsor becomes aware of the information and determines it is reportable.

The Sponsor will submit an IND Safety Report for individual events meeting the following criteria:

- There is a reasonable probability the drug under study caused the event (ie, there is evidence to suggest a causal relationship between the drug and the AE)
- The event meets the criteria in Section 11.2 Definition of Serious Adverse Event (SAE) for a serious adverse event
- The event is unexpected (ie, it is not consistent with the risk information in the protocol or other information submitted to the FDA in the IND)

Analysis of one or more occurrences of the same event, and/or aggregate analysis of specific events, may lead the Sponsor to determine that information requires reporting via an IND Safety Report.

The period for IND safety reporting extends from the beginning of the first infusion of the investigational product up to and including 30 days after the last infusion of the investigational product.

11.9 On-Target, Off-Tumor Toxicities

On-target off-tumor toxicities are toxicities attributable to the direct effect of CAR T cells on tissue (e.g. organ damage caused by CAR T cell direct targeting of organ cells) and not associated with a more global syndrome attributable to the CAR T cell (eg, CRS or pseudoprogression).

11.10 Reporting of Pregnancy

Pregnancies in study participants or partners that occur from time of consent through 28 days following administration of investigational product or in subjects with continued persistence of CAR T cells (whichever is later) must be reported within 24 hours of site awareness of the event to the study Sponsor or its designee. Subjects who become pregnant prior to the initial administration of investigational product will be taken off study. If pregnancy in subject's partner occurs after administration of investigational product, the investigator must make every effort to follow the pregnancy of either the participant or partner through resolution of the pregnancy (delivery or termination) and report the resolution to the study sponsor or its designee. In the event of a pregnancy in the partner of a participant, the investigator should make every effort to obtain the female partner's consent for release of protected health information.

11.11 Safety Reporting Contact Information

An SAE Report Form will be provided by the Sponsor for sites to use in reporting SAEs to the Sponsor. The completed SAE Report Form must be returned to SCTx per the instructions on the form.

12 ADMINISTRATIVE, ETHICAL, AND REGULATORY CONSIDERATIONS

12.1 Good Clinical Practice

This protocol is written in accordance with the principles established by the 18th World Medical Assembly General Assembly (Helsinki, 1964) and subsequent amendments and clarifications adopted by the General Assemblies. The investigator will make every effort that the study described in this protocol is conducted in full conformance with those principles, current FDA regulations, ICH Good Clinical Practices (GCP) guidelines, and local ethical and regulatory requirements. Should a conflict arise, the investigator will follow whichever law or guideline affords the greater protection to the individual subject. The investigator will also make sure he or she is thoroughly familiar with the appropriate administration and potential risks of administration of the study drug, as described in this protocol, prior to the initiation of the study.

12.2 Institutional Review Boards (IRB) and Institutional Biosafety Committees (IBC)

The protocol and consent form, and any accompanying material to be provided to participants, will be reviewed and approved by the IRB/IEC and/or IBC of the participating center prior to study initiation. Serious adverse experiences regardless of causality will be reported to the IRB/IEC and/or IBC in accordance with the standard operating procedures and policies of the IRB/IEC and/or IBC, and the Investigator will keep the IRB/IEC and/or IBC informed as to the progress of the study. The Investigator will obtain assurance of IRB/IEC compliance with regulations.

Any documents that the IRB/IEC and/or IBC may need to fulfill its responsibilities (such as protocol, protocol amendments, Investigator's Brochure (IB), consent forms, information concerning participant recruitment, payment or compensation procedures, or other pertinent information) will be submitted to the IRB/IEC and/or IBC. The IRB/IEC's and/or IBC's written unconditional approval of the study protocol and the informed consent form will be in the possession of the Investigator before the study is initiated.

Before implementation, the investigator will submit to and receive documented approval from the IRB/IEC for any modifications made to the protocol or any accompanying material to be provided to participants after initial IRB/IEC approval, with the exception of those necessary to reduce immediate risk to study subjects.

The IRB/IEC must be informed of revisions to other documents originally submitted for review; serious and/or unexpected adverse experiences occurring during the study in accordance with the standard operating procedures and policies of the IRB/IEC; new information that may affect adversely the safety of the participants of the conduct of the study; an annual update and/or request for re-approval; and when the study has been completed.

Research modifications will be submitted to the IBC according to National Institutes of Health (NIH) guidelines, standard operating procedures and policies of the IBC.

12.3 Informed Consent/Assent and Other Informational Documents Provided to Study Participants

The Investigator will prepare the informed consent form, assent and the Health Insurance Portability and Accountability Act (HIPAA) authorization and provide the documents to the Sponsor or its designee for approval prior to submission to the IRB/IEC. The consent/assent form generated by the Investigator must be acceptable to the Sponsor and be approved by the IRB. The written consent documents will comply with the elements of informed consent as described in 21 CFR Part 50 and ICH E6, and will also comply with local regulations. The Investigator will send a copy of the IRB-approved Informed Consent/Assent Form to the Sponsor or its designee for the study file.

A properly executed, written, informed consent will be obtained from each subject prior to entering the participant into the trial. Information should be given in both oral and written form and participants or their legal representatives must be given ample opportunity to inquire about details of the study. If appropriate and required by the local IRB, assent from the subject will also be obtained. If a participant is unable to sign the informed consent form (ICF) and the HIPAA authorization, a legal representative may sign for the subject. A copy of the signed consent form (and assent) will be given to the participant or their legal representative and will be provided any new information during the course of the study that might affect their continued participation in the study.

If the protocol is amended and the ICF (and assent, if applicable) is revised, each participant or their legal representative will be required to provide written informed consent/assent again using the revised ICF (and assent, if applicable).

Receipt of written informed consent/assent will be documented in each potential participant's CRF. The signed ICF will remain in each participant's study file and must be available to the study monitor(s) at all times.

12.4 Data Handling and Record Keeping

12.4.1 Case report forms and source documents

The investigator is required to initiate and maintain, for each participant, an adequate and accurate case history that records all observations and other data related to the study for that participant. A validated electronic data capture system will be used for entry of the data into electronic Case Report Forms (CRFs). Data must be recorded on CRFs approved by the Sponsor or its designee. All information recorded on CRFs for this study must be consistent with the participant's source documentation.

Initial data entry and any changes to the data will be made only by SCRI-authorized users, and data entries and changes will be captured in an electronic audit trail. An explanation of any data change should be recorded in the CRF. All data entered in to the CRF must be verifiable; therefore, CRFs will be routinely checked for accuracy, completeness, and clarity and will be cross-checked for consistency with source documents, including laboratory test reports and

other subject records by the Sponsor or its designee. The investigator must allow direct access to all source documents.

12.4.2 Data quality assurance

Quality assurance will be monitored by the Sponsor/Sponsor's designee at appropriate intervals determined by multiple factors including, but not limited to, the assessed risk level of the study, subject enrollment, reporting deadlines, and the findings of previous monitoring visits.

The trial will comply with the standard guidelines set forth by applicable regulatory committees and other institutional, state, and federal guidelines.

12.4.3 Record retention

All study records must be retained for at least two years after the last approval of a marketing application in the US or an ICH region and until: 1) there are no pending or contemplated marketing applications in the US or an ICH region or 2) at least two years have elapsed since the formal discontinuation of clinical development of the investigational product under study. The investigator/institution should retain subject identifiers for at least 15 years after the completion or discontinuation of the study. Subject files and other source data must be kept for the maximum period of time permitted by the hospital, institution or private practice, but not less than 15 years. These documents should be retained for a longer period, however, if required by the applicable regulatory requirements or by a Seattle Children's Therapeutics agreement. Seattle Children's Therapeutics must be notified and will assist with retention should the investigator/institution be unable to continue maintenance of subject files for the full 15 years. All study records must be stored in a secure and safe facility.

12.5 Investigational Product Accountability

While at the clinical site, investigational product must be stored in a secure limited access location at controlled temperature as required and according to product packaging. The storage facility must be available for inspection by the study monitor at any time during the study. A drug accountability record must be maintained for all investigational product received, dispensed, returned, and/or lost during the study. This record must be kept current and made available to the study monitor for inspection.

12.6 Protocol Deviations

A protocol *deviation* is any change, divergence, or departure from the study design or procedures defined in the protocol. In general, protocol deviations are classified as either *major* (or "*important*" per ICH E3 Structure and Content of Clinical Study Reports — Questions and Answers R1) or *minor*.

Major or important protocol deviations are a subset of protocol deviations that might significantly affect the completeness, accuracy, and/or reliability of the study data or that might significantly affect a participant's rights, safety, or well-being. For example, *major* or *important protocol deviations* might include enrolling participants in violation of key eligibility criteria or

failing to collect data necessary to interpret primary objectives, as this may compromise the scientific value of the trial.

Minor protocol deviations are protocol deviations that do not have a substantive effect on the participant's rights, safety, or well-being or the integrity of the data. For example, *minor protocol deviations* might include a missed study visit "window."

Major or *important protocol deviations* of this study include, but are not limited to, the following reasons:

- Failure to meet inclusion/exclusion criteria
- Use of a prohibited concomitant medication
- Failure to comply with GCP guidelines

The Investigator will determine if a *major* or *important protocol deviation* will result in withdrawal of a subject. All deviations should be reported to the IRB/IBC in accordance to the IRB/IBC reporting requirements.

12.7 Subject-Specific Biologic Materials

Leftover CAR T cell product, serum samples, cryopreserved peripheral blood mononuclear cells (PBMC), CSF, and other tissue and material will become the property of the Sponsor and may be used in non-therapeutic experiments.

12.8 Investigator's Responsibilities

By signing the Agreement of Investigator page (**Appendix 1a – sponsor Signature Page**), the Principal Investigator agrees to:

- Conduct the study in accordance with the protocol and only make changes after notifying the Sponsor (or designee), except when to protect the safety, rights or welfare of subjects.
- Personally conduct or supervise the study (or investigation).
- Ensure that the requirements relating to obtaining informed consent and IRB/IEC review and approval meet federal guidelines, as stated in 21CFR parts 50 and 56.
- Report to the Sponsor or designee any AEs that occur in the course of the study, in accordance with 21 CFR 312.64.
- Ensure that all associates, colleagues and employees assisting in the conduct of the study are informed about their obligations in meeting the above commitments.
- Maintain adequate and accurate records in accordance with 21 CFR 312.62 and to make those records available for inspection with the Sponsor or its designee.
- Ensure that an IRB/IEC that complies with the requirements of 21 CFR 56 will be responsible for initial and continuing review and approval of the clinical study.

- Promptly report to the IRB/IEC and the Sponsor or its designee all changes in the research activity and all unanticipated problems involving risks to subjects or others (to include amendments and IND safety reports).
- Ensure IRB/IEC approval before any changes are made in the research study, except when necessary to eliminate immediate hazards to the participants/subjects.
- Comply with all other requirements regarding the obligations of clinical investigators and all other pertinent requirements listed in 21 CFR 312.

12.9 Publication Policy

The publication or presentation of any study results shall comply with all applicable privacy laws, including, but not limited to, HIPAA. Material must be reviewed and approved by the Sponsor prior to submission for publication.

12.10 Financing and Insurance

For participation at sites other than Seattle Children's Hospital, financing and insurance for this clinical trial will be addressed in clinical trial agreements with the study site.

13 REFERENCES

1. Ostrom, Q.T., et al., *CBTRUS statistical report: primary brain and central nervous system tumors diagnosed in the United States in 2007-2011.* Neuro Oncol, 2014. **16 Suppl 4**: p. iv1-63.
2. Siegel, R.L., K.D. Miller, and A. Jemal, *Cancer statistics, 2015.* CA Cancer J Clin, 2015. **65**(1): p. 5-29.
3. Becker, A.P., et al., *KIAA1549: BRAF Gene Fusion and FGFR1 Hotspot Mutations Are Prognostic Factors in Pilocytic Astrocytomas.* J Neuropathol Exp Neurol, 2015. **74**(7): p. 743-54.
4. Kool, M., et al., *Molecular subgroups of medulloblastoma: an international meta-analysis of transcriptome, genetic aberrations, and clinical data of WNT, SHH, Group 3, and Group 4 medulloblastomas.* Acta Neuropathol, 2012. **123**(4): p. 473-84.
5. Northcott, P.A., et al., *Medulloblastoma comprises four distinct molecular variants.* J Clin Oncol, 2011. **29**(11): p. 1408-14.
6. Wu, G., et al., *Somatic histone H3 alterations in pediatric diffuse intrinsic pontine gliomas and non-brainstem glioblastomas.* Nat Genet, 2012. **44**(3): p. 251-3.
7. Pajtler, K.W., et al., *Molecular Classification of Ependymal Tumors across All CNS Compartments, Histopathological Grades, and Age Groups.* Cancer Cell, 2015. **27**(5): p. 728-43.
8. Louis, D.N., et al., *The 2016 World Health Organization Classification of Tumors of the Central Nervous System: a summary.* Acta Neuropathol, 2016. **131**(6): p. 803-20.
9. Freeman, C.R. and J.P. Farmer, *Pediatric brain stem gliomas: a review.* Int J Radiat Oncol Biol Phys, 1998. **40**(2): p. 265-71.
10. Shankar, G.M., et al., *BRAF alteration status and the histone H3F3A gene K27M mutation segregate spinal cord astrocytoma histology.* Acta Neuropathol, 2016. **131**(1): p. 147-50.
11. Mackay, A., et al., *Integrated Molecular Meta-Analysis of 1,000 Pediatric High-Grade and Diffuse Intrinsic Pontine Glioma.* Cancer Cell, 2017.
12. Cooney, T., et al., *Contemporary survival endpoints: an International Diffuse Intrinsic Pontine Glioma Registry study.* Neuro Oncol, 2017. **19**(9): p. 1279-1280.
13. Chang, L., et al., *Transgene-enforced co-stimulation of CD4+ T cells leads to enhanced and sustained anti-tumor effector functioning.* Cytotherapy, 2007. **9**(8): p. 771-84.
14. Cooper, L.J., et al., *Development and application of CD19-specific T cells for adoptive immunotherapy of B cell malignancies.* Blood Cells Mol Dis, 2004. **33**(1): p. 83-9.
15. Cooper, L.J., et al., *T-cell genetic modification for re-directed tumor recognition.* Cancer Chemother Biol Response Modif, 2005. **22**: p. 293-324.
16. Dudley, M.E., et al., *Adoptive transfer of cloned melanoma-reactive T lymphocytes for the treatment of patients with metastatic melanoma.* J Immunother, 2001. **24**(4): p. 363-73.
17. Eshhar, Z., et al., *Specific activation and targeting of cytotoxic lymphocytes through chimeric single chains consisting of antibody-binding domains and the gamma or zeta subunits of the immunoglobulin and T-cell receptors.* Proc Natl Acad Sci U S A, 1993. **90**(2): p. 720-4.

18. Irving, B.A. and A. Weiss, *The cytoplasmic domain of the T cell receptor zeta chain is sufficient to couple to receptor-associated signal transduction pathways.* Cell, 1991. **64**(5): p. 891-901.
19. Kusmartsev, S. and D.I. Gabrilovich, *Role of immature myeloid cells in mechanisms of immune evasion in cancer.* Cancer Immunol Immunother, 2006. **55**(3): p. 237-45.
20. Park, J.R., et al., *Adoptive transfer of chimeric antigen receptor re-directed cytolytic T lymphocyte clones in patients with neuroblastoma.* Mol Ther, 2007. **15**(4): p. 825-33.
21. Thomas, D.A. and J. Massague, *TGF-beta directly targets cytotoxic T cell functions during tumor evasion of immune surveillance.* Cancer Cell, 2005. **8**(5): p. 369-80.
22. Vence, L., et al., *Circulating tumor antigen-specific regulatory T cells in patients with metastatic melanoma.* Proc Natl Acad Sci U S A, 2007. **104**(52): p. 20884-9.
23. Woo, E.Y., et al., *Regulatory CD4(+)CD25(+) T cells in tumors from patients with early-stage non-small cell lung cancer and late-stage ovarian cancer.* Cancer Res, 2001. **61**(12): p. 4766-72.
24. Kowolik, C.M., et al., *CD28 costimulation provided through a CD19-specific chimeric antigen receptor enhances in vivo persistence and antitumor efficacy of adoptively transferred T cells.* Cancer Res, 2006. **66**(22): p. 10995-1004.
25. Baselga, J., et al., *Phase I studies of anti-epidermal growth factor receptor chimeric antibody C225 alone and in combination with cisplatin.* J Clin Oncol, 2000. **18**(4): p. 904-14.
26. Berger, C., et al., *Safety of targeting ROR1 in primates with chimeric antigen receptor-modified T cells.* Cancer Immunol Res, 2015. **3**(2): p. 206-16.
27. Morello, A., M. Sadelain, and P.S. Adusumilli, *Mesothelin-Targeted CARs: Driving T Cells to Solid Tumors.* Cancer Discov, 2016. **6**(2): p. 133-46.
28. Xiao, X., et al., *Identification and characterization of fully human anti-CD22 monoclonal antibodies.* MAbs, 2009. **1**(3): p. 297-303.
29. Weijtens, M.E., et al., *Single chain Ig/gamma gene-redirected human T lymphocytes produce cytokines, specifically lyse tumor cells, and recycle lytic capacity.* J Immunol, 1996. **157**(2): p. 836-43.
30. Almasbak, H., T. Aarvak, and M.C. Vemuri, *CAR T Cell Therapy: A Game Changer in Cancer Treatment.* J Immunol Res, 2016. **2016**: p. 5474602.
31. Brentjens, R.J., et al., *Genetically targeted T cells eradicate systemic acute lymphoblastic leukemia xenografts.* Clin Cancer Res, 2007. **13**(18 Pt 1): p. 5426-35.
32. Gade, T.P., et al., *Targeted elimination of prostate cancer by genetically directed human T lymphocytes.* Cancer Res, 2005. **65**(19): p. 9080-8.
33. Gill, S., M.V. Maus, and D.L. Porter, *Chimeric antigen receptor T cell therapy: 25years in the making.* Blood Rev, 2016. **30**(3): p. 157-67.
34. Kahlon, K.S., et al., *Specific recognition and killing of glioblastoma multiforme by interleukin 13-zetakine redirected cytolytic T cells.* Cancer Res, 2004. **64**(24): p. 9160-6.
35. Stephan, M.T., et al., *T cell-encoded CD80 and 4-1BBL induce auto- and transcostimulation, resulting in potent tumor rejection.* Nat Med, 2007. **13**(12): p. 1440-9.
36. Teng, M.W., et al., *Immunotherapy of cancer using systemically delivered gene-modified human T lymphocytes.* Hum Gene Ther, 2004. **15**(7): p. 699-708.

37. Westwood, J.A., et al., *Adoptive transfer of T cells modified with a humanized chimeric receptor gene inhibits growth of Lewis-Y-expressing tumors in mice.* Proc Natl Acad Sci U S A, 2005. **102**(52): p. 19051-6.
38. Finney, H.M., et al., *Chimeric receptors providing both primary and costimulatory signaling in T cells from a single gene product.* J Immunol, 1998. **161**(6): p. 2791-7.
39. Friedmann-Morvinski, D., et al., *Redirected primary T cells harboring a chimeric receptor require costimulation for their antigen-specific activation.* Blood, 2005. **105**(8): p. 3087-93.
40. Haynes, N.M., et al., *Single-chain antigen recognition receptors that costimulate potent rejection of established experimental tumors.* Blood, 2002. **100**(9): p. 3155-63.
41. Loskog, A., et al., *Addition of the CD28 signaling domain to chimeric T-cell receptors enhances chimeric T-cell resistance to T regulatory cells.* Leukemia, 2006. **20**(10): p. 1819-28.
42. Moeller, M., et al., *A functional role for CD28 costimulation in tumor recognition by single-chain receptor-modified T cells.* Cancer Gene Ther, 2004. **11**(5): p. 371-9.
43. Pule, M.A., et al., *A chimeric T cell antigen receptor that augments cytokine release and supports clonal expansion of primary human T cells.* Mol Ther, 2005. **12**(5): p. 933-41.
44. Gardner, R.A., et al., *Intent-to-treat leukemia remission by CD19 CAR T cells of defined formulation and dose in children and young adults.* Blood, 2017. **129**(25): p. 3322-3331.
45. Brown, C.E., et al., *Bioactivity and Safety of IL13Ralpha2-Redirected Chimeric Antigen Receptor CD8+ T Cells in Patients with Recurrent Glioblastoma.* Clin Cancer Res, 2015. **21**(18): p. 4062-72.
46. Brown, C.E., et al., *Regression of Glioblastoma after Chimeric Antigen Receptor T-Cell Therapy.* N Engl J Med, 2016. **375**(26): p. 2561-9.
47. Chapoval, A.I., et al., *B7-H3: a costimulatory molecule for T cell activation and IFN-gamma production.* Nat Immunol, 2001. **2**(3): p. 269-74.
48. Castriconi, R., et al., *Identification of 4Ig-B7-H3 as a neuroblastoma-associated molecule that exerts a protective role from an NK cell-mediated lysis.* Proc Natl Acad Sci U S A, 2004. **101**(34): p. 12640-5.
49. Xu, H., et al., *MicroRNA miR-29 modulates expression of immunoinhibitory molecule B7-H3: potential implications for immune based therapy of human solid tumors.* Cancer Res, 2009. **69**(15): p. 6275-81.
50. Benzon, B., et al., *Correlation of B7-H3 with androgen receptor, immune pathways and poor outcome in prostate cancer: an expression-based analysis.* Prostate Cancer Prostatic Dis, 2017. **20**(1): p. 28-35.
51. Inamura, K., et al., *Tumor B7-H3 (CD276) Expression and Survival in Pancreatic Cancer.* J Clin Med, 2018. **7**(7).
52. Xu, Z., et al., *High expression of B7-H3 and CD163 in cancer tissues indicates malignant clinicopathological status and poor prognosis of patients with urothelial cell carcinoma of the bladder.* Oncol Lett, 2018. **15**(5): p. 6519-6526.
53. Zhang, W., et al., *B7-H3 silencing inhibits tumor progression of mantle cell lymphoma and enhances chemosensitivity.* Int J Oncol, 2015. **46**(6): p. 2562-72.
54. Flem-Karlsen, K., et al., *Immunoregulatory protein B7-H3 promotes growth and decreases sensitivity to therapy in metastatic melanoma cells.* Pigment Cell Melanoma Res, 2017. **30**(5): p. 467-476.

55. Gregorio, A., et al., *Small round blue cell tumours: diagnostic and prognostic usefulness of the expression of B7-H3 surface molecule.* Histopathology, 2008. **53**(1): p. 73-80.
56. Zhou, Z., et al., *B7-H3, a potential therapeutic target, is expressed in diffuse intrinsic pontine glioma.* J Neurooncol, 2013. **111**(3): p. 257-64.
57. Majzner, R.G., et al., *CAR T cells targeting B7-H3, a Pan-Cancer Antigen, Demonstrate Potent Preclinical Activity Against Pediatric Solid Tumors and Brain Tumors.* Clin Cancer Res, 2019.
58. Zhang, C., et al., *Large-scale analysis reveals the specific clinical and immune features of B7-H3 in glioma.* Oncoimmunology, 2018. **7**(11): p. e1461304.
59. Baral, A., et al., *B7-H3 and B7-H1 expression in cerebral spinal fluid and tumor tissue correlates with the malignancy grade of glioma patients.* Oncol Lett, 2014. **8**(3): p. 1195-1201.
60. Souweidane, M.M., et al., *Convection-enhanced delivery for diffuse intrinsic pontine glioma: a single-centre, dose-escalation, phase 1 trial.* Lancet Oncol, 2018.
61. Loo, D., et al., *Development of an Fc-enhanced anti-B7-H3 monoclonal antibody with potent antitumor activity.* Clin Cancer Res, 2012. **18**(14): p. 3834-45.
62. Du, H., et al., *Antitumor Responses in the Absence of Toxicity in Solid Tumors by Targeting B7-H3 via Chimeric Antigen Receptor T Cells.* Cancer Cell, 2019. **35**(2): p. 221-237 e8.
63. Majzner, R.G., et al., *CAR T Cells Targeting B7-H3, a Pan-Cancer Antigen, Demonstrate Potent Preclinical Activity Against Pediatric Solid Tumors and Brain Tumors.* Clin Cancer Res, 2019.
64. Picarda, E., K.C. Ohaegbulam, and X. Zang, *Molecular Pathways: Targeting B7-H3 (CD276) for Human Cancer Immunotherapy.* Clin Cancer Res, 2016. **22**(14): p. 3425-3431.
65. Fry, T.J., et al., *CD22-targeted CAR T cells induce remission in B-ALL that is naive or resistant to CD19-targeted CAR immunotherapy.* Nat Med, 2018. **24**(1): p. 20-28.
66. Walker, A.J., et al., *Tumor Antigen and Receptor Densities Regulate Efficacy of a Chimeric Antigen Receptor Targeting Anaplastic Lymphoma Kinase.* Mol Ther, 2017. **25**(9): p. 2189-2201.
67. Kramer, K., et al., *Compartmental intrathecal radioimmunotherapy: results for treatment for metastatic CNS neuroblastoma.* J Neurooncol, 2010. **97**(3): p. 409-18.
68. Souweidane, M.M., et al., *Convection-enhanced delivery for diffuse intrinsic pontine glioma: a single-centre, dose-escalation, phase 1 trial.* Lancet Oncol, 2018. **19**(8): p. 1040-1050.
69. Powderly, J., et al., *Interim results of an ongoing Phase I, dose escalation study of MGA271 (Fc-optimized humanized anti-B7-H3 monoclonal antibody) in patients with refractory B7-H3-expressing neoplasms or neoplasms whose vasculature expresses B7-H3.* Journal of ImmunoTherapy of Cancer, 2015. **3**(8).
70. Wang, X., et al., *A transgene-encoded cell surface polypeptide for selection, in vivo tracking, and ablation of engineered cells.* Blood, 2011. **118**(5): p. 1255-63.
71. Ercikan-Abali, E.A., et al., *Active site-directed double mutants of dihydrofolate reductase.* Cancer Res, 1996. **56**(18): p. 4142-5.
72. Jonnalagadda, M., et al., *Efficient selection of genetically modified human T cells using methotrexate-resistant human dihydrofolate reductase.* Gene Ther, 2013. **20**(8): p. 853-60.

73. Frank, G.M., et al., *Early CD4(+) T cell help prevents partial CD8(+) T cell exhaustion and promotes maintenance of Herpes Simplex Virus 1 latency.* J Immunol, 2010. **184**(1): p. 277-86.
74. Hayes, R.L., *The cellular immunotherapy of primary brain tumors.* Rev Neurol (Paris), 1992. **148**(6-7): p. 454-66.
75. Ingram, M., et al., *Salvage immunotherapy of malignant glioma.* Arch Surg, 1987. **122**(12): p. 1483-6.
76. Jacobs, S.K., et al., *Interleukin-2 and lymphokine activated killer (LAK) cells in the treatment of malignant glioma: clinical and experimental studies.* Neurol Res, 1986. **8**(2): p. 81-7.
77. Jeffes, E.W., 3rd, et al., *Therapy of recurrent high grade gliomas with surgery, and autologous mitogen activated IL-2 stimulated killer (MAK) lymphocytes: I. Enhancement of MAK lytic activity and cytokine production by PHA and clinical use of PHA.* J Neurooncol, 1993. **15**(2): p. 141-55.
78. Kruse, C.A., et al., *Treatment of recurrent glioma with intracavitary alloreactive cytotoxic T lymphocytes and interleukin-2.* Cancer Immunol Immunother, 1997. **45**(2): p. 77-87.
79. Merchant, R.E., et al., *Treatment of recurrent malignant glioma by repeated intracerebral injections of human recombinant interleukin-2 alone or in combination with systemic interferon-alpha. Results of a phase I clinical trial.* J Neurooncol, 1992. **12**(1): p. 75-83.
80. Nakanishi, Y., et al., *CD8(+) T lymphocyte mobilization to virus-infected tissue requires CD4(+) T-cell help.* Nature, 2009. **462**(7272): p. 510-3.
81. Novy, P., et al., *CD4 T cells are required for CD8 T cell survival during both primary and memory recall responses.* J Immunol, 2007. **179**(12): p. 8243-51.
82. Sankhla, S.K., J.S. Nadkarni, and S.N. Bhagwati, *Adoptive immunotherapy using lymphokine-activated killer (LAK) cells and interleukin-2 for recurrent malignant primary brain tumors.* J Neurooncol, 1996. **27**(2): p. 133-40.
83. Yoshida, S., et al., *[Adoptive immunotherapy in patients with malignant glioma].* Gan To Kagaku Ryoho, 1987. **14**(6 Pt 1): p. 1930-2.
84. Mahdi, J., et al., *Tumor inflammation-associated neurotoxicity.* Nat Med, 2023. **29**(4): p. 803-810.
85. Collins, P.Y., et al., *Grand challenges in global mental health.* Nature, 2011. **475**(7354): p. 27-30.
86. Luger, T.J., et al., *Intracerebroventricular and intrathecal injectate spread in rats.* Eur J Anaesthesiol, 2005. **22**(3): p. 236-9.
87. Picard, N.A. and C.A. Zanardi, *Brain motion and volume transmission: Keeping the interstice flowing.* Med Hypotheses, 2015. **85**(1): p. 41-4.
88. Vitanza, N.A., et al., *Locoregional CAR T cells for children with CNS tumors: Clinical procedure and catheter safety.* Neoplasia, 2023. **36**: p. 100870.
89. Alizadeh, D., et al., *Induction of anti-glioma natural killer cell response following multiple low-dose intracerebral CpG therapy.* Clin Cancer Res, 2010. **16**(13): p. 3399-408.
90. Matsuzawa, J., et al., *Age-related volumetric changes of brain gray and white matter in healthy infants and children.* Cereb Cortex, 2001. **11**(4): p. 335-42.

91. Xenos, C., S. Sgouros, and K. Natarajan, *Ventricular volume change in childhood.* J Neurosurg, 2002. **97**(3): p. 584-90.
92. Trippett, T.M., et al., *Phase I and pharmacokinetic study of cetuximab and irinotecan in children with refractory solid tumors: a study of the pediatric oncology experimental therapeutic investigators' consortium.* J Clin Oncol, 2009. **27**(30): p. 5102-8.
93. Okada, H., et al., *Immunotherapy response assessment in neuro-oncology: a report of the RANO working group.* Lancet Oncol, 2015. **16**(15): p. e534-e542.

14 APPENDICES

Appendix 1a – sponsor Signature Page

Appendix 1b - Principal Investigator Signature Page

Appendix 2 – Schedule of Procedures

Appendix 3 – Performance Status Scales

Appendix 4 – Neurologic Toxicity Grading System

Appendix 5 –Refractory and Recurrent Disease Categorization

Appendix 6 – Highly Effective Contraception

APPENDIX 1A – SPONSOR SIGNATURE PAGE

Protocol Title: Phase 1 Study of B7-H3-Specific CAR T Cell Locoregional Immunotherapy for Diffuse Intrinsic Pontine Glioma, Diffuse Midline Glioma, and Recurrent or Refractory Pediatric Central Nervous System Tumors

Sponsor Acknowledgement:

As the Sponsor representative, I confirm that Seattle Children's Therapeutics will comply with all Sponsor obligations as detailed in all applicable regulations and guidelines. I will ensure that the investigator is informed of all relevant information that becomes available during the conduct of this study.

Colleen Annesley, MD Date
Medical Director, Seattle Children's Therapeutics

APPENDIX 1B – PRINCIPAL INVESTIGATOR SIGNATURE PAGE

Protocol Title: Phase 1 Study of B7-H3-Specific CAR T Cell Locoregional Immunotherapy for Diffuse Intrinsic Pontine Glioma, Diffuse Midline Glioma, and Recurrent or Refractory Pediatric Central Nervous System Tumors

Principal Investigator Acknowledgement:

I have read the Protocol, including all appendices, and I agree to conduct the study as detailed in this protocol and in compliance with the Declaration of Helsinki, Good Clinical Practices (GCP) and all applicable regulatory requirements and guidelines.

Principal Investigator Date

Seattle Children's® Therapeutics

APPENDIX 2 – SCHEDULE OF PROCEDURES

Appendix 2, Table 1a Schedule of Procedures, Arms A and B *Screening through End of Therapy/Early Discontinuation*

Appendix 2, Table 1a Schedule of Procedures, Arms A and B, Screening through End of Therapy/Early Discontinuation	Screening	Apheresis	Course 1							Course 2							Course 3 and beyond					End of Therapy / Early Discontinuation	28 Day Follow-up
			Week 1		Week 2		Week 3		Week 4	Week 1		Week 2		Week 3		Week 4	Week 1	Week 2	Week 3	Odd Courses (3, 5, etc), Week 4	Even Courses (4, 6, etc), Week 4		
			Infusion	Post-infusion	Infusion	Post-infusion	Infusion	Post-infusion		Infusion	Post-infusion	Infusion	Post-infusion	Infusion	Post-infusion		Infusion	Infusion	Infusion				
**Procedure Window* →** (unless otherwise noted)		e	f	h	f, g	h	f, g	h	i	f	h	f, g	h	f, g	h	i	f	f, g	f, g	i	i	l	m
**Informed consent/assent**	X [a]																						
**Eligibility determination**	X [a]	X	X		X		X			X		X		X			X	X	X				
**Demography**	X [a]																						
**Medical history**	X [a]		X [1]																				
**Disease history**	X [a]		X [1]																				
**Treatment history**	X [a]		X [1]																				
**Performance status [2]**	X [b]		X		X		X		X	X		X		X		X	X	X	X	X	X	X	
**Physical exam, vital signs [3], weight**	X [b]	X	X	X	X	X	X	X	X	X	X	X	X	X	X	X	X	X	X	X	X	X	
**Neurologic exam**	X [b]		X	X	X	X	X	X	X	X	X	X	X	X	X	X	X	X	X	X	X	X	
**Height**	X [b]																						

Seattle Children's Therapeutics

Appendix 2, Table 1a Schedule of Procedures, Arms A and B, Screening through End of Therapy/Early Discontinuation	Screening	Apheresis	Course 1							Course 2							Course 3 and beyond					End of Therapy / Early Discontinuation	28 Day Follow-up
			Week 1		Week 2		Week 3			Week 1		Week 2		Week 3			Week 1	Week 2	Week 3				
			Infusion	Post-infusion	Infusion	Post-infusion	Infusion	Post-infusion	Week 4	Infusion	Post-infusion	Infusion	Post-infusion	Infusion	Post-infusion	Week 4	Infusion	Infusion	Infusion	Odd Courses (3, 5, etc), Week 4	Even Courses (4, 6, etc), Week 4		
**Procedure Window* →** (unless otherwise noted)		e	f	h	f, g	h	f, g	h	i	f	h	f, g	h	f, g	h	i	f	f, g	f, g	i	i	l	m
**Pulse oximetry**	X[b]		X		X		X			X		X		X			X	X	X				
**Chemistry** [5]	X[b]	X[n]	X	X	X	X	X	X	X	X	X	X	X	X	X	X	X	X	X	X	X	X	
**Hematology** [6]	X[b]	X	X	X	X	X	X	X	X	X	X	X	X	X	X	X	X	X	X	X	X	X	
**Pregnancy test** [4]	X[b]		X							X							X						
**CRS labs and evaluation**[7]			Must be obtained daily during periods of grade 2-4 CRS																				
**Virology** [8]	X[d]																						
**CSF: Arm A** [9]	X[c]								X							X					X	X	
**Correlative Sciences: CSF Arm A** [11]									X							X					X	X	
**CSF: Arm B** [9]	X[c]		X	X	CX		X	X	X	X	X	X		X	X	X	X		X		X	X	
**Correlative Sciences: CSF Arm B** [11]			X	X	X		X	X	X	X	X	X		X	X	X	X		X		X	X	
**Correlative Sciences: PB** [12]		X	X	X	X				X	X	X	X				X			X	X	X	X	
**Correlative Sciences: other** [14]																							
**MRI brain & spine**	X[c]		X[j]													X[k]					X[k]	X[k]	
**Apheresis**		X																					

Appendix 2, Table 1a Schedule of Procedures, Arms A and B, Screening through End of Therapy/Early Discontinuation	Screening	Apheresis	Course 1							Course 2							Course 3 and beyond					End of Therapy / Early Discontinuation	28 Day Follow-up
			Week 1		Week 2		Week 3			Week 1		Week 2		Week 3			Week 1	Week 2	Week 3				
			Infusion	Post-infusion	Infusion	Post-infusion	Infusion	Post-infusion	Week 4	Infusion	Post-infusion	Infusion	Post-infusion	Infusion	Post-infusion	Week 4	Infusion	Infusion	Infusion	Odd Courses (3, 5, etc), Week 4	Even Courses (4, 6, etc), Week 4		
**Procedure Window*** → (unless otherwise noted)		e	f	h	f, g	h	f, g	h	i	f	h	f, g	h	f, g	h	i	f	f, g	f, g	i	i	l	m
**CAR T cell infusion Arm A & Arm B**			X		X		X			X		X		X			X	X	X				
**Adverse events** [13]			Continuous through Day +28 [p]																				
**Concomitant Medications**		X	Continuous through Day+28 [p]																				

* Treatment Plan delays may be allowed, see Section 5.1.6.

a Evaluations must be performed prior to study enrollment, but have no specific procedure window

b Evaluations must be performed within 7 days prior to study enrollment. Results of standard-of-care tests or examinations performed prior to obtaining informed consent may be used

c Evaluations must be performed within 28 days prior to study enrollment. Results of standard-of-care tests or examinations performed prior to obtaining informed consent may be used

d Evaluations must be performed within 3 months prior to study enrollment. Results of standard-of-care tests or examinations performed prior to obtaining informed consent may be used

e Evaluations must be performed within 2 days prior to any apheresis (See Section 5.3 for repeat apheresis criteria)

f Evaluations must be performed within 2 days prior to CAR T cell infusion unless the infusion is omitted in which case evaluations are optional (See Section 5.1.6)

g Infusions must be performed ≥ 7 days after the previous infusion

h Evaluations must be performed 2 to 5 days after CAR T cell infusion unless the infusion omitted in which case evaluations are optional (See Section 5.1.6). Post-infusion evaluations, except Correlative Sciences evaluations, may be used for infusion or Week 4 evaluations if performed within the required window. Distinct Correlative Sciences evaluations must be completed at every timepoint

i Evaluations may be performed at any point during Week 4 +/- 3 days. Evaluations are optional for odd courses beyond Course 2

j Required prior to Course 1 Week 1 if bridging therapy received AND >28 days from prior scans, or if clinically indicated

k If clinically indicated Disease Evaluation CSF and MRI brain and spine should occur prior to receiving the next course. Disease Evaluations are no longer required if progressive disease was confirmed in a prior evaluation

l End of Therapy/Early Discontinuation Evaluations must be performed within 14 days following the final CAR T cell infusion, EXCEPT MRI brain and spine which must be performed within 28 days following the final CAR T cell infusion. Any evaluations completed for a timepoint following the final T cell infusion may be used as End of Therapy/Early Discontinuation evaluations if performed within the indicated window. If greater than 10 days have elapsed since the subject's last dose before it is determined that subject will no longer receive protocol therapy, assessments may be performed within 28 days of the subject's last dose. Assessments may be obtained from local provider or treating oncologist

m Evaluations must be performed 28 (+ 7) days post final CAR T cell infusion. The purpose of the 28 day follow-up visit is to ensure adverse event and concomitant medication information is collected per the time period specified by the protocol. Visit may be done in person, by the subject's LCI, or via phone

n Required if >7 days from screening, or clinically indicated

p SAE collection must be ongoing until Day +30 post the final CAR T cell infusion

1 To include medical, disease and treatment history information from time of apheresis to Course 1, Week 1 CAR T cell infusion

2 Performance status will be assessed utilizing Lansky (for subjects < 16 years of age) or Karnofsky (for subjects ≥ 16 years of age). Subjects who are unable to walk because of paralysis, but who are up in a wheelchair, will be considered ambulatory for purposes of assessing performance status

3 Vital signs must include pulse, respiratory rate, blood pressure and temperature

4 Female subjects of child-bearing potential only

5 Chemistry must include serum sodium, potassium, chloride, bicarbonate, blood urea nitrogen (BUN), creatinine, alanine aminotransferase (ALT), total bilirubin, conjugated bilirubin, and C reactive protein (CRP)

6 Hematology must include complete blood count (CBC) including hemoglobin, hematocrit, red blood cell count, white blood cell count, white blood cell differential, and platelet count

7 CRS labs and evaluation must be obtained daily during periods of grade 2-4 cytokine release syndrome. CRS labs must include CRP, LDH, PT/PTT, d-dimer, fibrinogen and absolute lymphocyte count. CRS evaluation must include temperature, heart rate and blood pressure

8 Virology must include human immunodeficiency virus (HIV) antigen and antibody, Hepatitis B surface antigen, and Hepatitis C antibody testing; if Hepatitis C antibody testing is positive, quantitative PCR will be performed

9 CSF samples may be obtained via CNS catheter; testing at all time points must include cell count, glucose, protein and cytology, except at screening where only cell count and cytology are required. If CSF collection via catheter is not feasible the sample is not required

11 Refer to study specific lab manual for collection and shipping requirements of all Correlative Sciences samples. In addition to samples required per the Schedule of Procedures, in the event of neurologic toxicity CSF must be sent to CSL for cytokine analysis and presence of CAR T cells (Section 7.16 for guidance). Also, subjects who receive cetuximab for ablation of T cells (Section 6.5) will require additional CSF to be sent to CSL for determination of CAR T cell persistence: Arm A subjects will have CSF for Correlative Sciences collected prior to the initial dose of cetuximab and again at Day 7 following the initial dose of cetuximab. Arm B subjects will have CSF for Correlative Sciences collected prior to the initial dose of cetuximab and again on days 1, 3, 7, 10, 14, and 28 days following the initial dose of cetuximab

12 Refer to study specific lab manual for collection and shipping requirements of all Correlative Sciences samples. Pre-IP administration RCL testing will be performed on the pre-infusion sample. If testing is not successful, the pre-apheresis sample will be tested. In addition to samples required per the Schedule of Procedures, in the event of systemic toxicity related to CAR T cells or neurologic toxicity (Section 7.16), peripheral blood may be requested to be sent to the CSL up to once per day. All subjects who

receive cetuximab for ablation of T cells will require PB Correlative Sciences samples be collected prior to initial dose of cetuximab and at days 1, 3, 7, 10, 14 and 28 after initial dose of cetuximab for determination of T cell persistence

13 Baseline for assessing adverse events is established by the evaluations performed within 2 days prior to the Course 1, Week 1 CAR T cell infusion

14 "Other" Correlative Sciences samples are not a regularly scheduled protocol procedure, however if archival tumor tissue, tumor biopsy/resection or other tissue/material is collected for clinical purposes samples may be sent to the CSL for evaluation of marker expression, immune modulation and/or presence of CAR T cells (see Section 7.16 for further guidance); refer to lab manual for requirements of all Correlative Sciences samples

Seattle Children's Therapeutics

Appendix 2, Table 1b Schedule of Procedures, Arm C *Screening through End of Therapy/Early Discontinuation*

Appendix 2, Table 1b Schedule of Procedures, Arm C, Screening through End of Therapy/Early Discontinuation	Screening	Apheresis	Course 1						Course 2						Course 3 and beyond				End of Therapy / Early Discontinuation	28 Day Follow-up
			Week 1			Week 3			Week 1			Week 3			Week 1	Week 3				
			Infusion	Post-infusion	Week 2	Infusion	Post-infusion	Week 4	Infusion	Post-infusion	Week 2	Infusion	Post-infusion	Week 4	Infusion	Infusion	Odd Courses (3, 5, etc) Week 4	Even Courses (4, 6, etc.) Week 4		
Procedure Window* → (unless otherwise noted)		e	f	h	z	f, g	h	i	f	h	z	f, g	h	i	f	f, g	i	i	l	m
**Informed consent/assent**	X [a]																			
**Eligibility determination**	X [a]	X	X			X			X			X			X	X				
**Demography**	X [a]																			
**Medical history**	X [a]		X [1]																	
**Disease history**	X [a]		X [1]																	
**Treatment history**	X [a]		X [1]																	
**Performance status** [2]	X [b]		X		X	X		X	X		X	X		X	X	X	X	X	X	
**Physical exam, vital signs** [3]**, weight**	X [b]	X	X	X	X	X	X	X	X	X	X	X	X	X	X	X	X	X	X	
**Neurologic exam**	X [b]		X	X	X	X	X	X	X	X	X	X	X	X	X	X	X	X	X	
**Height**	X [b]																			
**Pulse oximetry**	X [b]		X			X			X			X			X	X				
**Chemistry** [5]	X [b]	X [o]	X	X		X	X	X	X	X		X	X	X	X	X	X	X	X	
**Hematology** [6]	X [b]	X	X	X		X	X	X	X	X		X	X	X	X	X	X	X	X	
**Pregnancy test** [4]	X [b]		X						X						X					

Appendix 2, Table 1b Schedule of Procedures, Arm C, Screening through End of Therapy/Early Discontinuation	Screening	Apheresis	Course 1						Course 2						Course 3 and beyond				End of Therapy / Early Discontinuation	28 Day Follow-up
			Week 1		Week 2	Week 3		Week 4	Week 1		Week 2	Week 3		Week 4	Week 1	Week 3	Odd Courses (3, 5, etc) Week 4	Even Courses (4, 6, etc.) Week 4		
			Infusion	Post-infusion		Infusion	Post-infusion		Infusion	Post-infusion		Infusion	Post-infusion		Infusion	Infusion				
**Procedure Window*** → (unless otherwise noted)		e	f	h	z	f, g	h	i	f	h	z	f, g	h	i	f	f, g	i	i	l	m
**CRS labs and evaluation**[7]			Must be obtained daily during periods of grade 2-4 CRS																	
**Virology** [8]	X[d]																			
**CSF** [9]	X[c]		X	X		X	X	X	X	X		X	X	X	X				X[k]	
**Correlative Sciences: CSF** [10]			X	X		X	X	X	X	X		X	X	X	X				X	
**Correlative Sciences: PB** [11]		X	X[n]	X				X	X	X				X		X	X	X	X	
**Correlative Sciences: other**[13]																				
**MRI brain & spine**	X[c]		X[j]											X[k]				X[k]	X[k]	
**Apheresis**		X																		
**CAR T cell infusion Arm C**			X			X			X			X			X	X				
**Adverse events** [13]			Continuous through Day +28[p]																	
**Concomitant Medications**		X	Continuous through Day+28[p]																	

* Treatment Plan delays may be allowed, see Section 5.1.6.

a Evaluations must be performed prior to study enrollment, but have no specific procedure window

b Evaluations must be performed within 7 days prior to study enrollment. Results of standard-of-care tests or examinations performed prior to obtaining informed consent may be used.

c Evaluations must be performed within 28 days prior to study enrollment. Results of standard-of-care tests or examinations performed prior to obtaining informed consent may be used.

d Evaluations must be performed within 3 months prior to study enrollment. Results of standard-of-care tests or examinations performed prior to obtaining informed consent may be used.

e Evaluations must be performed within 2 days prior to any apheresis (See Section 5.3 for repeat apheresis criteria).

f Evaluations must be performed within 2 days prior to CAR T cell infusion unless the infusion is omitted in which case evaluations are optional (See Section 5.1.6).

g Infusions must be performed ≥ 7 days after the previous infusion.

h Evaluations must be performed 2 to 5 days after CAR T cell infusion unless the infusion is omitted in which case evaluations are optional (See Section 5.1.6). Post-infusion evaluations, except Correlative Sciences evaluations, may be used for infusion or Week 4 evaluations if performed within the required window. Correlative Sciences evaluations must be completed at every timepoint.

i Evaluations may be performed at any point during Week 4 +/- 3 days. Evaluations are optional for odd courses beyond Course 2.

j Required prior to Course 1 Week 1 if bridging therapy received AND >28 days from prior scans, or if clinically indicated.

k If clinically indicated Disease evaluation CSF and MRI brain and spine should occur prior to receiving the next course. Disease Evaluations are no longer required if progressive disease was confirmed in a prior evaluation.

l End of Therapy/Early Discontinuation Evaluations must be performed within 14 days following the final CAR T cell infusion EXCEPT MRI brain and spine which must be performed within 28 days following the final CAR T cell infusion. Any evaluations completed for a timepoint following the final T cell infusion may be used as End of Therapy/Early Discontinuation evaluations if performed within the indicated window. If greater than 10 days have elapsed since the subject's last dose before it is determined that subject will no longer receive protocol therapy, assessments may be performed within 28 days of the subject's last dose. Assessments may be obtained from local provider or treating oncologist.

m Evaluations must be performed 28 (+ 7) days post final CAR T cell infusion. The 28 day follow-up visit purpose is to ensure adverse event and concomitant medication information is collected per the time period specified by the protocol. Visit may be done in person, by the subject's LCI, or via phone.

n Pre-IP administration RCL testing will be performed on the pre-infusion sample. If testing is not successful, the pre-apheresis sample will be tested.

o Required if >7 days from screening, or clinically indicated.

p SAE collection must be ongoing until Day +30 post the final CAR T cell infusion.

z Evaluations must be performed 8 to 14 days after Week 1 CAR T cell infusion. Week 2 evaluations may be used for week 3 pre-infusion evaluations if performed within 2 days of the week 3 infusion.

1 To include medical, disease and treatment history information from time of apheresis to Course 1, Week 1 CAR T cell infusion.

2 Performance status will be assessed utilizing Lansky (for subjects < 16 years of age) or Karnofsky (for subjects ≥ 16 years of age). Subjects who are unable to walk because of paralysis, but who are up in a wheelchair, will be considered ambulatory for purposes of assessing performance status.

3 Vital signs must include pulse, respiratory rate, blood pressure and temperature.

4 Female subjects of child-bearing potential only.

5 Chemistry must include serum sodium, potassium, chloride, bicarbonate, blood urea nitrogen (BUN), creatinine, alanine aminotransferase (ALT), total bilirubin, conjugated bilirubin, and C reactive protein (CRP).

6 Hematology must include complete blood count (CBC) including hemoglobin, hematocrit, red blood cell count, white blood cell count, white blood cell differential, and platelet count.

7 CRS labs and evaluation must be obtained daily during periods of grade 2-4 CRS. CRS labs must include CRP, LDH, PT/PTT, ferritin, d-dimer, fibrinogen and absolute lymphocyte count. CRS evaluation must include temperature, heart rate and blood pressure.

8 Virology must include human immunodeficiency virus (HIV) antigen and antibody, Hepatitis B surface antigen, and Hepatitis C antibody testing; if Hepatitis C antibody testing is positive, quantitative polymerase chain reaction (PCR) will be performed.

9 CSF may be obtained via CNS catheter; testing at all time points must include cell count, glucose, protein, and cytology except at screening when only cell count and cytology are required. If CSF collection via catheter is not feasible the sample is not required.

10 Refer to study specific lab manual for collection and shipping requirements of all Correlative Sciences samples. In addition to samples required per the Schedule of Procedures, in the event of neurologic toxicity CSF must be sent to CSL for cytokine analysis and presence of CAR T cells (Section 7.16 for guidance). Also, subjects who receive cetuximab for ablation of T cells (Section 6.5) will require additional CSF to be sent to CSL for determination of CAR T cell persistence: Arm c subjects will have CSF for Correlative Sciences collected prior to the initial dose of cetuximab and again on days 1, 3, 7, 10, 14, and 28 days following the initial dose of cetuximab.

11 Refer to study specific lab manual for collection and shipping requirements of all Correlative Sciences samples. In addition to samples required per the Schedule of Procedures, in the event of systemic toxicity related to CAR T cells or neurologic toxicity (Section 7.16), peripheral blood may be requested to be sent to the CSL up to once per day. All subjects who receive cetuximab for ablation of T cells will require PB Correlative Sciences samples be collected prior to initial dose of cetuximab and at days 1, 3, 7, 10, 14 and 28 after initial dose of cetuximab for determination of T cell persistence.

12 Baseline for assessing adverse events is established by the evaluations performed within 2 days prior to the Course 1, Week 1 CAR T cell infusion.

13 “Other” Correlative Sciences samples are not a regularly scheduled protocol procedure, however if archival tumor tissue, tumor biopsy/resection or other tissue/material is collected for clinical purposes samples may be sent to the CSL for evaluation of marker expression, immune modulation and/or presence of CAR T cells (see Section 7.16 for further guidance); refer to lab manual for requirements of all Correlative Sciences samples.

Seattle Children's® Therapeutics

Appendix 2, Table 2 Schedule of Procedures, All Arms ***Long-Term Follow-Up, Month 3 through Year 5***

		**Timepoint: months following final CAR T cell infusion**											
		**Month 3**	**Month 6**	**Month 9**	**Month 12 (Year 1)**	**Month 18**	**Month 24 (Year 2)**	**Month 30**	**Month 36 (Year 3)**	**Month 42**	**Month 48 (Year 4)**	**Month 54**	**Month 60 (Year 5)**
	**Procedure Window →**	± 3 months											
**Medical history** [1, 2]			X		X		X		X		X		X
**Physical exam** [1]			X		X		X		X		X		X
**CSF**[1, 3]		X	X	X	X								
**Correlative Sciences: PB**	**CAR T cell persistence** [4]		X		X	X	X	X	X	X	X	X	X
	**Replication-competent lentivirus (RCL)** [7]	X	X		X								
**MRI brain & spine** [1, 5]		X	X	X	X								
**Adverse events** [6]		Continuous											

1 If performed for clinical indication. May be obtained from local provider or treating oncologist

2 Medical events relevant to subject's disease and treatment, inclusive of infectious disease. If relevant, records pertaining to disease response and/or relapse will be collected.

3 Testing at all time points must include cell count and cytology. Any additional material obtained for clinical purposes may be sent to CSL for research testing. Disease Evaluations are no longer required if progressive disease was confirmed in a prior evaluation.

4 Timepoints only applicable if ongoing T cell persistence in peripheral blood at the end of protocol therapy/discontinuation visit. CAR T cell persistence testing will be discontinued after a negative result.

5 MRI of the spine for subjects with previous spinal disease, positive CSF cytology, or cause for clinical concern. Disease Evaluations are no longer required if progressive disease was confirmed in a prior evaluation.

6 The following are Delayed Related AEs of interest that must be reported to the Sponsor: development of a new malignancy, or neurologic, rheumatologic, autoimmune, or hematologic disorder

7 RCL testing will be discontinued if no positive results during the first year. Further time points may be determined by sponsor after review with FDA if RCL testing is positive at any time during the first year.

Appendix 2, Table 3 Schedule of Procedures, All Arms *Long-Term Follow-up, Years 6 through 15*

	Timepoint: years following final CAR T cell infusion									
	Year 6	Year 7	Year 8	Year 9	Year 10	Year 11	Year 12	Year 13	Year 14	Year 15
**Procedure Window →**	± 2 months									
**Medical history**[1, 2]	X	X	X	X	X	X	X	X	X	X
**Physical exam**[1, 5]	X	X	X	X	X	X	X	X	X	X
**Correlative Sciences: PB CAR T cell persistence**[1,3]	X	X	X	X	X	X	X	X	X	X
**Adverse events**[4]	Continuous									

1 May be obtained from local provider or treating oncologist
2 Interim medical history relevant to subject's disease, treatment and status; this information may be obtained by phone, unless subject has evidence of ongoing CAR T cell persistence or RCL, in which case subject must be seen in person
3 Timepoints only applicable if ongoing T cell persistence in peripheral blood at the 60-month visit. CAR T cell persistence testing will be discontinued after a negative result
4 The following are Delayed Related AEs of interest that must be reported to the Sponsor: development of a new malignancy, or neurologic, rheumatologic, autoimmune, or hematologic disorder
5 Only required for subjects with evidence of ongoing CAR T cell persistence or RCL.

APPENDIX 3 – PERFORMANCE STATUS SCALES

**Performance Status Criteria**<br>Karnofsky and Lansky performance scores are intended to be multiples of 10			
**Karnofsky**<br>subjects ≥ 16 years of age		**Lansky**<br>subjects < 16 years of age	
Score	***Description***	***Score***	***Description***
100	Normal, no complaints, no evidence of disease	100	Fully active, normal
90	Able to carry on normal activity, minor signs or symptoms of disease	90	Minor restrictions in physically strenuous activity
80	Normal activity with effort; some signs or symptoms of disease	80	Active, but tires more quickly
70	Cares for self, unable to carry on normal activity or do active work	70	Both greater restriction of and less time spent in play activity
60	Required occasional assistance, but is able to care for most of his/her needs	60	Up and around, but minimal active play; keeps busy with quieter activities
50	Requires considerable assistance and frequent medical care	50	Gets dressed, but lies around much of the day; no active play, able to participate in all quiet play and activities
40	Disabled, requires special care and assistance	40	Mostly in bed, participates in quiet activities
30	Severely disabled, hospitalization indicated. Death not imminent	30	In bed, needs assistance even for quiet play
20	Very sick, hospitalization indicated. Death not imminent	20	Often sleeping, play entirely limited to very passive activities
10	Moribund, fatal processes progressing rapidly	10	No play, does not get out of bed

APPENDIX 4 – NEUROLOGIC TOXICITY GRADING SYSTEM

Individual neurologic symptoms should be graded using CTCAE v5, however cumulative neurologic toxicity will be graded according to the Neurologic Toxicity Grading System.

Grade of Toxicity	Toxicity Description
**0**	Normal or no change from baseline examination at the start of therapy
1	Mild lethargy and/or irritability, and/or headache, visual, motor, or sensory symptoms without change in neurological exam
2	Moderate lethargy, disorientation, psychosis lasting less than 48 hrs, or mild increase in preexisting neurological deficit
3	Greater than 48 hours of severe lethargy, responsive to verbal stimuli, disorientation or psychosis lasting greater than 48 hours, moderate increase in preexisting neurological deficit or the onset of new neurological signs, greater than 2 seizures in 24 hours
4	Coma, unresponsive to verbal stimuli, increasing neurological deficit above grade 3, evidence of herniation, development of uncontrolled seizures, intracerebral hemorrhage
5	Death

APPENDIX 5 –REFRACTORY AND RECURRENT DISEASE CATEGORIZATION

- DIPG and DMG (WHO grade IV)
- Embryonal Tumor [including, but not limited to, medulloblastoma, atypical teratoid rhabdoid tumor (ATRT), pineoblastoma, embryonal tumor with multilayered rosettes (ETMR), and embryonal tumor NOS] that was refractory to standard first-line therapy or has recurred after standard first-line therapy.
- Low-grade glioma [including but not limited to pilocytic astrocytoma (WHO grade I) and diffuse astrocytoma (WHO grade II)] that was refractory or recurred after standard first-line therapy and received a standard second-line therapy to which it was refractory, or after which developed another recurrence.
- High-grade glioma [including, but not limited to, anaplastic astrocytoma (WHO grade III), glioblastoma (WHO grade IV)] that was refractory to standard first-line therapy or has recurred after standard first-line therapy.
- Ependymoma that was refractory to standard first-line therapy or has recurred after standard first-line therapy.
- Germ cell tumors (including but not limited to germinoma and nongerminomatous germ cell tumor) that was refractory to standard first-line therapy or has recurred after standard first-line therapy.
- Other WHO grade III or IV CNS tumor (including, but not limited to, choroid plexus carcinomas) that was refractory to standard first-line therapy or has recurred after standard first-line therapy.
- Other WHO grade I or II CNS tumor (including, but not limited to, neuroglial tumors) that was refractory or recurred after standard first-line therapy and received a standard second-line therapy to which it was refractory, or after which developed another recurrence.

APPENDIX 6 – HIGHLY EFFECTIVE CONTRACEPTION

The following methods of birth control are considered highly effective in preventing pregnancy:

- Total abstinence, when this is in line with the subject's preferred and usual lifestyle. Periodic abstinence like calendar, ovulation, symptothermal, post-ovulation methods, and withdrawal are not acceptable methods of contraception.
- Female sterilization, when the female subject has been surgically sterilized at least 6 weeks prior to enrollment (bilateral oophorectomy or bilateral salpingectomy).
- Male sterilization: the male subject, or female subject's sole sexual partner has been surgically sterilized at least 6 weeks be for enrollment (vasectomy). If the partner of a female subject, appropriate documentation of sterilization should be provided.
- Male subjects: use of a condom during intercourse. In addition, it is advised that the subject's female partner use an additional highly effective method of contraception (hormonal contraception, IUD, etc.).
- Female subjects of childbearing potential: use of a combination of any two of the following:
  - Use of oral, injected or implanted hormonal methods of contraception or other forms of hormonal contraception that have comparable efficacy (failure rate < 1%), for example hormone vaginal ring or transdermal hormone contraception. If oral contraception, subject should be on a stable dose of the same medication for ≥ 3 months prior to enrollment;
  - Placement of an intrauterine device (IUD) or intrauterine system (IUS);
  - Use of an occlusive cap (diaphragm or cervical/vault cap) by a female subject, or a condom by a female subject's male partner, combined with a spermicidal foam/gel/film/cream/vaginal suppository.

BrainChild-03 Protocol Version History

Document	Document Version/Version Date (vN.N/YYYY.MM.DD)	SC IRB Approval Date (YYYY.MM.DD)	Key Changes
Original Protocol submitted to FDA	V0.1/2019.04.24	N/A	N/A - Original
Original Protocol submitted to SRC	V0.2/2019.06.21<br>V0.3/2019.06.28	NA	Minor edits and corrections, addition of IND number, and clarification of data quality assurance procedures.
Original IRB Approved Protocol	V1.0/2019.08.20	2019.10.24	Minor edits and corrections, addition of language to exclude subjects who are pregnant or breastfeeding at time of initial CAR T cell infusions, update in requirements for initial and subsequent CAR T cell infusion to exclude pregnant or breastfeeding subjects, clarification of pre-medication requirements and prohibited medication, and disease response criteria updated to include Immune-related Progressive Disease (irPD).
Amendment 1	V2.0/2020.03.04	2020.03.27	Administrative/general updates to language for clarity and consistency. Updated study committee. Clarified enrollment inclusion criteria and requirements for infusion. Clarified prohibited and allowed con meds. Removed requirement for CSF catheter collection if not feasible. Removed requirement for disease evaluations if subject has already progressed. Modified the statistical design. Added pre-CAR T cell RCL testing.
Amendment 2	V3.0/2020.09.28	2020.11.10	Administrative/general updates for clarity & consistency. Updated study committee. Clarified inclusion criteria for subjects with existing apheresis product. Clarified delays due to subject preference or surgical recovery are allowed. Clarified levetiracetam is recommended but any anti-seizure medication is allowed. Standardized language regarding post-treatment long-term follow up. Added a 30 day follow up AE/Conmed review. Clarified timing of EOT visit. Clarified CRS and Neurologic grading scale criteria.
Amendment 3	V4.0/2021.05.05	2021.05.20	Protocol revised to remove 6 course treatment limit. Clarified dysphagia exclusion 4 only applies to Arm C subjects. Removed exclusion 7 criterion, inclusion criterion 7 already specifies that anti-cancer and chemotherapy agents must be discontinued if subject does not

			already have apheresis product available for use in manufacturing. Fatigue added as an adverse event observed in Clinical Trials with CAR-T cells.<br>Revised radiation therapy washout period from ≥ 12 to ≥ 6 weeks. Clarified persistence testing will be discontinued after subject has 2 negative successive tests after their final infusion.<br>Revised 4 week (1 course) observation period to a 7 day observation period between subjects within each DR on each arm after DR1.<br>Clarified it is acceptable to use results from standard of care assessments for screening assessments. Clarified Arm C week 2 evaluations may be used for week 3 pre-infusion evaluations if performed within 2 days of the week 3 infusion. Clarified medical history is required yearly during the 15 year follow-up. Physical exam required yearly for the first 5 years of long term follow up, and beyond year 5 if subject has ongoing persistence or a prior positive RCL test. This was already required per section 7.19.1 but was inadvertently not represented in the schedule of procedures.
Amendment 4	V4.1/2021.06.08	2021.06.16	Dose Regimen figures 5-2, 5-3, 5-4, 5-6, and 5-7 corrected to reflect the dose level for courses 3 and beyond as per the narrative. Protocol V4.0 05-May-2021 figures contained errors, it was never intended for the dose level for course 3 and beyond to be revised from previous versions of the protocol except to allow treatment beyond course 6.
Amendment 5	V5.0/2021.07.21	N/A	This was submitted to the FDA but never to the IRB as the FDA immediately required changes. The 5.1 summary includes all changes from V4.1 to V5.1.
Amendment 6	V5.1/2021.08.18	2021.08.26	Sponsor name has changed to Seattle Children's Therapeutics. The term "gender" was revised to "sex". Absolute Lymphocyte inclusion criteria lowered from greater than 500cells/uL to greater than 100 cells/uL. This is in line with our liquid tumor trials and is being changed in the Solid/Brain tumor trials as we have had no issues with manufacturing CAR T cell products. Bleeding in or from the tumor, confusion, hiccups, and malaise added as risks of CAR T cell therapy to align with observed toxicities, added reference to the Investigator's Brochure. Clarified dexamethasone dose must be decrease

			for a week but only must be down to 2.5mg/m2/day on the date of infusion. Clarified disease responses assessments occur during all even courses, not just course 2, 4, 6. This was an oversight when we moved to protocol v4.0. Standardized language for persistence and clonality research tests. Revised treatment stagger requirement for subjects in DR2 and beyond from 7 days to 14 days per FDA request. Add that abnormal laboratory values or tests that are not clinically significant or do not require therapy are not considered AE.
Amendment 7	V6.0/2022.01.07	2022.02.10	Amended to add Expansion Cohort and clarify treatment plan delays & RCL testing.
Amendment 8	V7.0/2022.08.19	2022.10.06	Updated to reference current IB for safety information, add symptom management guidelines, update specifications for neurologic exam and CSF collection, update requirement for disease response assessment to as clinically indicated after study therapy, and clarify limits to treatment delays.
Amendment 9	V8.0/2022.12.01	N/A	This amendment was submitted to the FDA, but not to the IRB as further revision occurred prior to submission. All changes below to be reviewed by the IRB under next protocol version (V8.1).<br><br>Amended to update Rebecca Gardner as Sponsor responsible medical officer, to add updated information regarding CNS catheter use, to allow Arm C subjects to receive a higher DL than assigned in Courses 3 and beyond if a subsequent DR had been cleared for escalation or for selection as MTDR, to clarify allowable treatment delays and corresponding pre-/post-infusion visit requirements, to allow subjects to undergo repeat apheresis after having initiated CAR T cell study therapy in order to manufacture additional product, to add PT and PTT as required CRS labs, to include additional suggested interventions for symptoms related to non-CRS toxicity, and to update the 30 day follow-up visit to 28 day follow-up visit.
Amendment 10	V8.1/2023.02.10	2023.04.26	Amended to remove post-infusion study visits as a protocol required procedure in Course 3 and beyond, and to remove Week 2 study visits as a protocol required procedure for Arm C subjects in Course 3 and beyond.
Amendment 11	V9.0/2023.10.10	2023-12-01	Addition of tumor inflammation-associated neurotoxicity (TIAN) definition, the timing of

			on study disease evaluation MRIs was updated to as clinically indicated, and the administered dose of CAR T-cell therapy may be within +/- 10% of the protocol prescribed dose. Update to Sponsor medical officer and minor other administrative changes.
Amendment 12	V9.1/2023.12.18		Amended to transition the role of Study Chair from Nick Vitanza to Rebecca Ronsley and to update the SCTx Sponsor representative to Colleen Annesley.
