# Supplementary material for: Intracerebroventricular B7-H3-targeting CAR T cells for diffuse intrinsic pontine glioma: a phase 1 trial
Source: Nat Med. 2025 Jan 7;31(3):861–8. doi: 10.1038/s41591-024-03451-3 (PMC11922736; doi:10.1038/s41591-024-03451-3)
Supplement: Supplementary file 2 — Reporting Summary [file 41591_2024_3451_MOESM2_ESM.pdf]

nature portfolio

Corresponding author(s): Nicholas Vitanza

Last updated by author(s): 12/32024

Reporting Summary

Nature Portfolio wishes to improve the reproducibility of the work that we publish. This form provides structure for consistency and transparency in reporting. For further information on Nature Portfolio policies, see our Editorial Policies and the Editorial Policy Checklist.

Statistics

For all statistical analyses, confirm that the following items are present in the figure legend, table legend, main text, or Methods section.

n/a	Confirmed	
☐	☒	The exact sample size (*n*) for each experimental group/condition, given as a discrete number and unit of measurement
☐	☒	A statement on whether measurements were taken from distinct samples or whether the same sample was measured repeatedly
☐	☒	The statistical test(s) used AND whether they are one- or two-sided<br>*Only common tests should be described solely by name; describe more complex techniques in the Methods section.*
☐	☒	A description of all covariates tested
☐	☒	A description of any assumptions or corrections, such as tests of normality and adjustment for multiple comparisons
☐	☒	A full description of the statistical parameters including central tendency (e.g. means) or other basic estimates (e.g. regression coefficient) AND variation (e.g. standard deviation) or associated estimates of uncertainty (e.g. confidence intervals)
☐	☒	For null hypothesis testing, the test statistic (e.g. F, t, r) with confidence intervals, effect sizes, degrees of freedom and P value noted<br>*Give P values as exact values whenever suitable.*
☒	☐	For Bayesian analysis, information on the choice of priors and Markov chain Monte Carlo settings
☒	☐	For hierarchical and complex designs, identification of the appropriate level for tests and full reporting of outcomes
☒	☐	Estimates of effect sizes (e.g. Cohen's *d*, Pearson's *r*), indicating how they were calculated

Our web collection on statistics for biologists contains articles on many of the points above.

Software and code

Policy information about availability of computer code

Data collection	BD LSRFortessa, MSD MESO QuickPlex SQ 120MM, Li-COR Odyssey Clx, TopCount Scintillation Counter
Data analysis	FlowJo v.10, R v4.4, emmeans (1.10.2), ggplot2 (3.5.1), and EnhancedVolcano (1.22.0).

For manuscripts utilizing custom algorithms or software that are central to the research but not yet described in published literature, software must be made available to editors and reviewers. We strongly encourage code deposition in a community repository (e.g. GitHub). See the Nature Portfolio guidelines for submitting code & software for further information.

Data

Policy information about availability of data

All manuscripts must include a data availability statement. This statement should provide the following information, where applicable:

- Accession codes, unique identifiers, or web links for publicly available datasets
- A description of any restrictions on data availability
- For clinical datasets or third party data, please ensure that the statement adheres to our policy

All requests for raw and analyzed data and materials should be made to Dr. Jason Wendler. Requests will be promptly reviewed by the intellectual property office of Seattle Children's Research Institute to verify if the request is subject to any intellectual property or confidentiality obligations. Raw preclinical and clinical data is stored at Seattle Children’s with indefinite appropriate backup. Patient-related data not included in the paper were generated as part of clinical trials and may be patient to patient confidentiality. Any data and materials that can be shared will be released via a Material Transfer Agreement.

Research involving human participants, their data, or biological material

Policy information about studies with human participants or human data. See also policy information about sex, gender (identity/presentation), and sexual orientation and race, ethnicity and racism.

Reporting on sex and gender	Patients were eligible regardless of sex and gender.
Reporting on race, ethnicity, or other socially relevant groupings	Patients were eligible regardless of race and ethnicity.
Population characteristics	Characteristics are listed in Table 1
Recruitment	Patients were recruited through physician and self-referral. Patients were recruited from both academic and community health centers. Patients received no financial compensation to enroll though bias may exist in the fact families required the means to travel to Seattle and stay locally during a portion of therapy.
Ethics oversight	This study was conducted in accordance with FDA and International Conference on Harmonisation Guidelines for Good Clinical Practice, the Declaration of Helsinki, and applicable institutional review board requirements, including study protocol approval by the Seattle Children's Institutional Review Board.

Note that full information on the approval of the study protocol must also be provided in the manuscript.

Field-specific reporting

Please select the one below that is the best fit for your research. If you are not sure, read the appropriate sections before making your selection.

☒ Life sciences ☐ Behavioural & social sciences ☐ Ecological, evolutionary & environmental sciences

For a reference copy of the document with all sections, see nature.com/documents/nr-reporting-summary-flat.pdf

Life sciences study design

All studies must disclose on these points even when the disclosure is negative.

Sample size	Sample size was based on the phase 1 3+3 design. The MTDR was defined as the highest DR with at least six DLT-evaluable subjects and a cumulative DLT rate during Courses 1 and 2 below 34%.
Data exclusions	There was no excluded data.
Replication	Cytokine assays were performed in duplicate
Randomization	No randomization was performed as this was a non-randomized phase 1 clinical trial.
Blinding	No blinding was performed as this was a non-blinded phase 1 clinical trial.

Reporting for specific materials, systems and methods

We require information from authors about some types of materials, experimental systems and methods used in many studies. Here, indicate whether each material, system or method listed is relevant to your study. If you are not sure if a list item applies to your research, read the appropriate section before selecting a response.

Materials & experimental systems

n/a	Involved in the study	
☐	☒	Antibodies
☒	☐	Eukaryotic cell lines
☒	☐	Palaeontology and archaeology
☒	☐	Animals and other organisms
☐	☒	Clinical data
☒	☐	Dual use research of concern
☒	☐	Plants

Methods

n/a	Involved in the study	
☒	☐	ChIP-seq
☐	☒	Flow cytometry
☐	☒	MRI-based neuroimaging

Antibodies

Antibodies used	FcR Blocking Reagent human (Miltenyi, cat#130-059-901, RRID:AB_2892112); Brilliant Stain Buffer (BD, cat#563794, RRID:AB_2869750); FVS520 (FITC, BD, cat#564407, RRID:AB_2869573); FVS510 (FVS510, BD, cat#564406, RRID:AB_2869572); CD36 (FITC, BD, cat#555454, RRID:AB_2291112); CD3 (V450, BD, cat#652356,RRID:AB_2868395); CD3 (FITC, BD, cat#555332, RRID:AB_395739); CD4 (BV605, BD, cat#562658, RRID:AB_2744420); CD4 (R718, BD, cat#567092, RRID:AB_2916435); CD8 (PerCP-Cy5.5, BD, cat#560662, RRID:AB_1727513); CD8 (BV605, BD, cat#564116, RRID:AB_2869551); Cetuximab (APC, BD, custom, RRID:AB_2459632).
Validation	Cetuximab-APC antibodies were custom-conjugated by BD and validated in-house by flow cytometry. Titration experiments were performed using EGFRt expressing H9 cell lines or T cells transduced with research-grade vectors representative of other clinical trials managed by Seattle Children's. All other antibodies are validated in a similar way via positive staining on cell lines by flow cytometry mirroring manufacture validation.

Clinical data

Policy information about clinical studies
All manuscripts should comply with the ICMJE guidelines for publication of clinical research and a completed CONSORT checklist must be included with all submissions.

Clinical trial registration	NCT04185038
Study protocol	The clinical trial protocol is provided.
Data collection	BrainChild-03 began accrual on November 22 2019. Clinical data through November 13 2024 is included. The first reported patient was enrolled in August 2020 and the last reported patient was enrolled in April 2023. Study activities were conducted at Seattle Children's.
Outcomes	The primary objectives were: To assess the feasibility of CNS locoregional adoptive therapy with autologous CD4+ and CD8+ T cells lentivirally transduced to express a B7-H3-specific CAR and EGFRt, delivered by an indwelling catheter into the tumor cavity or ventricular system in children and young adults with DIPG, DMG, or recurrent/refractory CNS tumors; To assess the safety of CNS locoregional adoptive therapy with autologous CD4+ and CD8+ T cells lentivirally transduced to express a B7-H3-specific CAR and EGFRt, delivered by an indwelling catheter into the tumor cavity or ventricular system in children and young adults with DIPG, DMG, or recurrent/refractory CNS tumors; To establish the tolerability of a fractionated CNS-delivered B7-H3 CAR T cell infusion schedule employing intra-subject dose escalation in children and young adults with DIPG, DMG, or recurrent/refractory CNS tumors; To define the maximally tolerated dose (MTD) and recommended Phase 2 dose regimen (RP2DR) of CNS-delivered fractionated B7-H3 CAR T cell infusions. The secondary objectives were: The secondary objectives are: To assess B7-H3 CAR T cell distribution within the cerebrospinal fluid (CSF) and the extent to which B7-H3 CAR T cells egress into the peripheral circulation; and To assess disease response to B7-H3 CAR T cell locoregional therapy in children and young adults with DIPG, DMG, or recurrent/refractory CNS tumors.

Plants

Seed stocks	n/a
Novel plant genotypes	n/a
Authentication	n/a

Flow Cytometry

Plots

Confirm that:

- [x] The axis labels state the marker and fluorochrome used (e.g. CD4-FITC).
- [x] The axis scales are clearly visible. Include numbers along axes only for bottom left plot of group (a 'group' is an analysis of identical markers).
- [x] All plots are contour plots with outliers or pseudocolor plots.
- [x] A numerical value for number of cells or percentage (with statistics) is provided.

Methodology

Sample preparation

Patient cerebrospinal fluid (CSF) samples were collected via lumbar puncture or ventricular catheter and kept at 4°C until processing. The samples underwent serial centrifugation: first at 250xg for 10 minutes to remove cells, followed by a final centrifugation at 10,000xg for 10 minutes to remove any remaining debris. The cell-free supernatant was then aliquoted and cryopreserved at -80°C. CSF immunophenotyping: Immunophenotyping of surface markers on cells isolated from the CSF specimens was conducted using standard staining protocols followed by flow cytometry analysis.

Instrument

Identify the instrument used for data collection, specifying make and model number.

Software

FlowJo

Cell population abundance

Samples with lymphocytes count under the limit of quantitation (LOQ) requirement for the assay were excluded from reporting. CAR T cell detection status is determined by a combination of at least one detectable EGFRt+ cell count in the sample, as well as the level of Lymphocytes/EGFRt+% cell in the sample to be above the pre-defined limit of detection (LOD) for the assay.

Gating strategy

Gating strategy used for flow-based CAR detection in CSF. Selection of the singlet, viable [CD36-] lymphocyte population was performed prior to T cell gating and examination of the EGFRt+ CART cells and CD4/CD8 expression.

☒ Tick this box to confirm that a figure exemplifying the gating strategy is provided in the Supplementary Information.

Magnetic resonance imaging

Experimental design

Design type

Clinical studies

Design specifications

Clinical studies

Behavioral performance measures

n/a

Acquisition

Imaging type(s)

clinical studies

Field strength

1.5T and 3T

Sequence & imaging parameters

Routine clinical MRI consisting of Brain = sagittal T1 MPRAGE at 1 mm with multiplanar reformats without and with contrast; axial and coronal T2; axial FLAIR; axial DWI with ADC maps; axial SWI. Spine = post-contrast axial and sagittal T1

Area of acquisition

Brain and spinal cord

Diffusion MRI ☒ Used ☐ Not used

Parameters

Routine clinical DWI

Preprocessing

Preprocessing software

n/a

Normalization

n/a

Normalization template

n/a

Noise and artifact removal

n/a

Volume censoring

n/a

Statistical modeling & inference

Model type and settings

n/a

Effect(s) tested

n/a

Specify type of analysis: ☐ Whole brain ☐ ROI-based ☐ Both

Statistic type for inference<br>(See Eklund et al. 2016)	n/a
Correction	n/a

Models & analysis

n/a	Involved in the study
☒	☐ Functional and/or effective connectivity
☒	☐ Graph analysis
☒	☐ Multivariate modeling or predictive analysis
